# Hetero-oligomerization drives structural plasticity of eukaryotic peroxiredoxins

**DOI:** 10.1038/s41589-026-02157-6

**Published:** 2026-03-10

**Authors:** Jannik Zimmermann, Lukas Lang, Julia Malo Pueyo, Mareike Riedel, Khadija Wahni, Dylan Stobbe, Laura Leiskau, Elham Aref, Christopher Lux, Steven Janvier, Didier Vertommen, Svenja Lenhard, Frank Hannemann, Sudharshini Thangamuragan, Helena Castro, Volkhard Helms, Ana Maria Tomas, Johannes M. Herrmann, Armindo Salvador, Timo Mühlhaus, Jan Riemer, Joris Messens, Marcel Deponte, Bruce Morgan

**Affiliations:** 1 Institute of Biochemistry, Center for Human and Molecular Biology (ZHMB), Saarland University, Saarbrücken, Germany; 2 Faculty of Chemistry, Comparative Biochemistry, University of Kaiserslautern-Landau (RPTU), Kaiserslautern, Germany; 3 VIB–VUB Center for Structural Biology, VIB, Brussels, Belgium; 4 Brussels Center for Redox Biology, Brussels, Belgium; 5 Structural Biology Brussels, Vrije Universiteit Brussel, Brussels, Belgium; 6 Redox Metabolism, Institute for Biochemistry, and Cologne Excellence Cluster on Cellular Stress Responses in Aging-Associated Diseases (CECAD), University of Cologne, Cologne, Germany; 7 Computational Systems Biology, University of Kaiserslautern-Landau (RPTU), Kaiserslautern, Germany; 8 De Duve Institute, MASSPROT Platform, UCLouvain, Brussels, Belgium; 9 Cell Biology, University of Kaiserslautern-Landau (RPTU), Kaiserslautern, Germany; 10 Center for Bioinformatics, Saarland University, Saarbrücken, Germany; 11 i3S—Instituto de Investigação e Inovação em Saúde, Universidade do Porto, Porto, Portugal; 12 ICBAS—Instituto de Ciências Biomédicas Abel Salazar, Universidade do Porto, Porto, Portugal; 13 CNC-UC—Centre for Neuroscience and Cell Biology, University of Coimbra, Coimbra, Portugal; 14 CiBB—Centre for Innovative Biomedicine and Biotechnology, University of Coimbra, Coimbra, Portugal; 15 Coimbra Chemistry Center–Institute of Molecular Sciences (CQC–IMS), University of Coimbra, Coimbra, Portugal; 16 Institute for Interdisciplinary Research, University of Coimbra, Coimbra, Portugal

**Keywords:** Enzymes, Model fungi, Structural biology, Protein folding

## Abstract

Peroxiredoxins are thiol peroxidases, which detoxify peroxides, relay redox signals and act as chaperones. In eukaryotes, multiple peroxiredoxin-1 (Prx1)/AhpC-type isoforms frequently co-exist in the same subcellular compartment, yet have been assumed to assemble only as homo-oligomeric complexes. Here we show that hetero-oligomerization is a conserved and functionally relevant property of Prx1/AhpC-type peroxiredoxins. Using biochemical reconstitution, native mass photometry, electron microscopy and live-cell assays, we demonstrate formation of heterodimers and heterodecamers, with diverse subunit stoichiometries, in peroxiredoxin pairs from different eukaryotic kingdoms. In *Saccharomyces cerevisiae*, oxidative challenge induces Tsa1–Tsa2 heterodecamerization with substoichiometric Tsa2 incorporation sufficing to stabilize the decameric state. Functional hetero-oligomers are also observed forming among human, plant and *Leishmania* peroxiredoxins. Our findings provide new insights into peroxiredoxin structural plasticity with broad implications for redox biology, stress responses and cellular adaptation, and also challenge the long-held paradigm of peroxiredoxin homo-oligomerization.

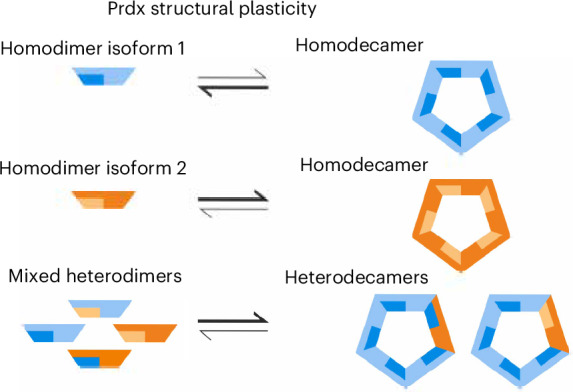

## Abstract (web summary)

Peroxiredoxins from diverse organisms were found to assemble into hybrid complexes, not just identical ones. These mixed assemblies reshape structure and stability, challenging a long-held view of peroxiredoxin assembly in cells.

## Main

**Extended Data Fig. 1:**
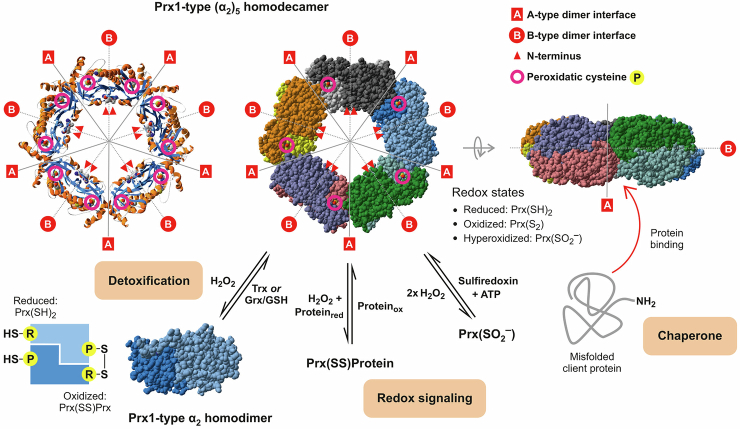
The oligomeric state of peroxiredoxins is closely linked to their functions and is affected by numerous factors. Prx1/AhpC-type peroxiredoxins (Prx) exist in a dynamic equilibrium between dimeric and decameric forms of the (α_2_)₅-type depicted as a ribbon diagram (top left), space-filling model (top middle), and space-filling side-view (top right). The oligomeric assembly is formed through A-type (dimer–dimer) and B-type (intradimer) interfaces. Prx dimers (bottom left) transition between reduced (Prx(SH)_2_) and oxidized (Prx(SS)) forms via reaction with H_2_O_2_, followed by reduction facilitated by thioredoxins (Trx) or glutaredoxins (Grx). Hyperoxidation leads to Prx(SO_2_) formation, reversible via sulfiredoxin (Srx) in an ATP-dependent reaction. The function of Prx1–AhpC-type peroxiredoxins is intricately coupled to their oligomeric state and includes peroxide detoxification, redox signaling, and chaperone activity.

Peroxiredoxins are highly efficient enzymes found in nearly all living organisms. As some of the most abundant cellular proteins, they have key roles in peroxide scavenging, redox signaling and as molecular chaperones^1–5^ (Extended Data Fig. 1). These versatile enzymes are classified into various groups based on their enzymatic mechanisms or structural features.

Peroxiredoxin-1 (Prx1)/AhpC-type peroxiredoxins are typically found in a dynamic equilibrium between homodimers and homodecamers^3,6,7^. Homodimers interact across the B-interface, whereas homodecamers are of the (α_2_)_5_ type, in which five dimers associate through their A-type dimer interfaces^8^(Extended Data Fig. 1). The dimer–decamer equilibrium is influenced by factors including protein concentration, the redox state of the catalytic cysteine residues, pH and various post-translational modifications^6,9–16^. All characterized Prx1/AhpC-type peroxiredoxins are mechanistically typical 2-Cys peroxiredoxins, containing a peroxidatic cysteine (C_P_) and a resolving cysteine (C_R_) on each subunit, both of which are essential for catalysis^8,17^. Dimers arise from a ‘head-to-tail’ arrangement of subunits, which enables intermolecular disulfide bond formation between the C_P_ and C_R_ of opposing monomers at both ends of the dimer (Extended Data Fig. 1).

Many organisms harbor two or more Prx1/AhpC-type peroxiredoxins, often with two highly homologous isoforms located in the same subcellular compartment. In the budding yeast *Saccharomyces cerevisiae*, Tsa1 and Tsa2 share 86% sequence identity and are both cytosolic^18–20^. Likewise, human PRDX1 and PRDX2 and *Leishmania infantum Li*PRX1 and *Li*PRX2 co-exist in the cytosol and share 78% and 87% sequence identity, respectively^21–23^. Finally, in *Arabidopsis thaliana*, *At*BAS1A and *At*BAS1B, sharing 96% sequence identity (excluding the chloroplast-targeting transit peptides), both reside in the chloroplast stroma. Nonetheless, these isoforms frequently exhibit marked differences in biophysical and biochemical properties, including their dimer–decamer equilibria, isoelectric points, enzyme kinetics and susceptibility to various post-translational modifications^10,24–28^.

Despite their high sequence identity and very similar quaternary structures, Prx1/AhpC-type peroxiredoxins have traditionally been considered to assemble exclusively into homo-oligomeric complexes. The first challenge to this view emerged from the characterization of peroxiredoxin-based H_2_O_2_ sensors^29,30^. We showed that the genetically encoded roGFP2-Tsa2ΔC_R_ probe forms enzymatically active hetero-oligomeric complexes with endogenous Tsa1 in yeast^29^. More recently, human PRDX1 and PRDX2 were also shown to form hetero-oligomers in vitro, although functionality, activity and in vivo relevance were not assessed^31^. These observations prompted us to investigate whether peroxiredoxin hetero-oligomerization is limited to specialized experimental contexts or represents a broader, biologically relevant feature of eukaryotic Prx1/AhpC-type peroxiredoxins.

In this study, we demonstrate that eukaryotic peroxiredoxins can assemble into hetero-oligomers with a broad range of subunit stoichiometries, with hetero-oligomer formation typically modulating the dimer–decamer equilibrium. In yeast, hetero-oligomerization between Tsa1 and Tsa2 is inducible upon oxidative challenge, coincident with the upregulation of *TSA2* expression. Even substoichiometric incorporation of Tsa2 strongly stabilizes the decameric state. We detect human PRDX1–PRDX2 hetero-oligomers in HEK293T cells and show that *Arabidopsis* and *Leishmania* peroxiredoxins likewise assemble into functional hetero-oligomers. Given the link between peroxiredoxin oligomeric state and function, hetero-oligomerization likely represents a widespread and conserved regulatory mechanism that fine-tunes peroxiredoxin activity across different compartments, cell types and species throughout the domain Eukaryota.

## Results

### Tsa1 and Tsa2 form hetero-oligomers in *Escherichia coli*

**Fig. 1:**
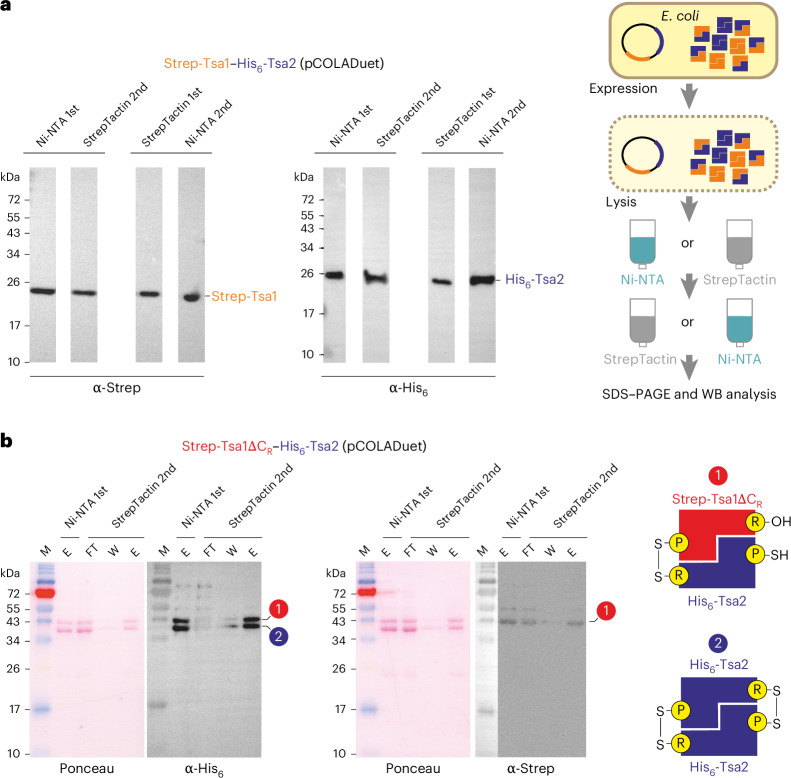
Strep-Tsa1 and His_6_-Tsa2 form hetero-oligomers in *E. coli.* **a**, Purification scheme and WB analysis of the eluates from tandem-affinity copurifications of Strep-Tsa1 and His_6_-Tsa2 with Ni-NTA agarose, followed by StrepTactin agarose or vice versa. Eluate samples were separated by reducing SDS–PAGE. The calculated molecular masses of Strep-Tsa1 and His_6_-Tsa2 are 22.9 kDa and 23.8 kDa, respectively. Uncropped blots are shown in Supplementary Fig. 1. **b**, Tandem-affinity copurification of Strep-Tsa1ΔC_R_ and His_6_-Tsa2. Protein samples were treated with NEM to block free thiols and separated by nonreducing SDS–PAGE to preserve intersubunit disulfide bonds, as shown in **b**. In **a** and **b**, gels are representative of those obtained during three experimental repeats, each yielding similar results. M, marker; FT, flow through; W, wash; E, eluate. Source data

We first asked whether Tsa1 and Tsa2 can form hetero-oligomers when purified from *E. coli* (Fig. 1 and Supplementary Fig. 1). Recombinant N-terminally His_6_-tagged or Strep-tagged Tsa1 and His_6_-tagged Tsa2 were produced and purified individually (Supplementary Fig. 1a) and then mixed at a 1:1 molar ratio. After a tandem-affinity purification using Ni-NTA agarose followed by StrepTactin agarose beads, no interaction between Strep-Tsa1 and His_6_-Tsa2 was detected. This suggests that individually purified recombinant Tsa1 and Tsa2 form stable homo-oligomeric complexes and do not exchange subunits under these conditions.

We then coexpressed genes encoding Strep-Tsa1 and His_6_-Tsa2 from a single plasmid in *E. coli* and performed tandem-affinity purifications with Ni-NTA agarose followed by StrepTactin agarose, or vice versa. Under these conditions, Strep-Tsa1 and His_6_-Tsa2 were successfully copurified irrespective of the order of the tandem-affinity purification, supporting an interaction between recombinant Tsa1 and Tsa2 in *E. coli* (Fig. 1a and Supplementary Fig. 1b,c). Semiquantitative western blotting calibrated with individually purified proteins revealed similar levels of copurified Tsa1 and Tsa2, suggesting a ~1:1 ratio of Strep-Tsa1 and His_6_-Tsa2 (Supplementary Fig. 1d).

To rule out other possibilities for copurification, for example, the formation of heterogenous stacks of homodecameric complexes, we performed tandem-affinity purification with the resolving cysteinyl mutant Strep-Tsa1ΔC_R_, which cannot form disulfide-linked Tsa1ΔC_R_–Tsa1ΔC_R_ homodimers, and His_6_-Tsa2, which can form either Tsa2–Tsa2 homodimers or disulfide-linked Tsa1ΔC_R_–Tsa2 heterodimers (Fig. 1b). Nonreducing SDS–PAGE and western blotting detected two disulfide-linked dimer species for His_6_-Tsa2, whereas a single disulfide-linked dimer species was detected for Strep-Tsa1ΔC_R_, indicating that the proteins form heterodimers through the B-type interface (Fig. 1b). The formation of Tsa1–Tsa2 heterodimers was also supported by in silico analysis using HADDOCK-based molecular docking (Supplementary Fig. 2). In conclusion, recombinant yeast Tsa1 and Tsa2 readily interact through their B-type interface, and probably also through their A-type interface, forming hetero-oligomeric complexes.

### Tsa1 and Tsa2 form decamers with varied stoichiometries

**Fig. 2:**
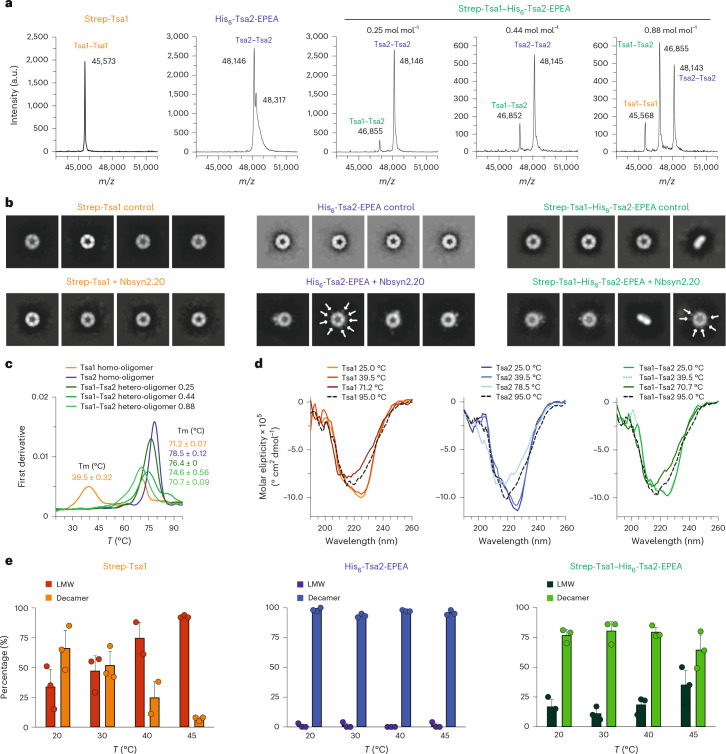
Tsa1–Tsa2 hetero-oligomer formation stabilizes the decameric state. **a**, MALDI-TOF MS analysis of Strep-Tsa1 and His_6_-Tsa2-EPEA homo-oligomers and Strep-Tsa1–His_6_-Tsa2-EPEA hetero-oligomers. The Tsa1:Tsa2 molar ratio of each hetero-oligomer sample is displayed above each panel. The signal intensity (a.u.) is plotted against different *m*/*z* ratios (*n* = 2 independent experimental repeats). Expected masses are listed in Supplementary Table 1. **b**, Negative staining EM analysis of Nbsyn2.20 binding to the 0.88 mol mol^−1^ Strep-Tsa1–His_6_-Tsa2-EPEA heterodecamer at multiple positions. Protein particles appear bright against the 2% uranyl-acetate stain, revealing a characteristic decameric ‘donut-like’ structure. Nbsyn2.20 positions are marked with white arrows. Scale bars, 200 Å. Images are representative of those obtained during three experimental repeats, each yielding similar results. **c**, NanoDSF analysis of Strep-Tsa1 and His_6_-Tsa2-EPEA homo-oligomers and Strep-Tsa1–His_6_-Tsa2-EPEA hetero-oligomers. Temperature was increased to 100 °C (2 °C min^−1^; *n* = 3 technical replicates). Tm, melting temperature. **d**, CD analysis of Strep-Tsa1 and His_6_-Tsa2-EPEA homo-oligomers, and Strep-Tsa1–His_6_-Tsa2-EPEA hetero-oligomer samples (*n* = 5 technical replicates). **e**, Mass photometry analysis of Strep-Tsa1, His_6_-Tsa2-EPEA and the 0.88 mol mol^−1^ Strep-Tsa1–His_6_-Tsa2-EPEA sample at 20 °C, 30 °C, 40 °C and 45 °C, respectively. Data were acquired for 60 s, and counts of individual molecules were plotted against their molecular weight (*n* = 3 technical replicates with separate aliquots of proteins obtained from 1 purification). Data are presented as mean ± s.d. Source data

We then sought to visualize Tsa1–Tsa2 hetero-oligomers by negative-stain electron microscopy (EM). To this end, we coexpressed Strep-Tsa1 and His_6_-Tsa2-EPEA, in which Tsa2 additionally contains a C-terminal EPEA epitope that binds with high affinity to the nanobody Nbsyn2.20 (refs. ^32,33^). Recombinant Strep-Tsa1–His_6_-Tsa2-EPEA hetero-oligomers were produced and purified using tandem Ni-NTA and StrepTactin affinity chromatography, and three different fractions were collected for analysis. Strep-Tsa1 homo-oligomers and His_6_-Tsa2-EPEA homo-oligomers were purified separately as controls (Supplementary Fig. 3a–d). Native PAGE analysis showed that the Tsa1–Tsa2 hetero-oligomers run at a mass consistent with a decamer, between the bands observed for the Tsa1 and Tsa2 homo-oligomers (Supplementary Fig. 3e). Matrix-assisted laser desorption/ionization time-of-flight (MALDI-TOF) mass spectrometry (MS) analysis revealed that all Tsa1–Tsa2 hetero-oligomers contain disulfide-linked dimers, consistent with the lack of a reducing agent in the purification protocol (Fig. 2a). Notably, all three hetero-oligomer populations contained disulfide-linked Tsa1–Tsa2 heterodimers as well as Tsa2–Tsa2 homodimers. One of the hetero-oligomer fractions also contained disulfide-linked Tsa1–Tsa1 homodimers (Fig. 2a). All dimers were reducible with dithiothreitol (DTT), and no contamination with *E. coli* AhpC was detected (Supplementary Fig. 4 and Supplementary Table 1). Liquid chromatography (LC)–MS/MS analysis of the three hetero-oligomer populations revealed distinct Tsa1:Tsa2 molar ratios; 0.25 mol mol^−1^ (most Tsa2), 0.42 mol mol^−1^ and 0.88 mol mol^−1^ (least Tsa2; PRIDE accession PXD060819), indicating hetero-oligomer populations with different subunit stoichiometries. Finally, negative-stain EM imaging of Tsa1 and Tsa2 homo-oligomers and the 0.88 mol mol^−1^ Tsa1–Tsa2 hetero-oligomer fraction revealed donut-shaped particles in all samples, consistent with decamers of similar diameter (Supplementary Fig. 5).

We then used the Nbsyn2.20 nanobody to visualize the position of Tsa2 within the hetero-oligomers. We first assessed the specificity of Nbsyn2.20 binding to the EPEA tag. Biolayer interferometry (BLI) and mass photometry confirmed specific interaction with EPEA-tagged proteins, with no binding observed to Tsa1 homo-oligomers (Supplementary Fig. 6a). Mass photometry further revealed that at least two Nbsyn2.20 molecules bind to a Tsa1–Tsa2 heterodecamer. Multiple populations of Tsa1–Tsa2 heterodecamers were detected, as reflected by an asymmetric molecular-weight peak with a tail. The Tsa2 homo-oligomer control showed various populations with different numbers of Nbsyn2.20 molecules bound, likely due to the dynamic nature of the interaction (Supplementary Fig. 6b). Tsa1 homodecamers did not bind Nbsyn2.20, confirming the specificity of the nanobody–EPEA interaction.

Finally, negative-stain EM imaging of Tsa1–Tsa2 heterodecamers in the presence of Nbsyn2.20 showed a diverse population with different numbers of nanobodies binding to different particles. This is likely explained by the existence of different stoichiometries of Tsa1 and Tsa2 in solution (Fig. 2b). In agreement with the BLI and mass photometry analyses, no interaction was observed between Tsa1 homo-oligomers and Nbsyn2.20, whereas Tsa2 homo-oligomers displayed up to ten Nbsyn2.20 molecules bound at distinct positions (Fig. 2b). In summary, our structural analysis of recombinant Tsa1–Tsa2 hetero-oligomers reveals a donut-shaped organization in which Tsa1 and Tsa2 likely assemble into multiple, distinct, heterodecameric species.

### Tsa2 stabilizes the decameric state of the hetero-oligomers

We then evaluated the decamer stability and dimer–decamer equilibrium of Tsa1–Tsa2 hetero-oligomers in comparison to their homo-oligomeric counterparts, focusing here on the disulfide-linked oxidation form. NanoDSF analysis of the Tsa1 homo-oligomer revealed two inflection points at 39.5 °C and 71.2 °C (Fig. 2c). In contrast, Tsa2 homo-oligomers displayed only one inflection point at 78.5 °C. The three purified Tsa1–Tsa2 hetero-oligomer populations also presented only one inflection point at 76.4 °C (0.25 mol mol^−1^ population), 74.6 °C (0.42 mol mol^−1^ population) and 70.7 °C (0.88 mol mol^−1^ population; Fig. 2c). These data indicate that increasing Tsa2 content enhances oligomer stability, as reflected by progressively higher inflection temperatures. We then performed circular dichroism (CD) measurements for the Tsa1 homo-oligomer, Tsa2 homo-oligomer and the 0.88 mol mol^−1^ Tsa1–Tsa2 hetero-oligomer sample at temperatures of 25 °C, 39.5 °C, the respective inflection point temperature for each sample and 95 °C (Fig. 2d). The CD data showed no change in secondary structure at 39.5 °C in any sample. For all three proteins, the second inflection point near 75 °C in Fig. 2c corresponds to a loss of some secondary structural features; however, none of the protein samples fully denatured at any temperature tested, suggesting that the Tm values lie above 95 °C (Fig. 2d).

To gain further insight into the molecular basis of the inflection points observed by nanoDSF, we again used mass photometry (Fig. 2e and Supplementary Table 2). Measurements were performed at 20 °C, 30 °C, 40 °C and 45 °C for the Tsa1 homo-oligomer, Tsa2 homo-oligomer and the 0.88 mol mol^−1^ Tsa1–Tsa2 hetero-oligomer samples, respectively. The relative abundance (%) of low-molecular-weight (LMW) species and decamers was quantified under each condition. Tsa1 showed a clear loss of the decameric state and dissociation to dimers with increasing temperature, with a pronounced shift at 40 °C. This supports the interpretation that the 39.5 °C inflection point observed by nanoDSF reflects dissociation of Tsa1 homodecamers into homodimers. In contrast, the Tsa2 homo-oligomer and 0.88 mol mol^−1^ Tsa1–Tsa2 hetero-oligomer samples displayed far less dissociation into LMW oligomers (Fig. 2e).

To examine potential effects of peptide tags on oligomer stability, we used mass photometry to monitor the temperature-dependent dimer–decamer ratios of Tsa1, Tsa2 and Tsa1–Tsa2 hetero-oligomers with N-terminal and C-terminal green fluorescent protein (GFP) tags (Supplementary Fig. 7 and Supplementary Table 2). An N-terminal GFP tag destabilized the decameric state, particularly for Tsa1. Nonetheless, the increased decamer stability of the hetero-oligomeric complexes, and especially for Tsa2, was consistently observed regardless of tag identity or tag position. Notably, in the His_6_-GFP-TEV-Tsa2 homo-oligomer and in the Tsa1–Tsa2-TEV-GFP-His_6_ hetero-oligomer samples, we detected some AhpC contamination (Supplementary Figs. 8 and 9; LC–MS data—PRIDE accession PXD060819).

In summary, our data support that the Tsa1 homodecamer is less stable than the Tsa2 homodecamer, consistent with previous reports^27^. Intriguingly, the incorporation of substoichiometric amounts of Tsa2 strongly stabilizes the decameric state of the resultant Tsa1–Tsa2 heterodecamers, suggesting that hetero-oligomerization may serve as a mechanism for regulating peroxiredoxin oligomeric state dynamics.

### H_2_O_2_-induced Tsa1–Tsa2 assembly stabilizes decamers in yeast

**Fig. 3:**
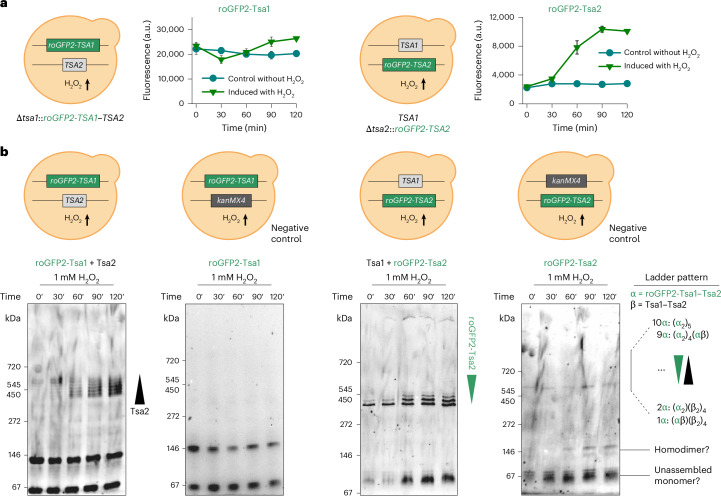
Hetero-oligomerization is inducible in yeast and promotes decamer stabilization. **a**, Graph showing the GFP fluorescence intensity of roGFP2-Tsa1 and roGFP2-Tsa2 constructs in yeast cells at the indicated time points after the treatment with 1 mM exogenous H_2_O_2_. The constructs were expressed from genes integrated into the *TSA1* and *TSA2* genomic loci, respectively, remaining under the control of the native promoters and 3′ untranslated regions. Experiments were repeated thrice with independent yeast cultures. Data represent mean ± s.d. **b**, Clear native PAGE gels, monitored for GFP fluorescence, of lysates taken from the cultures of the indicated yeast strains at the indicated time points following the addition of 1 mM exogenous H_2_O_2_. Wedges indicate hetero-oligomers containing an increasing number of Tsa2 subunits (first panel from the left) and an increasing number of roGFP2-Tsa2 subunits (third panel from the left). Two further experimental repeats are shown in Supplementary Fig. 10. Source data

Under standard growth conditions, Tsa1 is highly abundant in yeast cells, whereas Tsa2 levels are much lower^34^. However, *TSA2* expression is known to be inducible under oxidative challenge^35,36^. To investigate whether Tsa1 and Tsa2 hetero-oligomers form when expressed from their native promoters, we replaced either *TSA1* or *TSA2* at their endogenous genomic loci with roGFP2-tagged versions, that is, *RoGFP2-TSA1* and *RoGFP2-TSA2*, while maintaining the native promoter and 3′ untranslated regions. Fluorescence measurements confirmed that the roGFP2-Tsa1 level was approximately tenfold higher than that of roGFP2-Tsa2 (Fig. 3a). Upon exposure to 1 mM H_2_O_2_, the roGFP2-Tsa1 fluorescence remained unchanged, whereas the roGFP2-Tsa2 fluorescence increased approximately fivefold within 90 min, confirming induction of *TSA2* expression but not *TSA1* upon oxidative challenge (Fig. 3a).

To further explore whether *TSA2* induction promotes hetero-oligomer formation, we performed clear native gel electrophoresis. Under standard conditions, roGFP2-Tsa1 migrated predominantly as a dimer. Within 30 min after H_2_O_2_ addition to Δ*tsa1*::*RoGFP2-TSA1* cells, we observed additional bands at a molecular mass consistent with roGFP2-Tsa1–Tsa2 heterodecamers, and these bands intensified during the time-course of the experiment (Fig. 3b and Supplementary Fig. 10). We observed a ladder pattern of decamer bands, consistent with the formation of roGFP2-Tsa1–Tsa2 heterodecamers containing different subunit stoichiometries (Fig. 3b). No decameric bands were observed in Δ*tsa1*::*RoGFP2-TSA1* Δ*tsa2* cells in which *TSA2* is deleted (Fig. 3b). As an additional control, we monitored the hyperoxidation status of the peroxidatic cysteines, which could potentially stabilize the decameric state (Supplementary Fig. 11a). No hyperoxidation was detected under our experimental conditions.

In Δ*tsa2*::*RoGFP2-TSA2* cells, the predominant species were decamers and a band that we interpret as unassembled monomers. Upon H_2_O_2_ treatment and the resulting induction of roGFP2-Tsa2 expression, a series of higher-molecular-mass bands appeared above the original decamer band. This suggests that as roGFP2-Tsa2 levels increase, hetero-oligomers containing increasing proportions of roGFP2-Tsa2 subunits are formed (Fig. 3b). Consistent with our previous results, we found no evidence of hyperoxidation in these cells during the time-course of this experiment (Supplementary Fig. 11b).

In conclusion, our findings demonstrate that Tsa1–Tsa2 hetero-oligomerization is inducible upon oxidative challenge and that incorporation of only one or two Tsa2 subunits is sufficient to stabilize a heterodecameric complex.

### Hetero-oligomerization allows high structural plasticity

**Extended Data Fig. 2:**
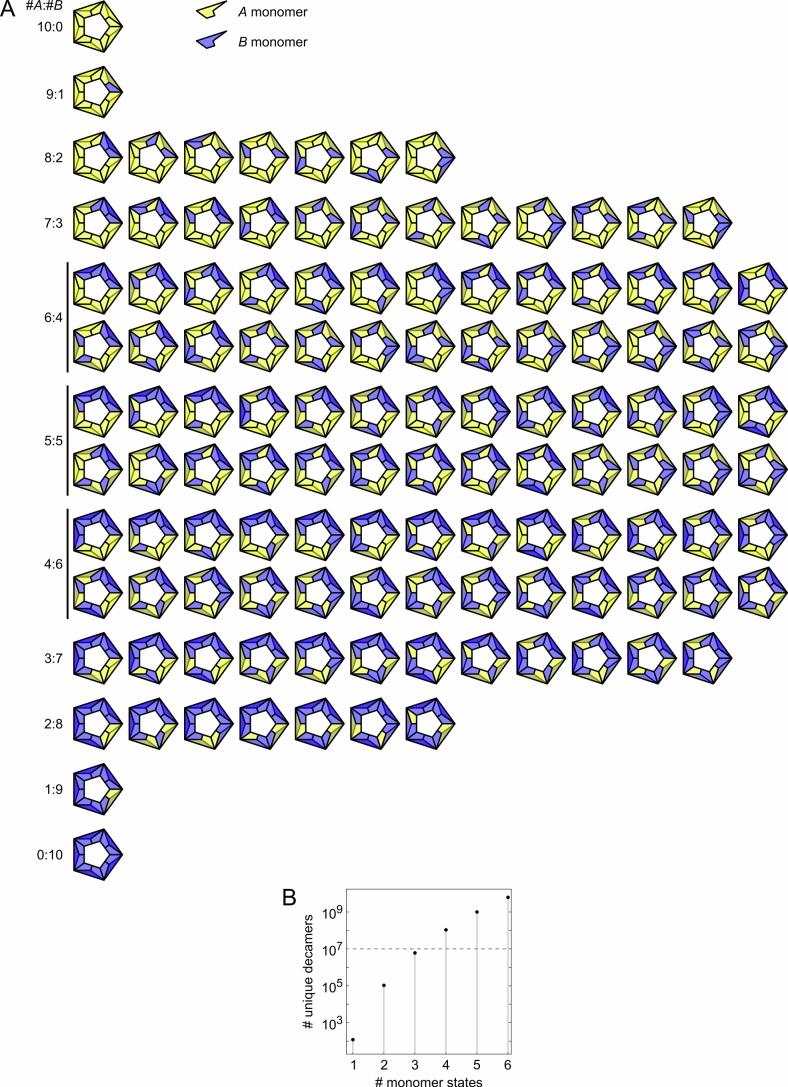
Enumeration of the distinct decamers that can be formed from two types of monomers. **a**, Distinct decamers as a function of the stoichiometric ratio between monomer types. **b**, Number of unique hetero-decamer configurations as a function of the number of possible states each monomer type can adopt. The horizontal dashed line indicates the estimated number of peroxiredoxin decamers in a typical mammalian cell. Please note the logarithmic scale on the vertical axis. Calculations are explained in the Supplementary Note.

Our data support the existence of Tsa1–Tsa2 interactions across both the A-type and B-type interfaces, prompting us to calculate the theoretical maximum number of possible different hetero-oligomeric configurations. This analysis revealed that two distinct monomer types can assemble into 120 unique decamers. These are defined as structures that are not rotations of the same decamer around the fivefold symmetry axis perpendicular to the plane of the decamer or across the five twofold symmetry axes parallel to this plane (Extended Data Fig. 2 and Supplementary Note). These 120 distinct heterodecamers include 2 distinct homodecameric structures, 2 decamers with a 1:9 or 9:1 stoichiometric ratio, 14 decamers with 2:8 or 8:2 ratios, 24 decamers with 3:7 or 7:3 ratios, 52 decamers with 4:6 or 6:4 ratios and 26 decamers with 5:5 ratio.

The potential number of distinct hetero-oligomers increases dramatically when the multiple structural states of each monomer are considered. Each monomer switches among at least three distinct structural states during the catalytic cycle, and these states multiply several-fold when known post-translational modifications are also considered^37^. Consequently, the number of possible unique heterodecamer configurations scales with the 10th power of the number of monomer states (Extended Data Fig. 2b and Supplementary Note). This number would far exceed the number of peroxiredoxin decamers present in a cell, even if each monomer type had only four states. The potential configurations increase substantially when more realistic state counts are considered. Therefore, only a very small fraction of the possible peroxiredoxin hetero-oligomer configurations can exist in a cell at any given moment.

### Tsa1–Tsa2 hetero-oligomers show homo-oligomer-like kinetics

**Fig. 4:**
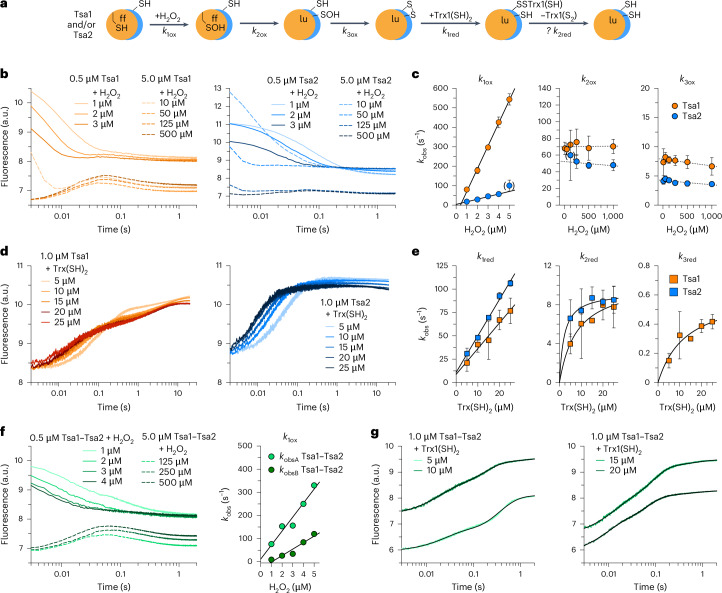
Hetero-oligomers of recombinant Tsa1 and Tsa2 have similar enzyme kinetics as compared with their homo-oligomers. **a**, Reaction scheme for the oxidation and reduction of recombinant Tsa1 and/or Tsa2. Only the dimeric enzyme species are shown for simplicity. **b**, Representative changes of tryptophan fluorescence during the H_2_O_2_-dependent oxidation of individually purified His_6_-Tsa1(SH)_2_ (left) or His_6_-Tsa2(SH)_2_ (right). **c**, Secondary plots of the observed rate constants (*k*_obs_) from exponential fits of the three reaction phases at variable H_2_O_2_ concentrations from **b**. **d**, Representative changes of tryptophan fluorescence during the yeast Trx1-dependent reduction of individually purified His_6_-Tsa1(S_2_) (left) or His_6_-Tsa2(S_2_) (right). **e**, Secondary plots of the *k*_obs_ values from exponential fits of the two or three reaction phases at variable Trx1 concentrations from **d**. **f**, Representative changes of tryptophan fluorescence during the H_2_O_2_-dependent oxidation of copurified His_6_-Tsa1(SH)_2_ and His_6_-Tsa2(SH)_2_ (left) and secondary plot of the *k*_obs_ values from exponential fits of the first reaction phase (right). **g**, Representative changes of tryptophan fluorescence during the yeast Trx1-dependent reduction of copurified His_6_-Tsa1(S_2_) and His_6_-Tsa2(S_2_) (Supplementary Fig. 1). Quadruple exponential fits (black lines) were calculated using the *k*_obs_ values of the first phase of individually purified His_6_-Tsa1 and His_6_-Tsa2 at the corresponding Trx1 concentration as input. All stopped-flow measurements were performed at pH 7.4 and 25 °C. Ten times higher enzyme concentrations were used to highlight the second and third oxidation phases (dashed lines). Data from **c** and **e** were from technical triplicates of independent biological triplicate protein purifications and measurements. Data from **f** and fits in **g** were from technical triplicates of a single measurement. Error bars in all panels represent the s.d. Rate constants are summarized in Supplementary Table 3. Source data

We recently analyzed the catalytic cycle of recombinant His_6_-Tsa1 using stopped-flow kinetic measurements, revealing three distinct reaction phases for the H_2_O_2_-dependent oxidation of reduced Tsa1, that is, Tsa1(SH)_2_, and three phases for the yeast Trx1-dependent reduction of Tsa1 disulfide, that is, Tsa1(S_2_)^38^. To compare the catalytic activity of Tsa1–Tsa2 hetero-oligomers to that of their homo-oligomeric counterparts, we analyzed individually purified His_6_-Tsa1 and His_6_-Tsa2, and Tsa1–Tsa2 hetero-oligomers (Fig. 4, Supplementary Fig. 1 and Supplementary Table 3).

As previously reported for Tsa1 (refs. ^27,38,39^), three distinct phases were observed during the oxidation of individually purified His_6_-Tsa1(SH)_2_ and His_6_-Tsa2(SH)_2_. First, a decrease in tryptophan fluorescence reflects the rapid formation of the sulfenic acid species at higher H_2_O_2_ concentrations. Second, an increase in fluorescence indicates a conformational change from the fully folded to the locally unfolded state. Third, fluorescence decreases again due to the formation of an intermolecular disulfide bond at the B-type dimer interface (Fig. 4a,b). The second-order rate constant *k*_1ox_ of 1.3 × 10^7 ^M^−1^ s^−1^ for the sulfenic acid formation in His_6_-Tsa2 was tenfold lower than that for His_6_-Tsa1, whereas the first-order rate constants (*k*_2ox_ and *k*_3ox_) for the H_2_O_2_-independent phases were similar for both enzymes (Fig. 4c and Supplementary Table 3). Control experiments with Strep-Tsa1 revealed that the Strep-tag had no major effect on the oxidation kinetics compared to His_6_-Tsa1 (Supplementary Table 3).

For the yeast Trx1-dependent reduction, three phases were observed for His_6_-Tsa1(S_2_) and two phases for His_6_-Tsa2(S_2_) (Fig. 4a,d). The second-order rate constant *k*_1red_ of 3.9 × 10^6^ M^−1 ^s^−1^ for the formation of the mixed disulfide between His_6_-Tsa2 and yeast Trx1 was slightly higher than that for the mixed disulfide of His_6_-Tsa1 (Fig. 4e and Supplementary Table 3). Similar *y*-axis intercepts, 8.7 s^−1^ for His_6_-Tsa1 and 10.8 s^−1^ for His_6_-Tsa2, suggest either a reverse reaction or a conformational change. At higher Trx1 concentrations, the second and third phases exhibited pseudo-first-order reaction kinetics, with *k*_2red_ values around 10 s^−1^ for both enzymes (Fig. 4e and Supplementary Table 3). The second phase likely corresponds to the formation of reduced peroxiredoxins and Trx1(S_2_). The third phase with *k*_3red_ (0.6 s^−1^) for His_6_-Tsa1 may indicate a Trx1-induced decamer dissociation, a process not observed for His_6_-Tsa2.

The oxidation kinetics of copurified recombinant Strep-Tsa1–His_6_-Tsa2 hetero-oligomers were broadly similar to those of the individual enzymes (Fig. 4f) but characterization of the first oxidation phase required a quadruple exponential fit. The rate constants, *k*_1oxA_ and *k*_1oxB_, had intermediate values of 6.1 × 10^7^ M^−1^ s^−1^ and 2.8 × 10^7^ M^−1^ s^−1^ compared to the *k*_1ox_ values of the individual enzymes (Fig. 4f and Supplementary Table 3). The second and third phases displayed similar kinetics to those observed for the homo-oligomeric enzymes, yielding *k*_2ox_ and *k*_3ox_ values of 45 and 3.6 s^−1^, respectively. Premixed 1:1 mixtures of recombinant His_6_-Tsa1 and His_6_-Tsa2, which do not exchange subunits and thus do not assemble into hetero-oligomers, showed similar oxidation kinetics as the individual enzymes, with *k*_1oxA_ and *k*_1oxB_ values from a double exponential fit of 9.8 × 10^7^ M^−1^ s^−1^ and 1.2 × 10^7^ M^−1^ s^−1^ (Supplementary Table 3). Thus, the oxidation kinetics of Strep-Tsa1 and His_6_-Tsa2 mixtures reflect the superposition of the individual enzyme activities, whereas Strep-Tsa1–His_6_-Tsa2 hetero-oligomers exhibit intermediate macroscopic rate constants (*k*_1ox_) for the reaction with H_2_O_2_.

The reduction kinetics of Strep-Tsa1–His_6_-Tsa2 hetero-oligomers resembled those of His_6_-Tsa1 but required a quadruple exponential fit with two preset rate constants (Fig. 4g). The best fit was obtained using *k*_obs_ values for the first reduction phase from the individually purified enzymes at the corresponding Trx1 concentration. The remaining *k*_obs_ values were consistent with those observed for His_6_-Tsa1. This suggests that the reduction kinetics of Strep-Tsa1–His_6_-Tsa2 hetero-oligomers can be approximated by superimposing the reduction profiles of the individually purified enzymes.

**Extended Data Fig. 3:**
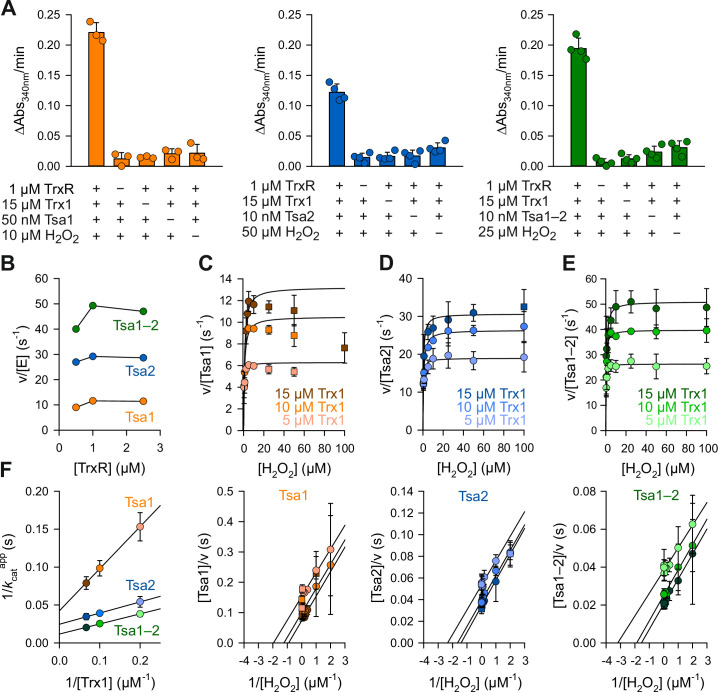
Coupled steady-state peroxidase assays for His_6_-Tsa1, His_6_-Tsa2, or copurified Strep-Tsa1 and His_6_-Tsa2. **a**, Controls to estimate the component-dependent background-consumption of NADPH. **b**, Control to exclude that the TrxR detection system was rate-limiting. **c**‒**e**, Direct Michaelis–Menten (top) and linearized Lineweaver–Burk (bottom) plots at variable H_2_O_2_ concentrations for 50 nM Tsa1, 10 nM Tsa2, or 10 nM copurified Tsa1 and Tsa2, respectively. Squares were omitted for regression analysis. All data were obtained from three (Tsa1) or four (Tsa2 and copurified Tsa1 and Tsa2) independent biological protein purifications and measurements. **f**, Secondary plot for the Trx1-dependent rate constants from **c**‒**e**. The *k*_cat_^app^ values for the oxidation by H_2_O_2_ and the rate constants for the Trx1-dependent reduction of oxidized Tsa1, Tsa2, or copurified Tsa1 and Tsa2 are listed in Supplementary Tables 4 and 5 and were estimated from the slopes in **c**‒**e** and **f**, respectively. Source data

To examine potential concentration-dependent effects on the rate constants, we also established a coupled steady-state peroxidase assay containing NADPH, TrxR, Trx1, H_2_O_2_ and nanomolar peroxidase concentrations (Extended Data Fig. 3a,b). The assays displayed a high degree of variability and required exact mixing routines as well as fixed volumes and dilutions of stock solutions, consistent with sensitive temperature-dependent and concentration-dependent effects on the oligomeric state. Variability was highest for His_6_-Tsa1, consistent with the results shown in Fig. 2. However, in contrast to previous reports that suggested rate constants around 10^4 ^M^−1^s^−1^ based on apparent *k*_cat_ and *K*_*m*_ values for recombinant Tsa1 and Tsa2 (ref. ^27^), our steady-state assays gave approximate rate oxidation constants in the range of 10^7^ – 10^8^ M^−1^ s^−1^ (Extended Data Fig. 3c–e and Supplementary Table 4), differing from the stopped-flow values by less than one order of magnitude (Supplementary Table 3). Approximate rate constants for the Trx1-dependent reduction were around 10^6^‒10^7^ M^−1^s^−1^ and also agreed within one order of magnitude (Extended Data Fig. 3f and Supplementary Tables 3 and 5).

One notable difference between the second-order rate constants from the stopped-flow and steady-state measurements was observed for His_6_-Tsa1, which showed a tenfold lower *k*_cat_^app^/*K*_*m*_^app^ value in the steady-state assays (Supplementary Tables 3 and 4). A likely explanation is the higher sensitivity of the threonine-containing active site of Tsa1 to hyperoxidation compared to the serine-containing active site of Tsa2 (ref. ^40^). The inactivation became relevant and detectable at micromolar-to-nanomolar peroxide-to-Tsa1 ratios but was reduced or even absent for Strep-Tsa1–His_6_-Tsa2 hetero-oligomers (Extended Data Fig. 3c,e).

Another notable difference between the stopped-flow and steady-state measurements was that the *k*_3ox_ or *k*_2red_ in Supplementary Table 3 were smaller than the *v*/(*E*) values shown in Extended Data Fig. 3b–f. One explanation is that nanomolar enzyme concentrations in the steady-state assay favor substantial populations of homodimeric or heterodimeric species with distinct kinetic properties compared with the predominantly decameric species present at micromolar concentrations in the stopped-flow measurements. Different dimer concentrations also explain the *v*/(*E*) values for 10 nM Strep-Tsa1–His_6_-Tsa2 hetero-oligomers, which were about four times higher than for 50 nM His_6_-Tsa1 and also 1.5 times higher than for 10 nM His_6_-Tsa2 (Extended Data Fig. 3c–e). Overall, His_6_-Tsa1, His_6_-Tsa2 and Strep-Tsa1–His_6_-Tsa2 hetero-oligomers exhibit similar second-order rate constants for H_2_O_2_ and Trx1 at both micromolar and nanomolar peroxiredoxin concentrations. However, nanomolar enzyme concentrations resulted in higher v/(E) values, likely reflecting an increased dimer abundance. In the steady-state assays, very high peroxide-to-enzyme ratios caused hyperoxidation of His_6_-Tsa1 but not of His_6_-Tsa2 or Strep-Tsa1–His_6_-Tsa2 hetero-oligomers.

In summary, analyses of oxidation and reduction kinetics of homo-oligomers, mixtures of Tsa1 and Tsa2 homo-oligomers, and Tsa1–Tsa2 hetero-oligomers revealed that the hetero-oligomers exhibit enzyme kinetics largely comparable to those of their homo-oligomeric counterparts. Although the formation of the sulfenic acid species in His_6_-Tsa1 was approximately tenfold faster than in His_6_-Tsa2, copurified Tsa1–Tsa2 hetero-oligomers displayed intermediate macroscopic rate constants for the reaction with H_2_O_2_ in the stopped-flow experiments. Our findings suggest that hetero-oligomerization and/or heterodimerization might protect Tsa1 from hyperoxidation at low enzyme concentrations at the cost of a slightly reduced reactivity with H_2_O_2_ at high enzyme concentrations.

### Tsa1–Tsa2 hetero-oligomers are enzymatically active in yeast

We previously demonstrated that a roGFP2-Tsa2ΔC_R_ probe can form enzymatically active hetero-oligomers with endogenous Tsa1 in the yeast cytosol^29^. To further investigate the assembly and enzymatic activity of Tsa1–Tsa2 hetero-oligomers in yeast, we expressed roGFP2-Tsa1 variants in Δ*tsa1*Δ*tsa2* yeast cells together with either wild-type (WT) or catalytically inactive Tsa2. Specifically, cells were transformed with plasmids encoding roGFP2-Tsa1ΔC_P_ΔC_R_, in which both catalytic cysteine residues were mutated to serine, and either Tsa2wt or Tsa2ΔC_P_ΔC_R_. Cells were then exposed to increasing concentrations of H_2_O_2_ (0–500 µM), and roGFP2 oxidation was quantified using a fluorescence plate-reader assay (Supplementary Fig. 12). In this assay, roGFP2 oxidation in response to H_2_O_2_ is efficiently catalyzed in *trans*, that is, by a partner peroxiredoxin in the dimer or decamer. Notably, in *trans* oxidation is only possible if the two peroxiredoxins are physically associated in a complex.

In Δ*tsa1*Δ*tsa2* cells coexpressing *roGFP2-TSA1ΔC*_*P*_*ΔC*_*R*_ and *TSA2wt*, roGFP2 oxidation was detected at exogenous H_2_O_2_ concentrations as low as 10 µM, indicating a highly sensitive response. In contrast, no roGFP2 oxidation was observed in cells coexpressing *roGFP2-TSA1ΔC*_*P*_*ΔC*_*R*_ and *TSA2ΔC*_*P*_*ΔC*_*R*_, even at the highest H_2_O_2_ concentration. Because roGFP2 is only very poorly oxidized directly by H_2_O_2_, these results confirm that Tsa1 and Tsa2 assembles into functional hetero-oligomers in the yeast cytosol^29,30,41,42^. In a complementary experiment, *roGFP2-TSA2ΔC*_*P*_*ΔC*_*R*_ was coexpressed with either *TSA1wt* or *TSA1ΔC*_*P*_*ΔC*_*R*_, resulting in robust roGFP2 oxidation only in the presence of Tsa1wt, with almost no oxidation with Tsa1ΔC_P_ΔC_R_ (Supplementary Fig. 12).

In summary, our findings confirm that Tsa1 and Tsa2 can form catalytically active hetero-oligomers in the yeast cytosol and function cooperatively to mediate roGFP2 oxidation.

### Native PRDX1 and PRDX2 form hetero-oligomers in HEK293 cells

**Fig. 5:**
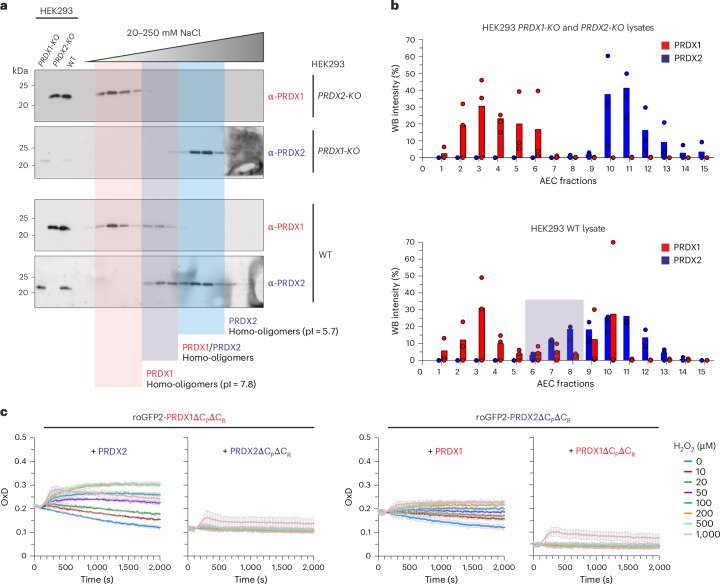
Native human PRDX1 and PRDX2 form hetero-oligomers in HEK293 cells. **a**,**b**, WB analysis (**a**) and quantification of AEC fractions (**b**) from HEK293 WT and *PRDX1-KO* and *PRDX2-KO* cell lysates (*n* = 3 experimental repeats, the 2 further experimental repeats are shown in Supplementary Fig. 13). Proteins from the three cell lines were separated by AEC and fractions subsequently analyzed by WB for the presence of PRDX1 and PRDX2. Lysates from the three cell lines were prepared and fractionated independently, with no mixing of lysates at any stage. WBs (**a**) and quantification (**b**) show that in the *PRDX-KO* cell lines, the PRDX1 and PRDX2 signals show no overlap in the middle fractions, whereas in the WT lysate, there is a clear overlap in the middle fractions, indicating a shift in the isoelectric point of the protein complex species, suggesting the presence of hetero-oligomers. **c**, Graphs showing change in OxD of the indicated roGFP2-PRDX fusion constructs in Δ*tsa1*Δ*tsa2* yeast cells treated with the indicated concentrations of H_2_O_2_. Experiments were repeated thrice with independent yeast cultures. Data are presented as mean ± s.d. AEC, anion exchange chromatography; pl, isoelectric point; OxD, degree of oxidation. Source data

To determine whether peroxiredoxin hetero-oligomerization extends beyond yeast, we investigated human PRDX1 and PRDX2, which were recently shown to form hetero-oligomers in vitro^31^. Despite sharing 78% sequence identity, PRDX1 and PRDX2 have distinct isoelectric points (7.80 and 5.71, respectively), allowing to use ion-exchange chromatography to separate them in Flp-In T-Rex HEK293 cell lysates (Fig. 5 and Supplementary Fig. 13). Western blot (WB) analysis of collected fractions revealed that, although PRDX1 and PRDX2 primarily eluted at different positions along the NaCl gradient, they also eluted across a broad range of partially overlapping fractions (Fig. 5a,b).

To further explore this interaction, we repeated the experiment using lysates from CRISPR–Cas9 knockout (KO) cells lacking either *PRDX1* or *PRDX2* (ref. ^43^). In *PRDX1 KO* and *PRDX2 KO* cells, the remaining PRDX isoform eluted in a narrower range of fractions, with no overlap observed (Fig. 5a,b). This shift in elution pattern suggests that PRDX1 and PRDX2 influence each other’s chromatographic behavior and provides strong evidence for their interaction as native hetero-oligomers.

To assess whether PRDX1–PRDX2 hetero-oligomers are enzymatically active, we used a yeast-based roGFP2 assay. Heterologous *roGFP2-PRDX1ΔC*_*P*_*ΔC*_*R*_ was expressed in Δ*tsa1*Δ*tsa2* yeast cells together with either *PRDX2* or *PRDX2ΔC*_*P*_*ΔC*_*R*_ and roGFP2 oxidation was measured upon H_2_O_2_ exposure (Fig. 5c). Consistent with our observation with yeast Tsa1 and Tsa2, roGFP2 oxidation was observed only when PRDX2 was present, indicating that PRDX1 requires an active PRDX2 partner to mediate roGFP oxidation. Likewise, roGFP2-PRDX2ΔC_P_ΔC_R_ only supported roGFP2 oxidation in the presence of PRDX1, but not PRDX1ΔC_P_ΔC_R_ (Fig. 5c).

In summary, these findings demonstrate that PRDX1 and PRDX2 form enzymatically active hetero-oligomers in both HEK293T cells and yeast and support the presence of peroxiredoxin hetero-oligomerization across species.

### Diverse eukaryotic peroxiredoxins form hetero-oligomers

**Fig. 6:**
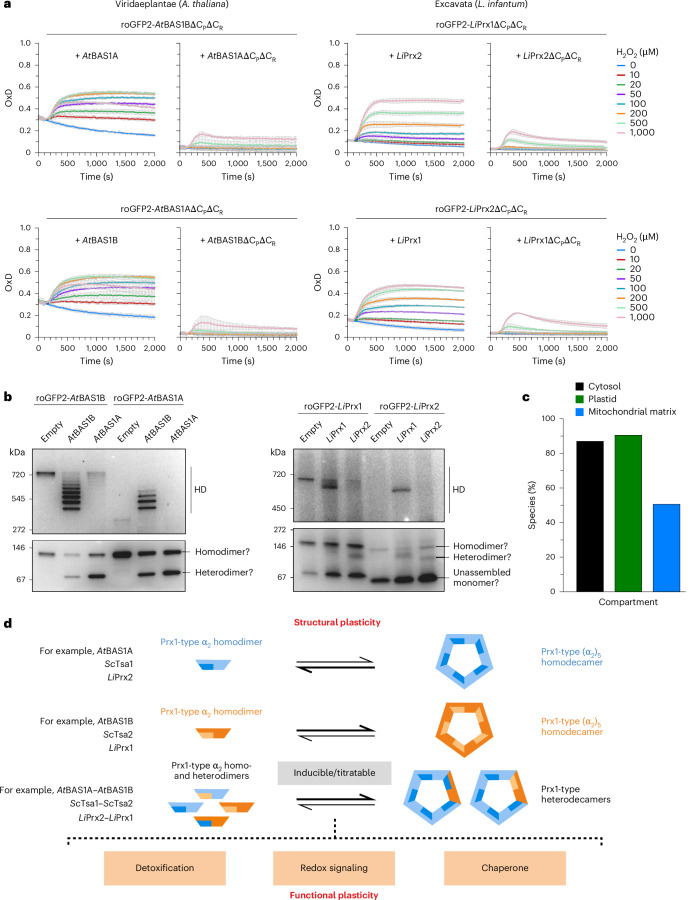
Hetero-oligomerization is a common feature of eukaryotic peroxiredoxins. **a**, Graphs showing the change in the OxD in response to 1 mM H_2_O_2_ of roGFP2-*At*BAS1BΔC_P_ΔC_R_ and roGFP2-*At*BAS1AΔC_P_ΔC_R_ constructs (left) or roGFP2-*Li*Prx1ΔC_P_ΔC_R_ and roGFP2-*Li*Prx2ΔC_P_ΔC_R_ constructs (right) expressed in Δ*tsa1*Δ*tsa2* yeast together with either a WT or cysteine-less (ΔC_P_ΔC_R_) variant of the corresponding partner peroxiredoxin. Experiments were repeated thrice with independent yeast cultures. Data are presented as mean ± s.d. **b**, Clear native PAGE analysis of lysates from Δ*tsa1*Δ*tsa2* yeast strains expressing the indicated combination of constructs. Gels were imaged for GFP fluorescence. Two further experimental repeats are shown in Supplementary Fig. 14. **c**, Graph showing the percentage of sequenced eukaryotic genomes in which there are predicted to be two or more Prx1/AhpC-type peroxiredoxins present in the indicated subcellular compartments. **d**, Model illustrating the impact of peroxiredoxin hetero-oligomerization on oligomeric state. Substoichiometric subunit incorporation is capable of strongly shifting the dimer–decamer equilibrium. HD, homodecamer and heterodecamer. Source data

To further assess whether hetero-oligomerization is a conserved feature of peroxiredoxins across eukaryotes, we extended our analysis to peroxiredoxins from non-opisthokont species using the yeast-based roGFP2 system. We generated roGFP2 fusion constructs with LiPRX1 and LiPRX2 from the cytosol of the kinetoplastid parasite *L. infantum*^23^, as well as *At*BAS1A and *At*BAS1B from the chloroplast stroma of the plant *A. thaliana*^44,45^. Each construct was expressed in Δ*tsa1*Δ*tsa2* yeast cells together with either the WT or double cysteine mutant of the corresponding partner peroxiredoxin isoform (Fig. 6a). In all cases, roGFP2 oxidation was observed exclusively in the presence of the WT partner and not with the cysteine mutant, consistent with our observations for yeast and human peroxiredoxins.

To explore the oligomeric state of these hetero-oligomers, we coexpressed *roGFP2*-*AtBAS1A* and *roGFP2-AtBAS1B* with either an empty vector, *AtBAS1A* or *AtBAS1B* (Fig. 6b and Supplementary Fig. 14). While roGFP2-*At*BAS1B predominantly formed a decamer, roGFP2-*At*BAS1A was almost exclusively dimeric. Coexpression of *AtBAS1A* with *roGFP2-AtBAS1B* induced a shift from a decamer to a heterodimeric form, whereas coexpression of *AtBAS1B* with *roGFP2-AtBAS1B* maintained decamer stability, producing a series of heterodecamers with varying stoichiometries and a minor heterodimer band. Conversely, coexpression of *AtBAS1B* with *roGFP2*-*AtBAS1A* led to partial decamer formation, but only when *At*BAS1A subunits were a minority, indicating that AtBAS1A destabilizes decamers. Coexpression of *roGFP2*-*AtBAS1A* and *AtBAS1A* resulted exclusively in heterodimers.

Similar results were obtained for *L. infantum* peroxiredoxins (Fig. 6b). In yeast lysates, roGFP2-*Li*Prx1 primarily assembled into decamers, whereas roGFP2-*Li*Prx2 showed little to no oligomerization. Coexpression of *LiPRX2* with *roGFP2-LiPRX1* disrupted decamer formation and promoted a shift toward putative heterodimeric species, whereas coexpression with *LiPRX1* had no effect on decamer stability. Consistent with the apparent differences in decamer stability, coexpression of *roGFP2-LiPRX2* with *LiPRX1* induced a shift to decameric species, while coexpression with *LiPRX2* did not.

In summary, our experiments suggest that strong differences in dimer–decamer equilibria are widespread among eukaryotic peroxiredoxins. Even limited incorporation of a second peroxiredoxin isoform can markedly shift the prevailing oligomeric state, highlighting the functional significance of hetero-oligomerization across diverse organisms.

### Pairs of Prx1-type peroxiredoxins occur in most eukaryotes

Finally, we sought to determine the percentage of eukaryotes in which two or more Prx1/AhpC-type peroxiredoxin proteoforms are predicted to be present in the same subcellular compartment. An extensive sequence search identified 11,415 peroxiredoxin candidate sequences across 1,525 eukaryotic species representing the kingdoms Viridiplantae, Metazoa and Fungi. Of these species, 1,471 were predicted to contain at least one Prx1-type peroxiredoxin in the cytosol, and two or more Prx1-type cytosolic peroxiredoxins were predicted in 1,326 species (Fig. 6c and Supplementary Fig. 15). Mitochondria-targeted Prx1-type peroxiredoxins were predicted in 1,236 species, with 771 species predicted to contain two or more mitochondrial peroxiredoxins. Within the kingdom Viridiplantae, plastid-localized Prx1-type peroxiredoxins were predicted in 347 species, with two or more plastid-localized peroxiredoxins in 314 species.

Overall, our data indicate that peroxiredoxin hetero-oligomerization is likely to be relevant in more than 80% of sequenced eukaryotic species and probably occurs in several different subcellular compartments.

## Discussion

Peroxiredoxins have been intensively studied since their discovery more than 30 years ago^1,3,5,7,46,47^. Nonetheless, Prx1/AhpC-type peroxiredoxins have until now been examined almost exclusively as homo-oligomeric complexes. Here we show that hetero-oligomerization is a common feature of Prx1/AhpC-type peroxiredoxins throughout the domain Eukaryota. Our findings therefore call for a careful re-evaluation of peroxiredoxin structural dynamics and functional diversity (Fig. 6d).

Regulation of the oligomeric state of peroxiredoxins appears to be closely linked to their functions. For example, the decameric state of Prx1/AhpC-type peroxiredoxins has been associated with protein chaperone activity^48–52^, whereas both dimers and decamers retain enzymatic activity^6,11,53^. In a possible example of functional specialization through structural adaptation, differences in dimer–decamer equilibria are common among Prx1/AhpC-type peroxiredoxins within an organism. For example, human PRDX1 is more stable as a decamer than PRDX2 (refs. ^10,24^). Likewise, yeast Tsa2 is more stable in the decameric form than Tsa1 (ref. ^27^). We observe similar differences in the dimer–decamer equilibria of *A. thaliana* chloroplast and *L. infantum* cytosolic peroxiredoxins. However, this simplistic binary view of peroxiredoxins as either ‘type-A’ or ‘type-B’ becomes difficult to sustain in light of hetero-oligomerization. In the absence of mechanisms to actively prevent hetero-oligomerization, of which we have found no evidence, cells are likely to contain a complex mixture of peroxiredoxin hetero-oligomers with diverse subunit stoichiometries, potentially each having its own dimer–decamer equilibrium (Fig. 6d). On the other hand, the observation that even substoichiometric incorporation of Tsa2 can impart Tsa2-like properties onto a Tsa1 decamer may reduce the complexity somewhat, at least in yeast. Finally, the possible role of hetero-oligomerization in the formation of higher-order assemblies, including stacked decamers and 12-decamer-containing dodecahedrons, remains largely unexplored, and the biological functions of these structures are still unclear^13,54^.

Hyperoxidation has been shown to be an important factor leading to decamer stabilization in several peroxiredoxins. However, the hyperoxidation sensitivity of Tsa1 is found to be approximately ~9× and ~100× lower than human PRDX1 and PRDX2, respectively^39,40^. Consistent with these findings and the lack of a strong signal for hyperoxidation in our WBs, we consider it unlikely that hyperoxidation is substantially affecting the stability of the hetero-oligomers formed in our yeast induction assays. However, given that changes in just one or two subunits can influence the properties of the entire decamer, hyperoxidation may still become relevant under other experimental conditions. For example, cell growth stage, carbon source and cell density are all important factors influencing hyperoxidation sensitivity in our hands.

Hetero-oligomerization not only influences oligomer stability but also brings together isoform-specific biochemical properties, including differences in isoelectric point, catalytic rates and susceptibility to hyperoxidation^10,24,27^, as well as modifications including limited proteolysis^55^, tyrosine nitration^56^, S-nitrosylation^57^, S-glutathionylation^14^ and phosphorylation^58^. By allowing for a graduated blending of properties, peroxiredoxin hetero-oligomerization may broaden functional outputs, for example, by modulating target protein specificity in redox signaling relays or altering the spectrum of potential chaperone clients. The structural plasticity of peroxiredoxins may therefore underpin functional plasticity, positioning them as potential hubs for integrating and transmitting signals within specific signaling pathways (Fig. 6d). This conceptual shift also raises new questions. If cells predominantly contain diverse hetero-oligomeric assemblies instead of pure homo-oligomeric complexes, how do individual isoforms maintain selective interactions with specific target proteins such as STAT3 (refs. ^58–60^) or ASK1 (ref. ^61^)? How signaling fidelity is preserved in the context of hetero-oligomeric mixtures remains unknown and should now be explored.

The extent to which cells can regulate the formation and subunit composition of hetero-oligomers remains unclear. One obvious point of control is transcription. As shown here, this mechanism is prominent in yeast—under oxidative challenge, *TSA2*, normally expressed at much lower levels than *TSA1*, is strongly induced, leading to hetero-oligomers with a much broader range of subunit stoichiometries than those found in unstressed cells. Post-translational modifications may also modulate hetero-oligomer composition by altering interactions between different peroxiredoxin isoforms. Whether subunits can dynamically exchange between oligomers is unclear and may differ among isoforms depending on the stability of their dimeric and decameric states.

Are hetero-oligomers unique to Prx1-type enzymes? In principle, Prx1-type enzymes share a higher degree of structural similarity with Prx6-type enzymes in contrast to other peroxiredoxin subfamilies^8^. To explore this possibility, we recently analyzed the cytosolic Prx1-type and Prx6-type enzymes from the malaria parasite *Plasmodium falciparum* but found no evidence of interaction^62^. In summary, while further studies are needed to investigate potential interactions among other peroxiredoxin subfamilies, current evidence suggests that heterodimer and hetero-oligomer formation is likely restricted to members of the Prx1/AhpC-type subfamily.

Future work should clarify how hetero-oligomerization influences peroxiredoxin-mediated signaling and whether it contributes to diseases where redox homeostasis is disrupted. Given the increasing recognition of peroxiredoxins in aging, cancer and neurodegenerative diseases, understanding how hetero-oligomerization influences their function may reveal new principles of redox regulation and uncover therapeutic opportunities. Defining the structural basis of hetero-oligomerization will be essential: cryo-EM and crystallography can resolve the interaction interfaces governing stability, while targeted mutagenesis can identify critical residues that drive hetero-oligomer assembly and link these to functional consequences.

In summary, peroxiredoxin hetero-oligomerization remains a largely underexplored phenomenon that likely holds broad significance for cellular function and disease.

## Methods

### Materials

*N*,*N*,*N*′,*N*′-tetramethylazodicarboxamide (diamide), DTT, ethylenediaminetetraacetic acid (EDTA) and *N*-ethylmaleimide (NEM) were purchased from Sigma-Aldrich; diethylenetriaminepentaacetic acid (DTPA) was from Carl Roth; isopropyl-β-D-1-thiogalactopyranoside was from Serva; H_2_O_2_ was from VWR and desthiobiotin was from IBA Lifesciences. PCR primers were purchased from Metabion. All restriction enzymes, DNA polymerase and T4 DNA ligase were purchased from New England Biolabs.

### Cloning and mutagenesis

The detailed information on the primers/constructs is presented in Supplementary Table 6.

*TSA1* was PCR-amplified (Phusion HF) from pET15b/*His-TSA1* and subcloned into pET45b/Strep (KpnI/AvrII), then into pCOLADuet MCS2 (NdeI/XhoI). *TSA2* from pET15b/*His*_*6*_-*TSA2* was inserted into pCOLADuet MCS1 (NcoI/BamHI) to yield pCOLADuet/*His*_*6*_*-TSA2/Strep-TSA1*. *TSA1ΔCR* (resolving Cys mutant) was generated by site-directed mutagenesis and cloned analogously. An EPEA tag was introduced into *TSA2* to generate pCOLADuet/*His*_*6*_*-TSA2*-*EPEA/Strep-TSA1* and pET15b/His_6_-TSA2-EPEA. All plasmids were verified by Sanger sequencing.

### Protein expression and purification

Homo-oligomers *Strep-TSA1* (pET45b plasmid), *His*_*6*_*-TSA1* (pET15b plasmid), *His*_*6*_*-TSA2* (pET15b plasmid) and *His*_*6*_*-TSA2-EPEA* (pET15b plasmid), as well as hetero-oligomers *Strep-TSA1–His*_*6*_*-TSA2* (pCOLADuet-1 plasmid) and *Strep-TSA1–His*_*6*_*-Tsa2-EPEA* (pCOLADuet-1 plasmid), were expressed in *E. coli* strain SHuffle T7 Express in Luria Broth (LB) and induced with 0.5 mM IPTG. Eluted proteins were buffer-exchanged, assessed by SDS–PAGE, Clear/Blue Native gel and WB, and quantified at A_280_. Extinction coefficients: Strep-Tsa1 29,575 M^−1^ cm^−1^, His_6_-Tsa2-EPEA 24,075 M^−1^ cm^−1^, hetero-oligomer 26,825 M^−1^ cm^−1^, Nbsyn220 27,180 M^−1^ cm^−1^ and reduced Trx 9,970 M^−1^ cm^−1^—were calculated in the EXPASY webserver based on the sequence.

#### Tsa1 and Tsa2-EPEA homo-oligomers

pET45b plasmid, containing *Strep-TSA1*, and pET15b, containing *His*_*6*_*-TSA2-EPEA*, were independently expressed in *E. coli* strain SHuffle T7 Express in LB (100 μg ml^−1^ ampicillin) at 30 °C. Cells were induced with 0.5 mM IPTG, grown overnight at 30 °C and collected by centrifugation. Cells were lysed in 100 mM HEPES/NaOH pH 7.9, 300 mM NaCl, 20 mM MgCl_2_, 1 μg ml^−1^ DNaseI, 50 μg ml^−1^ leupeptin and 0.1 mg ml^−1^ AEBSF by sonication at 4 °C, and centrifuged at 39,846*g* for 25 min at 4 °C.

For His_6_-Tsa2-EPEA, supernatant was incubated with previously equilibrated Ni^2+^-Sepharose High Performance beads for 1 h at 4 °C in equilibration buffer (100 mM HEPES/NaOH pH 7.9, and 300 mM NaCl). Proteins were eluted using a stepwise gradient of 100 mM HEPES/NaOH pH 7.9, 300 mM NaCl and 1 M imidazole. For Strep-Tsa1, supernatant was loaded onto a StrepTactin column (IBA Lifesciences) equilibrated with 100 mM Tris–HCl, pH 8, 150 mM NaCl and 1 mM EDTA, and then eluted with the same buffer with 1 mM desthiobiotin in a one-step gradient. Fractions containing His-Tsa2-EPEA or Strep-Tsa1 were dialyzed against 100 mM Tris–HCl, pH 8, 150 mM NaCl and 1 mM EDTA, and stored at −80 °C.

#### Tsa1–Tsa2-EPEA hetero-oligomer

*Strep-TSA1* and *His*_*6*_*-TSA2-EPEA* were expressed in pCOLADuet-1 plasmid and purified by tandem chromatography (Ni→Strep). Fractions from the Ni-NTA containing Strep-Tsa1 and His_6_-Tsa2-EPEA were dialyzed overnight at 4 °C against 100 mM Tris–HCl, pH 8, 150 mM NaCl and 1 mM EDTA with two buffer changes. The dialyzed sample was loaded onto a StrepTactin column. Fractions containing the hetero-oligomer were dialyzed against 100 mM Tris–HCl, pH 8, 150 mM NaCl and 1 mM EDTA, and stored at −80 °C.

#### Homo-oligomers and hetero-oligomers for kinetics

Cells were induced with 0.5 mM IPTG, grown for 4 h at 37 °C, and collected by centrifugation. Cell pellets were resuspended in 100 mM Na_*x*_H_*y*_PO_4_, pH 8.0, 20 mM imidazole, 300 mM NaCl at 4 °C and stirred on ice for 45 min with DNaseI and 10 mg l^−1^ lysozyme before sonication and clarified by centrifugation. Supernatants containing His-tagged Tsa1 or Tsa2 were loaded onto Ni-NTA agarose columns. The columns were eluted with 100 mM Na_*x*_H_*y*_PO_4_, pH 8.0, 200 mM imidazole and 300 mM NaCl at 4 °C. Supernatants containing Strep-tagged Tsa1 were loaded onto StrepTactin Superflow columns. The columns were eluted with 100 mM Tris–HCl, pH 8.0, 150 mM NaCl, 1 mM EDTA and 1 mM desthiobiotin at 4 °C. For the tandem purification using pCOLADuet constructs, supernatants were first purified by Ni-NTA and fractions were separately purified by StrepTactin as described above. For the reverse tandem purification, supernatants were purified by StrepTactin and fractions were then purified by Ni-NTA.

#### Thioredoxin and thioredoxin reductase for kinetics

His-ScTrx1 was produced in *E. coli* strain XL1-Blue at 37 °C in LB^38^. Cells were induced with 0.5 mM IPTG at an optical density of 0.5, grown for 4 h at 37 °C, collected by centrifugation and purified by Ni-NTA. His-ScTrr1 was produced in *E. coli* strain SHuffle T7 Express in LB containing 40 µM FAD. Cells were first grown at 30 °C until an optical density of 0.8, cooled in an ice-water bath, induced with 0.1 mM IPTG, grown overnight at 16 °C, collected by centrifugation and purified by Ni-NTA.

#### Nanobody (Nbsyn2.20)

His_6_-Nbsyn2.20 was expressed and purified as described^32^, with a final Superdex75 step (PBS pH 7.4). Purified protein was stored at −20 °C.

### Stopped-flow kinetics

Proteins were reduced with 5 mM DTT for 30 min on ice and desalted into assay buffer (100 mM Na_*x*_H_*y*_PO₄, pH 7.4, 0.1 mM DTPA at 25 °C). Oxidized enzymes were generated with equimolar H_2_O_2_ for 30 min on ice. Reactions were recorded at 25 °C on a thermostated SX-20 spectrofluorometer (Ex = 295 nm, slit width = 2 mm, total emission). For oxidation, 1 or 10 µM reduced Tsa1 and/or Tsa2 was mixed with H_2_O_2_. For reduction, 2 µM oxidized peroxiredoxins were mixed with reduced ScTrx1. Traces (≥3 technical replicates) were fit (double/triple exponential) in SigmaPlot 13.0 to obtain *k*_obs_; three biological replicates were fit (linear/hyperbolic) to derive rate constants.

### Steady-state kinetics

Coupled steady-state kinetic assays with recombinant His-tagged *Sc*Trr1, *Sc*Trx1, Tsa1, Tsa2 or copurified Strep-Tsa1–His-Tsa2 were carried out in assay buffer (100 mM Na_*x*_H_*y*_PO_4_, 0.1 mM DTPA, pH 7.4 at 25 °C) at 25 °C using a thermostated Jasco V-650 UV–visual spectrophotometer. The consumption of NADPH was monitored at 340 nm (*ε* = 6.22 mM^−1^ cm^−1^). Stock solutions of 4 mM NADPH, 0.98 mM H_2_O_2_ and all enzymes were freshly prepared in assay buffer before each experiment. The activity of *Sc*Trr1 in U ml^−1^ was determined with 100 μM NADPH and 20 µM ScTrx1. Briefly, NADPH and *Sc*Trr1 were mixed in assay buffer, a baseline was recorded for 30 s and the ScTrxR assay was started by the addition of ScTrx1. Peroxidase assays were optimized for ΔAbs/min values of 0.02‒0.2 and contained 150 μM NADPH, 1 µM *Sc*Trr1 (corresponding to 0.4 mU ml⁻^1^), 5, 10 or 15 µM ScTrx1, 0.5‒100 µM H_2_O_2_, and 50 nM Tsa1, 10 nM Tsa2 or 10 nM Strep-Tsa1–His-Tsa2. After a baseline was recorded for 30 s, peroxidase assays were started by the simultaneous addition of peroxide and peroxidase. For the determination of apparent *k*_cat_ and *K*_*m*_ values, initial activities were corrected by subtracting the final slope of the baseline using the Spectra Analysis program (Spectra Manager (v2), Jasco). Negative controls included the omission of each of the assay components. Controls with variable *Sc*Trr1 concentrations confirmed that the detection system was not rate-limiting at all substrate concentrations tested. Kinetic data from triplicate measurements from independent protein purifications were analyzed according to Michaelis–Menten theory and by linear regression according to Lineweaver–Burk, Eadie–Hofstee and Hanes theory in SigmaPlot (v11.0, Systat) to identify outliers and deviations from Michaelis–Menten kinetics.

### Western blotting

Samples (±reducing/nonreducing) were denatured in Laemmli buffer (95 °C, 5 min), separated by SDS–PAGE or clear native PAGE, transferred to PVDF, stained and probed with anti-His, anti-EPEA or anti-Strep, as well as secondary antibody. For tandem-purified His_6_-Tsa2/Strep-Tsa1ΔCR, 10 mM NEM (1 h, ice) prevented artificial disulfides. Band intensities were analyzed or quantified in ImageJ against calibrated standards.

### BLI

For the BLI assay on Octet R8 system (Sartorius), biotinylated ligands (Strep-Tsa1 and His_6_-Tsa2-EPEA, homo-oligomer and hetero-oligomer, positive-control anti-EPEA nanobody, negative-control BSA) were loaded on Streptavidin (SA) Biosensors (10 µg ml^−1^, 100 s, 25 °C). The concentration of Nbsyn2.20 (analyte) was fixed (50 nM) in 10 mM HEPES/NaOH pH 8, 137 mM NaCl and 2 mM KCl with 1% BSA/0.05% Tween 20. Association and dissociation phases were recorded (600 s each at 25 °C). Data were reference-subtracted, filtered and fit (1:1 local) to extract kinetics (*n* = 3).

### Nanodifferential scanning fluorimetry

Dialyzed proteins (5 µM) in 10 mM HEPES/NaOH pH 8, 137 mM NaCl and 2 mM KCl were heated to 100 °C (2 °C min^−1^). Fluorescence ratio was measured at 350/330 nm in a Prometheus spectrophotometer (NanoTemper) and the inflection point values gave *T*_*m*_ (*n* = 3).

### CD

Proteins were buffer-exchanged into 10 mM sodium phosphate pH 8, 140 mM NaF, diluted to 0.35–0.40 mg ml^−1^ and measured from 190 to 260 nm (1-mm path, 50 nm min^−1^, 1-nm bandwidth) in a BioLogic MOS-500 CD spectropolarimeter (BioLogic). Five spectra were averaged at temperatures selected from nanoDSF inflection points.

### Mass photometry

Dialyzed samples (5 µM) were incubated 15 min at 20 °C, 30 °C, 40 °C or 45 °C, diluted 25× into 10 mM HEPES/NaOH pH 8, 137 mM NaCl and 2 mM KCl, and measured on a Refeyn OneMP (6,000 frames, 60 s). Contrast-to-mass was calibrated (MassFerence P1, 88–344 kDa). Counts were binned and plotted versus mass; mean molecular weight ± s.d. were calculated from triplicates. Relative abundance (%) of LMW species and decameric oligomers was calculated.

### Negative-stain EM

Formvar/Carbon 400 Mesh, Cu grids were glow discharged at 4–5 mA and 0.3 mbar vacuum for 30 s. Three microliters of freshly diluted sample (0.02 mg ml^−1^) in 10 mM HEPES/NaOH pH 8, 137 mM NaCl and 2 mM KCl were incubated on the grids for 30 s, followed by staining with 2% uranyl-acetate. A total of 20 micrographs were collected on a JEOL 1400+ microscope, equipped with a LaB6 filament operating at 120 kV. Micrographs were recorded using a TVIPS F416 CCD camera using a nominal magnification of 60,000, corresponding to a magnified pixel size of 1.94 Å px^−1^ and a defocus range of 0 to −1.5 μm. The micrographs were processed using CryoSparc (v4.6.0). After running patch contrast transfer function estimation, particles were picked by blob-picker and extracted using a box size of 256 px. These particles were subjected to two-dimensional classification. Particle diameters were measured by TVIPS imaging software.

### Hetero-oligomer–nanobody complex

Strep-Tsa1/His_6_-Tsa2-EPEA (5 µM) and Nbsyn2.20 (6 µM) were incubated for 20 min at room temperature in 10 mM HEPES/NaOH pH 8, 137 mM NaCl and 2 mM KCl, and analyzed by mass photometry and negative-stain EM.

### MALDI-TOF MS

Samples were exchanged into 0.1% TFA; monomers were obtained by reduction with DTT (1:20 molar ratio) and rebuffering (TFA + 1 mM DTT). Furthermore, 1:1:1 mixture of protein sample, 2,5-dihydroxyacetophenone (DHAP) matrix (Bruker) and 2% vol/vol TFA were spotted in duplicate on an MTP ground steel plate. Spectra were acquired on an Ultraflextreme enhanced MALDI-TOF/TOF MS system (Bruker) in linear positive mode (range = 5,000–50,000 *m*/*z*) and processed using FlexAnalysis, with two replicates per sample. All acquisition methods were provided by the manufacturer and optimized and calibrated with an in-house calibration standard (15–122 kDa, five calibrants).

### LC–MS/MS

Samples were digested using S-Trap mini spin columns according to the manufacturer’s instructions (Protifi). Intact peptides were detected in the Orbitrap Fusion Lumos at a resolution of 120,000. Peptides were selected for MS/MS using HCD setting at 30, and ion fragments were detected in the IonTrap. A data-dependent procedure that alternated between 1 MS scan followed by MS/MS scans was applied for 3 s for ions above a threshold ion count of 1.0 × 10^4^ in the MS survey scan with 30.0 s dynamic exclusion. MS1 spectra were obtained with an automatic gain control target of 4 × 10^5^ ions and a maximum injection time set to auto, and MS2 spectra were acquired with an automatic gain control target of 1 × 10^4^ ions and a maximum injection set to auto. Oxidation of methionine was set as a variable modification, and the thiomethylation of cysteine was fixed. Trypsin specificity with semispecific cleavage was applied, allowing up to two missed cleavages.

### Yeast strains and induction

The detailed information on strains is presented in Supplementary Table 7. The *TSA1*::*ROGFP2-TSA1*, *TSA1*::*ROGFP2-TSA1* Δ*tsa2*, *TSA2*::*ROGFP2-TSA2* and *TSA2*::*ROGFP2-TSA2* Δ*tsa1* strains were generated by standard homologous recombination approaches^63^. *TSA1* and *TSA2* genes were first replaced with a URA3 cassette, selecting for uracil auxotrophy on Hartwell’s Complete (HC) agar plates lacking uracil. The URA3 cassette was replaced by *ROGFP2-TSA1* or *ROGFP2-TSA2* with selection on HC plates containing 0.1% wt/vol 5-fluoroorotic acid (5-FOA). Plates were incubated at 30 °C for 48 h. Colonies were picked and screened by PCR.

### Yeast peroxiredoxin hetero-oligomer induction

Yeast strains were grown at 30 °C in HC medium for 24 h, diluted to an *D*_600_ = 1 in fresh medium and grown for 1 h. Cultures were treated for the indicated time points with 1 mM H_2_O_2_. At these time points, 25 *D*_600_ units were collected by centrifugation at 800*g* for 3 min at room temperature. Cells were resuspended in 50 mM Tris–HCl, pH 7.7, 50 mM NaCl, 10% (vol/vol) glycerol, 20 mM NEM, 100 µM DTPA, 1× protease inhibitor cocktail and lysed by glass-bead homogenization. Lysates were cleared by centrifugation at 10,000*g* for 1 min at 5 °C. Protein concentration was determined by Bradford assays and 20 µg protein was loaded onto a 3–12% Clear-Native gel. Gels were imaged for GFP fluorescence. Cell lysates were also analyzed for total GFP fluorescence using a BMG Labtech CLARIOstar plate-reader.

### roGFP2 activity assays (yeast)

BY4742 Δ*tsa1*Δ*tsa2* cells were transformed with p415TEF and p416TEF plasmids for the expression of roGFP2-peroxiredoxin fusion constructs and unfused peroxiredoxin variants, respectively (Supplementary Table 7). Cells were grown to late-logarithmic phase (*D*_600_ = 3–4) in HC medium lacking the appropriate amino acids for plasmid selection. Cells were collected at room temperature and resuspended to a final concentration of 7.5 *D*_600_ U ml^−1^. Cells were transferred to a flat-bottomed 96-well imaging plate (BD Falcon, 353219), with 200 µl cell suspension per well. Fully oxidized and fully reduced controls were established by the addition of 20 mM diamide and 100 mM DTT, respectively^64^. Fluorescence was monitored in a BMG Labtech CLARIOstar plate-reader at 400 nm and 488 nm, and emission at 510 nm. The experiment was initiated by H_2_O_2_ at the indicated concentration. Degree of oxidation (OxD) roGFP2 was calculated according to equation (1).

1 $$\begin{array}{rcl}{{\rm{OxD}}}_{{\rm{roGFP}}2}=\displaystyle \frac{({I}_{400{\rm{sample}}}\times {I}_{480{\rm{red}}})-({I}_{400{\rm{red}}}\times {I}_{480{\rm{sample}}})}{\begin{array}{c}({I}_{400{\rm{sample}}}\times {I}_{480{\rm{red}}}-{I}_{400{\rm{sample}}}\times {I}_{480{\rm{ox}}})\\ +({I}_{400{\rm{ox}}}\times {I}_{480{\rm{sample}}}-{I}_{400{\rm{red}}}\times {I}_{480{\rm{sample}}})\end{array}}\end{array}$$

### Mammalian PRDX1/PRDX2 hetero-oligomers

HEK293 FLP-In/T-REx WT, PRDX1 KO and PRDX2 KO cells were generated previously in Jan Riemer’s lab^43^. Cell lysates (20 mM Tris–HCl, pH 9.2, 20 mM NaCl) were fractionated on a HiTrap Q column with a 0–250 mM NaCl gradient. Fractions (0.5 ml) were trichloroacetic acid-precipitated, washed with acetone, resolubilized in reducing Laemmli buffer and analyzed by SDS–PAGE/WB.

### Bioinformatics

Peroxiredoxin candidates were identified using iterative BLASTp (NCBI; v2.16.0) with BLOSUM45 on the nonredundant protein database (GenPept, Swiss-Prot, PIR, PDF, PDB, RefSeq; 9 Jan 2025), restricted to genomes at assembly level ‘chromosome’ or ‘complete’. New queries were generated from top-scoring hits in phylogenetic adjacent eukaryotic taxa to broaden and ensure coverage. Sequences containing characteristic peroxiredoxin PFAM domains (PF00578, PF08534, PF10417), identified through HMMER, were retained, and highly similar entries (>99% identity) within the same species were collapsed. Subcellular localization was predicted using DeepLoc 2.1 with default parameters.

### Reporting summary

Further information on research design is available in the Nature Portfolio Reporting Summary linked to this article.

## Online content

Any methods, additional references, Nature Portfolio reporting summaries, source data, extended data, supplementary information, acknowledgements, peer review information; details of author contributions and competing interests; and statements of data and code availability are available at 10.1038/s41589-026-02157-6.

## Supplementary information

Supplementary Information Supplementary Figs. 1–15, supporting data for Figs. 3, 10, 11, 13 and 14, Tables 1–7, and Note.

Reporting Summary

## Source data

Source Data Fig. 1 Unprocessed gels.

Source Data Fig. 2 Statistical source data.

Source Data Fig. 3 Unprocessed gels.

Source Data Fig. 3 Statistical source data.

Source Data Fig. 4 Statistical source data.

Source Data Fig. 5 Unprocessed gels.

Source Data Fig. 5 Statistical source data.

Source Data Fig. 6 Statistical source data.

Source Data Fig. 6 Unprocessed gels.

Source Data Extended Data Fig. 3 Statistical source data.

## Data Availability

All experimental data generated in this study are presented within the main text and Supplementary Information. Raw data relating to LC–MS experiments have been deposited in the PRIDE database, accession PXD060819, and can be accessed with the following login: reviewer_pxd060819@ebi.ac.uk and password: CK9xDcVBX9pU. Source data for all main and supplementary figures are provided. Source data are provided with this paper.
