# Supplementary material for: Hetero-oligomerization drives structural plasticity of eukaryotic peroxiredoxins
Source: Nat Chem Biol. 2026 Mar 10;22(4):580–92. doi: 10.1038/s41589-026-02157-6 (PMC13038412; doi:10.1038/s41589-026-02157-6)
Supplement: Supplementary file 1 — Supplementary Figs. 1–15, supporting data for Figs. 3, 10, 11, 13 and 14, Tables 1–7, and Note. [file 41589_2026_2157_MOESM1_ESM.pdf]

nature chemical biology

Supplementary information https://doi.org/10.1038/s41589-026-02157-6

Hetero-oligomerization drives structural plasticity of eukaryotic peroxiredoxins

In the format provided by the authors and unedited

Supplementary Information

Table of Contents

- **Supplementary Figures 1–15**
- **Source Data for Supplementary Figures**
- **Supplementary Tables 1–7**
- **Supplementary Note 1**
- **Supplementary References**

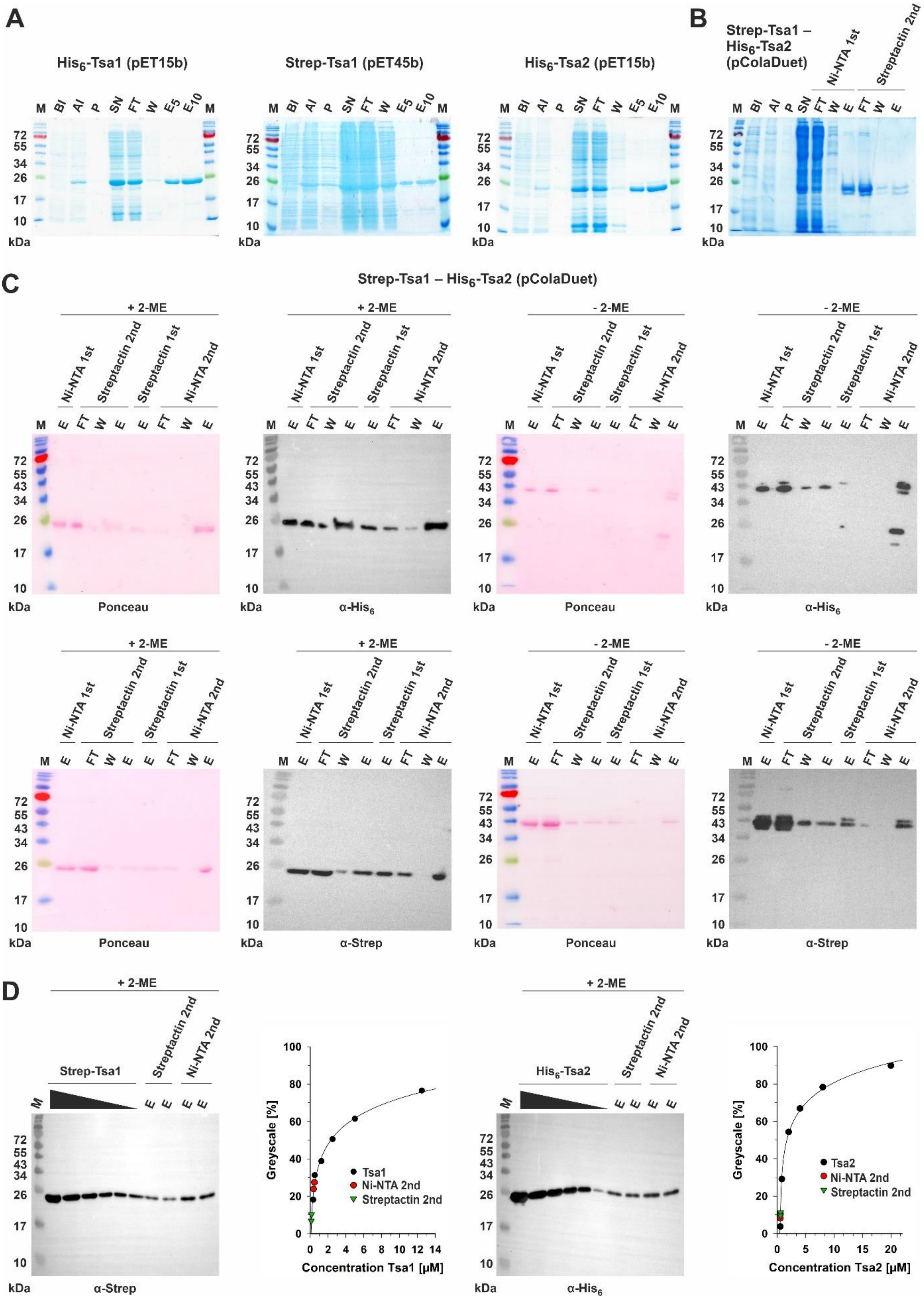

A
His6-Tsa1 (pET15b)
Strep-Tsa1 (pET45b)
His6-Tsa2 (pET15b)
M BI AI P SN FT W E5 E10 M
72
55
34
26
17
10
kDa
B
Strep-Tsa1 – His6-Tsa2 (pColaDuet)
Ni-NTA 1st
Streptactin 2nd
M BI AI P SN FT W E FT W E
C
Strep-Tsa1 – His6-Tsa2 (pColaDuet)
+ 2-ME
- 2-ME
Ni-NTA 1st
Streptactin 2nd
Streptactin 1st
Ni-NTA 2nd
M E FT W E E FT W E
72
55
43
34
26
17
10
kDa
Ponceau
α-His6
α-Strep
D
+ 2-ME
Strep-Tsa1
His6-Tsa2
Streptactin 2nd
Ni-NTA 2nd
M E E E E
Greyscale [%]
Concentration Tsa1 [µM]
Concentration Tsa2 [µM]
Tsa1
Tsa2
Ni-NTA 2nd
Streptactin 2nd
0 2 4 6 8 10 12 14
0 5 10 15 20
0 20 40 60 80 100

Supplementary Figure 1. Purification of recombinant Tsa1 and Tsa2 from *E. coli*.

A) SDS-PAGE analysis of representative individual purifications of recombinant His_6-Tsa1 (left), Strep-Tsa1 (middle), and His_6-Tsa2. **B**) SDS-PAGE analysis of a representative tandem affinity co-purification of recombinant Strep-Tsa1 and His_6-Tsa2. **C**) Western blot analysis of the tandem affinity co-purifications of recombinant Strep-Tsa1 and His_6-Tsa2. Samples were separated by reducing (left) or non-reducing (right) SDS-PAGE and blots were stained with ponceau as a loading control. The membranes were subsequently decorated with an antibody against either the His-tag (upper row) or the Strep-tag (lower row). Each membrane shows two tandem affinity co-purifications, one with Ni-NTA agarose followed by StrepTactin agarose and one with StrepTactin agarose followed by Ni-NTA agarose. **D**) Semi-quantitative western blot analysis of the protein content of co-purified Strep-Tsa1 and His_6-Tsa2. Known concentrations of individually purified Strep-Tsa1 or His_6-Tsa2 were used for calibration. Greyscales were quantified using ImageJ. The calculated molecular masses of His_6-Tsa1/2 and Strep-Tsa1 are 23.8 and 22.9 kDa, respectively. The calculated Tsa1:Tsa2 ratios for the "Streptactin 2nd" and "Ni-NTA 2nd" preparations were 0.23 and 0.75, respectively. M, marker; BI, before induction; AI, after induction; P, pellet after sonication; SN, supernatant; FT, flow-through; W, wash; $E_{(5,10)}$, eluate (5 or 10 µL loaded); 2-ME, 2-mercaptoethanol. All experiments were repeated three times with the same results.

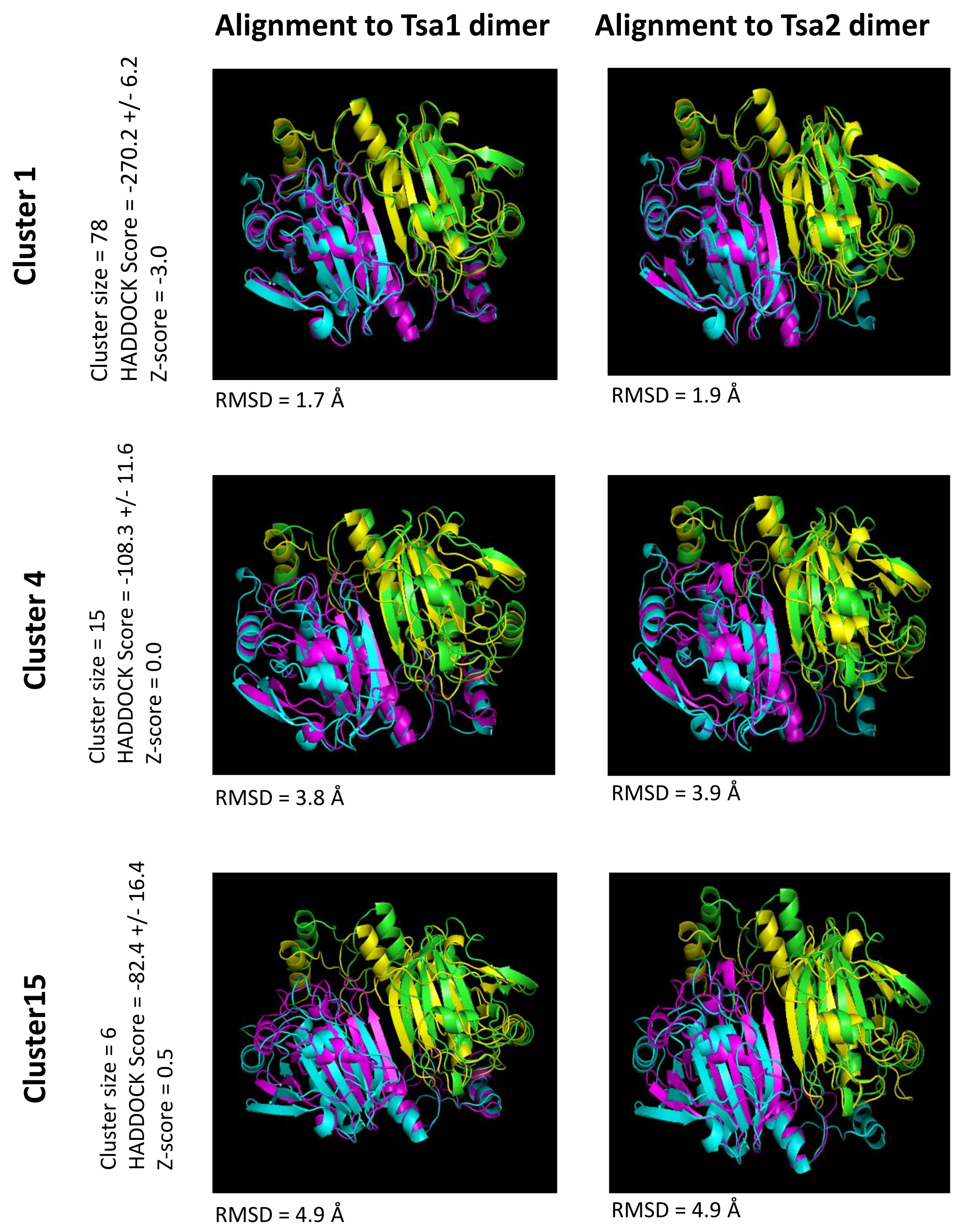

Supplementary Figure 2. HADDOCK-based assembly of Tsa1–Tsa2 B-type heterodimers

PDB files of chain A sequence from yeast TSA1 (3SBC) and chain A sequence from yeast TSA2 (5DVB) were loaded as molecule 1 and molecule 2 respectively. B-type interface residues of each chains were loaded as active residues (ß-sheet interchain interaction sites). HADDOCK clustered 281 structures into 23 clusters. Images of the top 3 clusters are shown with HADDOCK predicted Tsa1 (Blue) and Tsa2 (Green) compared to the crystal structure of Tsa1 (3SBC: left panels) or Tsa2 (right panels) respectively; in both cases, Chain A is depicted in yellow and chain B in magenta.

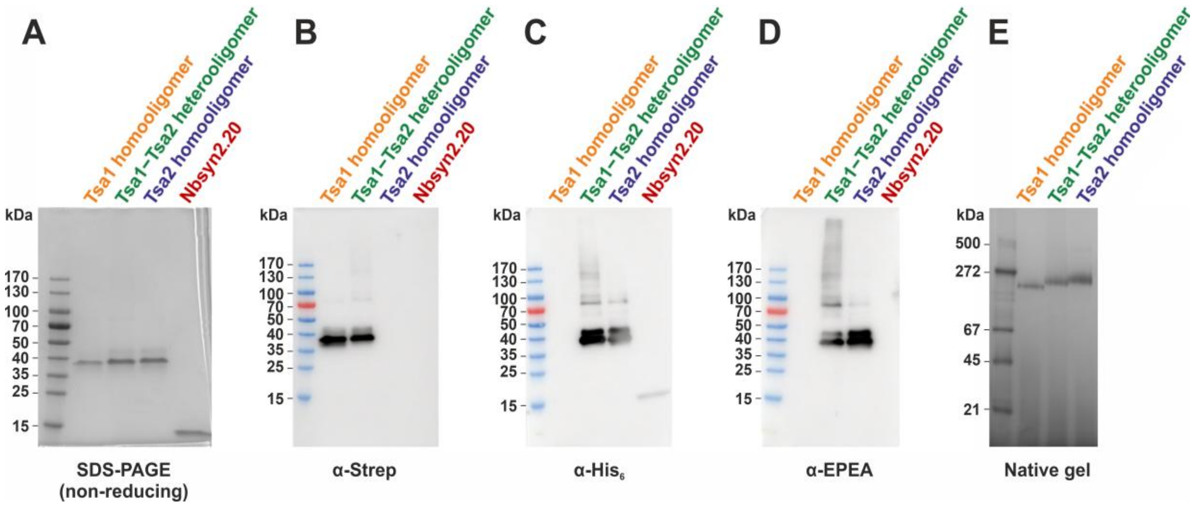

Supplementary Figure 3. Purified proteins present the correct tags identified by western blot

Purified Strep-Tsa1 homooligomer, His_6-Tsa2-EPEA homooligomer, 0.88 mol/mol Strep-Tsa1–His_6-Tsa2-EPEA heterooligomer and His-Nbsyn2.20 samples were separated on a 4–20% gradient SDS-PAGE gel and transferred onto PVDF membranes. The membranes were probed with anti-EPEA, HRP-conjugated anti-Strep and anti-His primary antibodies. Then, anti-EPEA and anti-His membranes were incubated with HRP-conjugated goat anti-mouse IgG secondary antibody. Protein bands were visualized with HRP substrate Pierce™ ECL western blotting substrate. **A)** 4–20% SDS-PAGE gradient gel **B)** anti-Strep blot **C)** anti-His_6 blot **D)** anti-EPEA blot **E)** Native gel analysis shows that Strep-Tsa1–His_6-Tsa2-EPEA heterooligomers form a single stable population in solution. 2 μg of protein samples (Strep-Tsa1 homooligomer, His_6-Tsa2-EPEA homooligomer, 0.88 mol/mol Strep-Tsa1–His_6-Tsa2-EPEA heterooligomer) were separated on a 4–16% precasted native gel (Serva). The protein ladder used was Native Marker Liquid Mix for BN/CN PAGE (Serva). All purifications were repeated three times with similar results.

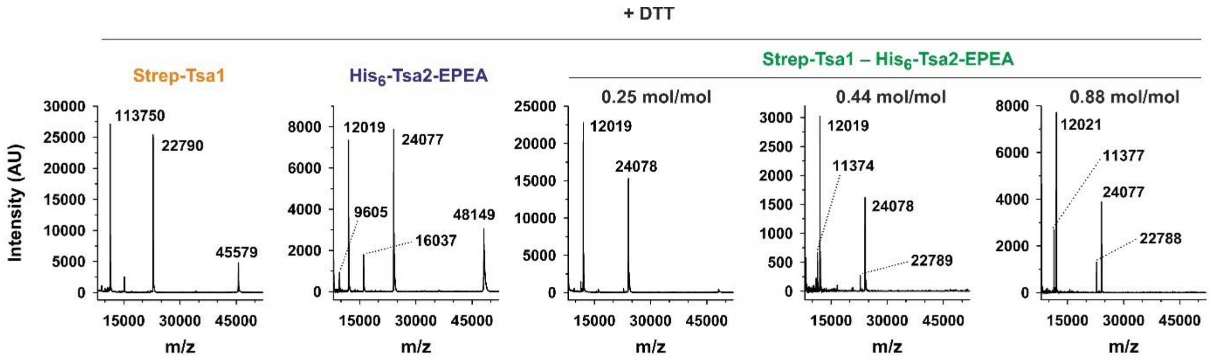

Supplementary Figure 4. Purified Strep-Tsa1 homooligomer, His_6-Tsa2-EPEA homooligomer, 0.88 mol/mol Strep-Tsa1–His_6-Tsa2-EPEA heterooligomer samples do not show any contamination with AhpC from *E. coli*

MALDI-TOF mass spectrometry analysis in reducing conditions reveals that Strep-Tsa1–His_6-Tsa2-EPEA heterooligomers are formed by Strep-Tsa1 and His_6-Tsa2-EPEA monomers in varying ratios. The ratio of each Strep-Tsa1–His_6-Tsa2-EPEA heterooligomer sample is displayed above the spectra. The intensity of the signal (AU) is shown at different m/z ratios. Duplicate measurements were performed for each sample, and data were analyzed by FlexAnalysis 3.4 (Bruker). The expected masses are displayed in **Supplementary Table 1.**

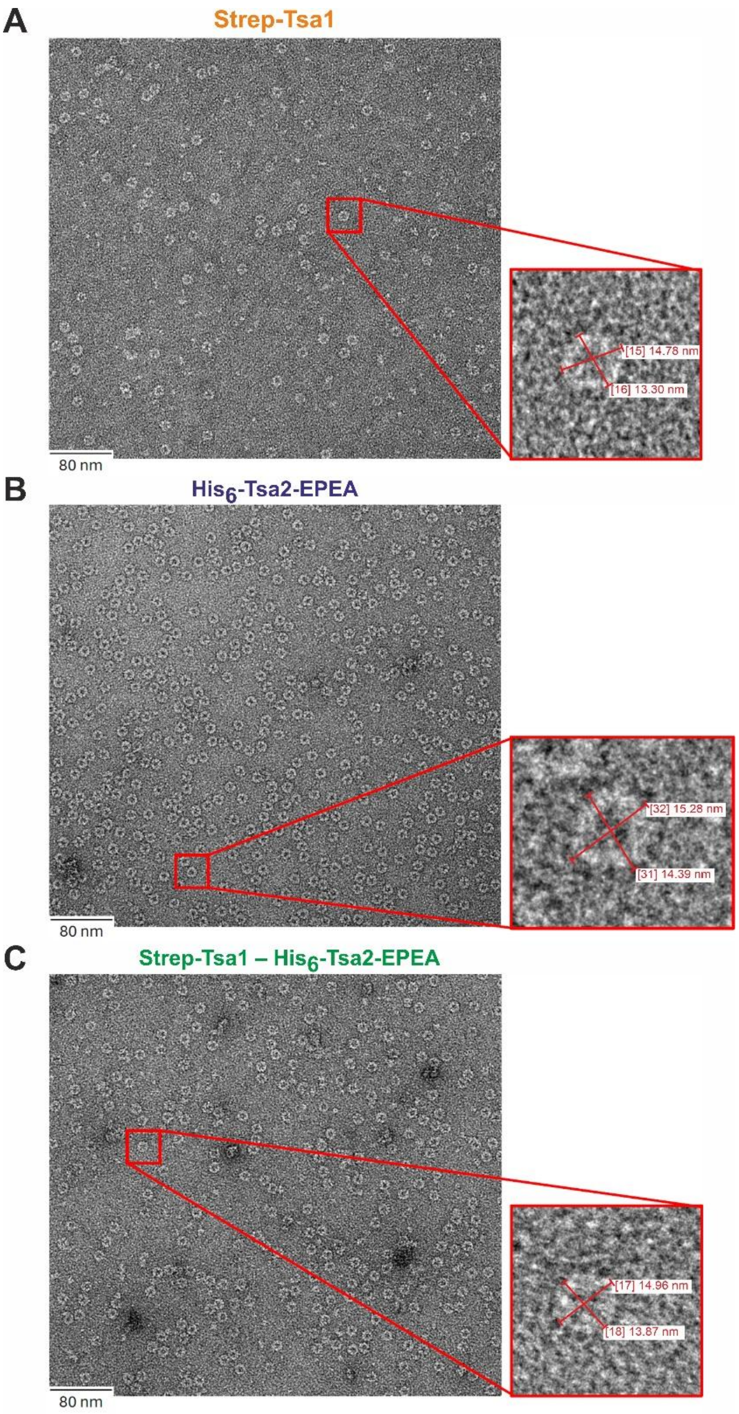

Supplementary Figure 5. The presence of the N- and C-terminal tags does not affect the decameric stability of the proteins in solution

Protein particles appear bright against the 2% uranyl-acetate stain, display a characteristic decameric 'donut-like' structure. Diameters were measured using EMMenu from TVIPS imaging software. Protein concentration was 0.02 mg/mL. **A)** Strep-Tsa1 homooligomer **B)** His_6-Tsa2-EPEA homooligomer **C)** Strep-Tsa1–His_6-Tsa2-EPEA heterooligomer. Experiments was repeated three times with separate protein purifications, each yielding similar results.

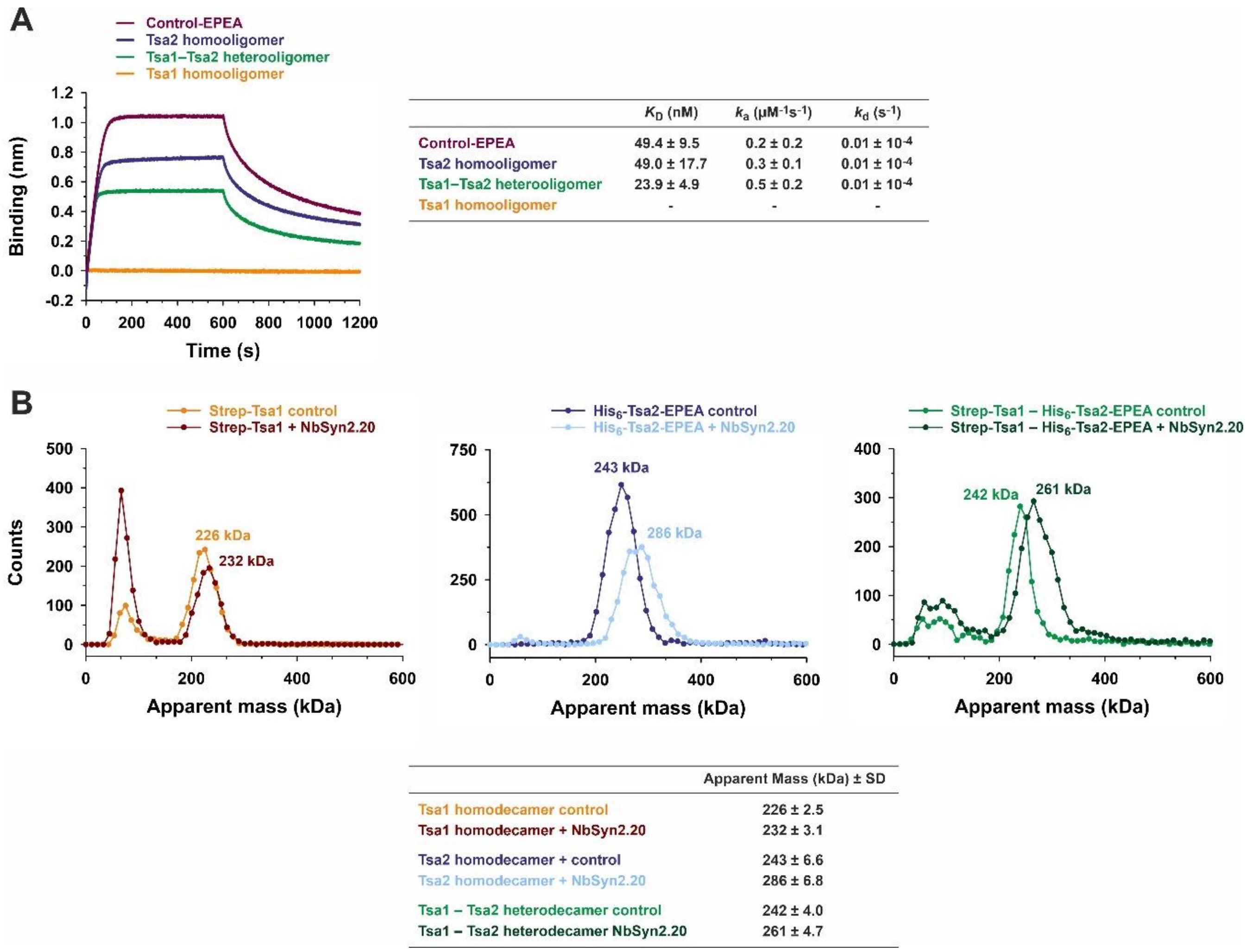

	K_D (nM)	k_a ($\mu M^{-1}s^{-1}$)	k_d (s^{-1})
Control-EPEA	49.4 ± 9.5	0.2 ± 0.2	0.01 ± 10^{-4}
Tsa2 homooligomer	49.0 ± 17.7	0.3 ± 0.1	0.01 ± 10^{-4}
Tsa1–Tsa2 heterooligomer	23.9 ± 4.9	0.5 ± 0.2	0.01 ± 10^{-4}
Tsa1 homooligomer	-	-	-

	Apparent Mass (kDa) ± SD
Tsa1 homodecamer control	226 ± 2.5
Tsa1 homodecamer + NbSyn2.20	232 ± 3.1
Tsa2 homodecamer + control	243 ± 6.6
Tsa2 homodecamer + NbSyn2.20	286 ± 6.8
Tsa1 – Tsa2 heterodecamer control	242 ± 4.0
Tsa1 – Tsa2 heterodecamer NbSyn2.20	261 ± 4.7

Supplementary Fig. 6. Nbsyn2.20 specifically binds to the C-terminal EPEA-tag of His_6-Tsa2 protein

BLI assay shows that Nbsyn2.20 specifically binds to the C-terminal EPEA-tag of Tsa2. Strep-Tsa1 homooligomer, His_6-Tsa2-EPEA homooligomer, and 0.88 mol/mol Strep-Tsa1–His_6-Tsa2-EPEA heterooligomer were immobilized on SA-sensors, with His-Nbsyn2.20 used as the analyte. Protein-EPEA was used as a positive control. Association and dissociation curves are shown (Binding (nm) vs time (s)) with Strep-Tsa1 homooligomer, His_6-Tsa2-EPEA homooligomer, 0.88 mol/mol Strep-Tsa1–His_6-Tsa2-EPEA heterooligomer and protein-EPEA represented in orange, blue, green and purple, respectively. Data were analyzed using Octet Analysis studio 13.0 software, and the graph was generated with GraphPad Software, Inc. Triplicates were measured. **B)** Mass photometry experimental data show that at least two Nbsyn2.20 molecules bind to the 0.88 mol/mol Strep-Tsa1–His_6-Tsa2-EPEA heterooligomer. Stock protein solutions (5 µM) were diluted prior to the measurement. Data were acquired for 60 s, and counts of individual molecules were plotted against their molecular weight. Strep-Tsa1 and His_6-Tsa2-EPEA homooligomers mass photometry histograms are shown in orange and blue, respectively, while the histogram for 0.88 mol/mol Strep-Tsa1–His_6-Tsa2-EPEA heterooligomer is shown in green. Data were analyzed using DiscoverMP (version 2.1.1; Refeyn Ltd). Triplicates were measured, and the average molecular weight was calculated.

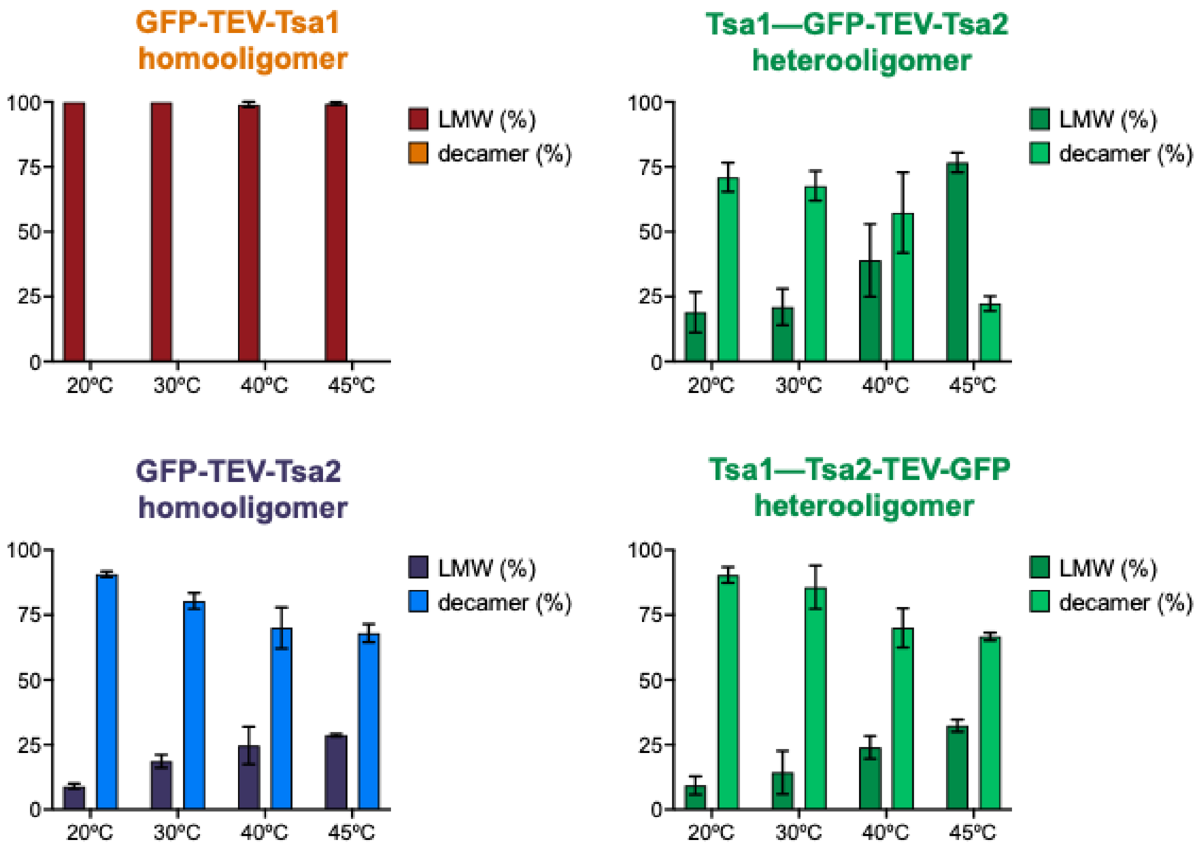

Supplementary Figure 7. Increasing temperature destabilizes the decamer conformation in all the proteins with a more evident shift on Tsa1.

Protein samples (5 µM) were incubated at the indicated temperatures for 15 minutes, then diluted and analyzed by mass photometry. Data were acquired over 60 seconds and processed using DiscoverMP (v2.1.1; Refeyn Ltd). The relative abundance of low-molecular-weight (LMW) and decameric species was quantified using default settings. All measurements were performed in triplicate (technical repeats with the same batch of purified proteins) and results are reported as percentage distributions across temperatures.

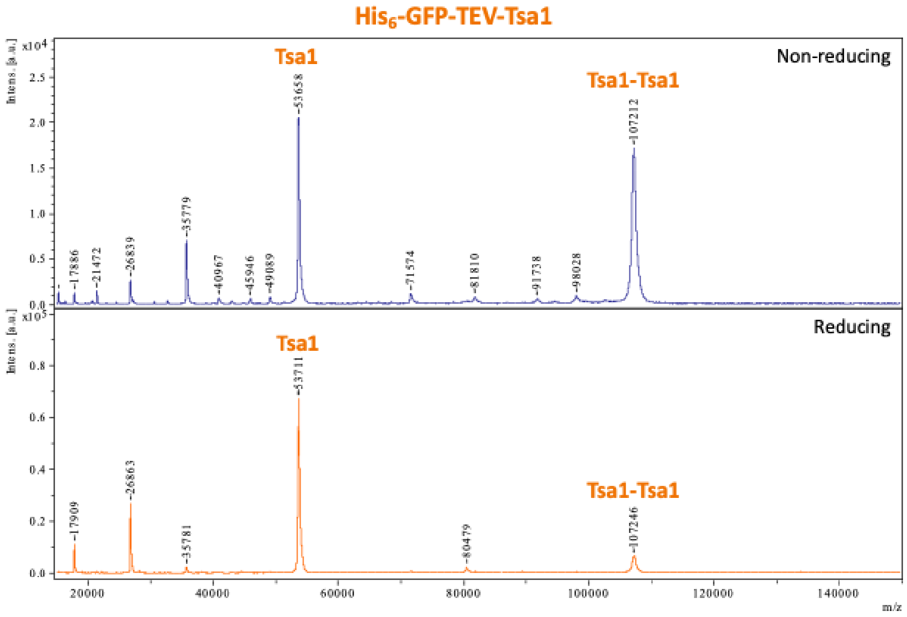

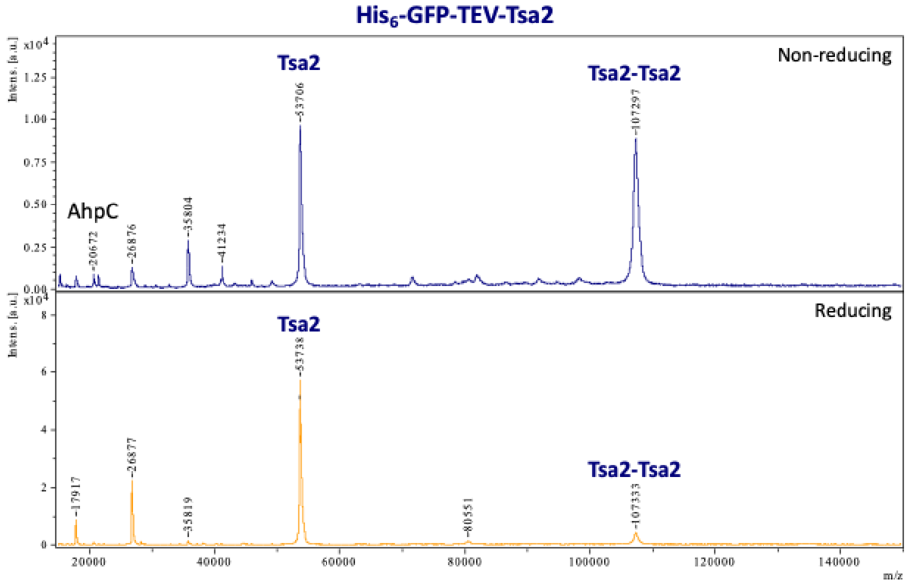

Supplementary Fig. 8. Purified His$_6$-GFP-TEV-Tsa1 homooligomer and His$_6$-GFP-TEV-Tsa2 homooligomer samples present disulfide-linked dimers, which are mainly reduced by DTT treatment. His$_6$-GFP-TEV-Tsa2 show AhpC contamination, however His$_6$-GFP-TEV-Tsa1 does not. The ratio of each heterooligomer sample is displayed above. The signal intensity (AU) is plotted against different m/z ratios. Duplicate measurements were performed for each sample, and data were analyzed by FlexAnalysis 3.4 (Bruker). The expected masses are displayed in **Supplementary Table 1**.

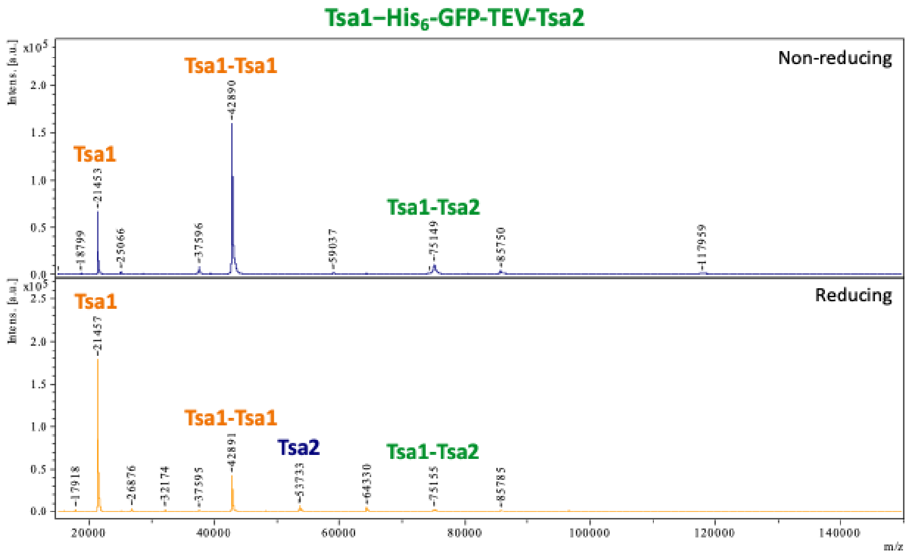

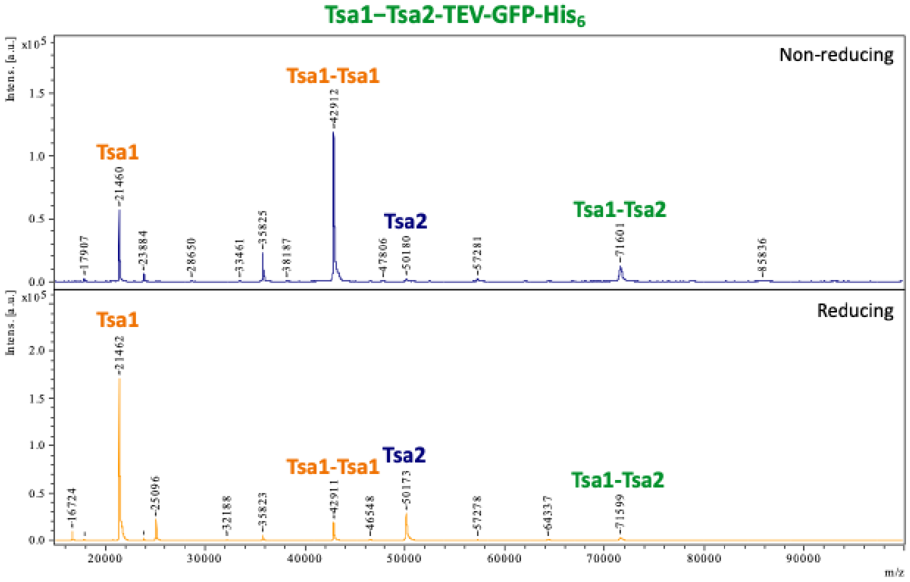

Supplementary Fig. 9. Purified Tsa1–His$_6$-GFP-TEV-Tsa2 and Tsa1–Tsa2-TEV-GFP-His$_6$ heterooligomer samples present disulfide-linked heterodimers, which are mainly reduced by DTT treatment. Tsa1–Tsa2-TEV-GFP-His$_6$ show AhpC contamination, however Tsa1–His$_6$-GFP-TEV-Tsa2 does not. The ratio of each heterooligomer sample is displayed above. The signal intensity (AU) is plotted against different m/z ratios. Duplicate measurements were performed for each sample, and data were analyzed by FlexAnalysis 3.4 (Bruker). The expected masses are displayed in **Supplementary Table 1**.

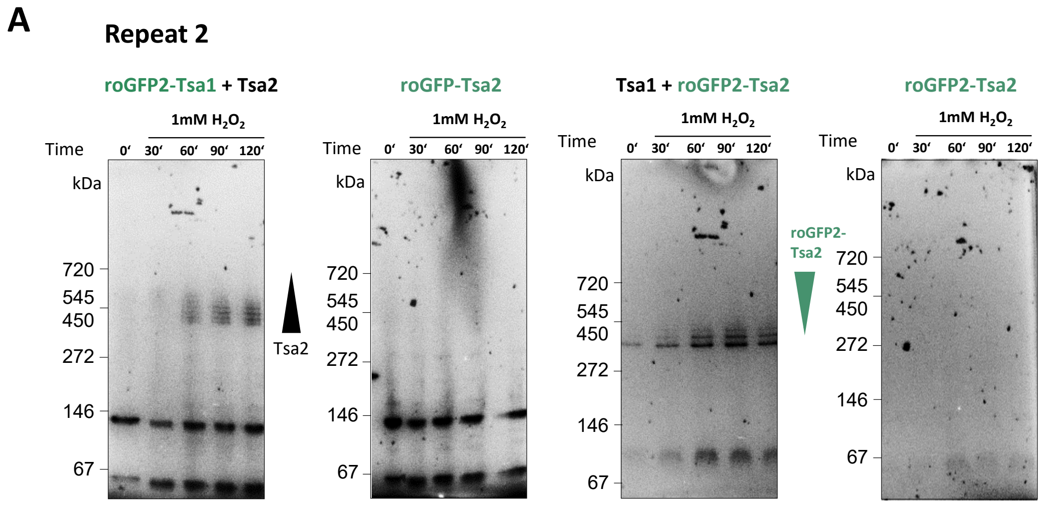

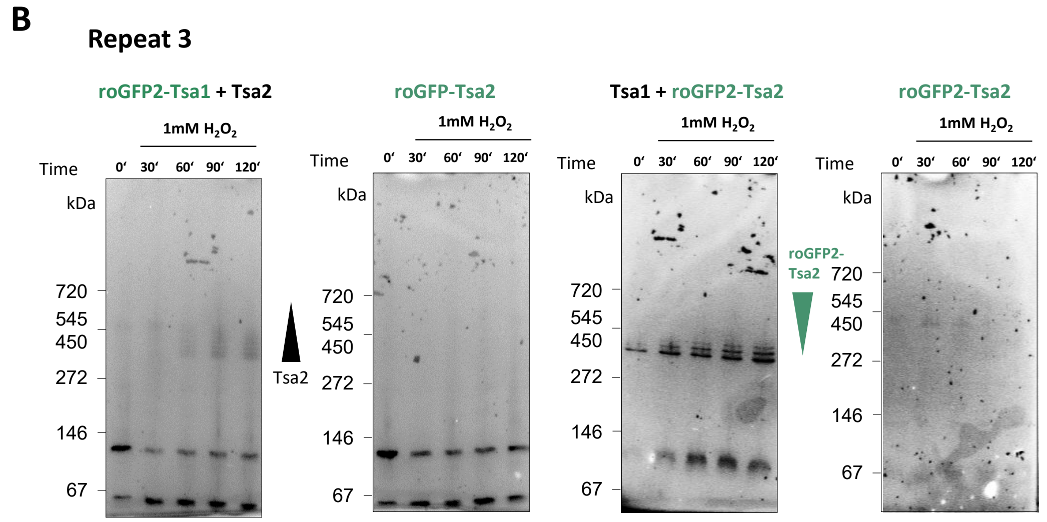

Supplementary Figure 10. Heterooligomerization is inducible in yeast and promotes decamer stabilization

A. and **B.** Experimental repeats of the CN-PAGE gels presented in **Fig. 3B.**

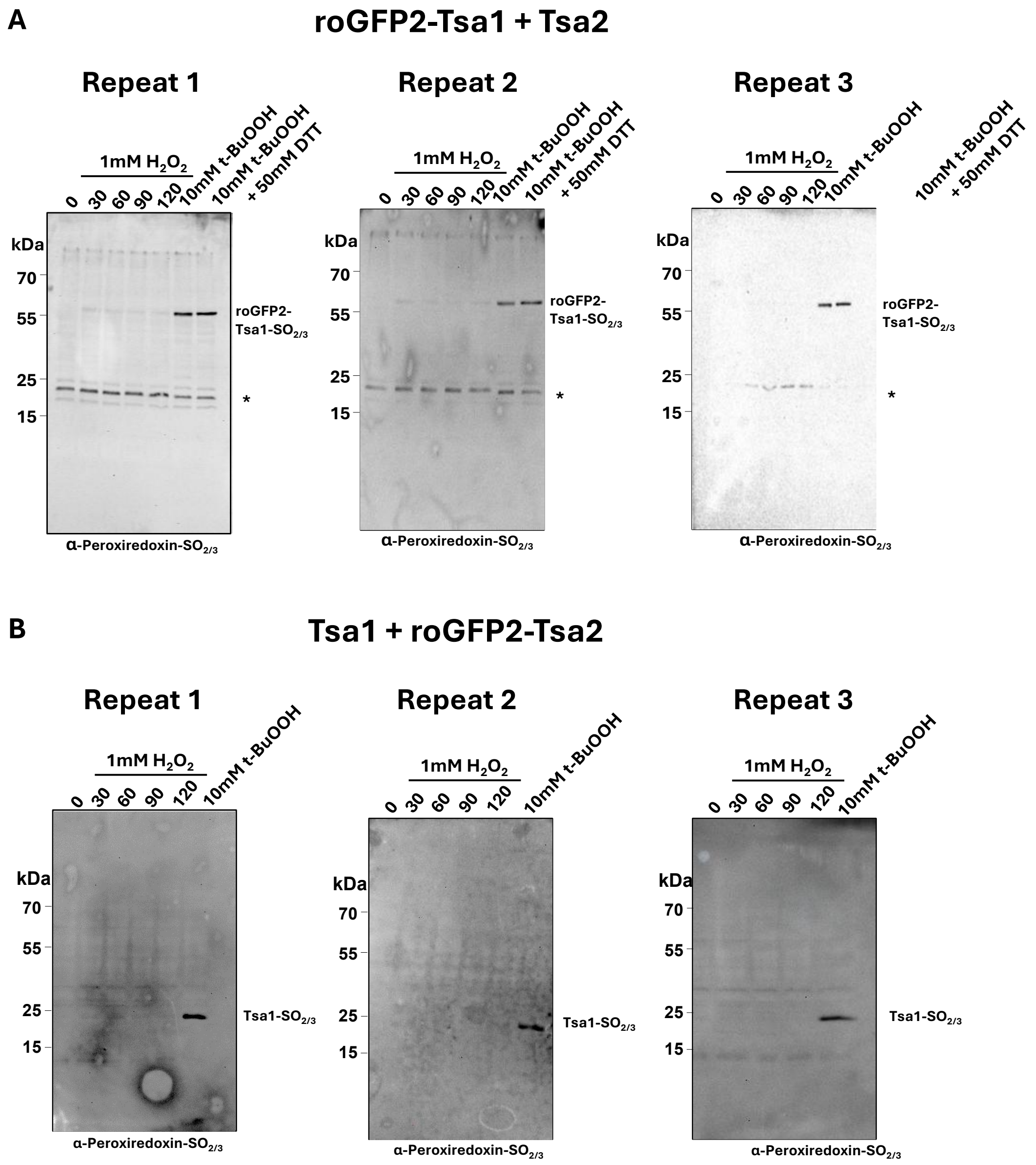

Supplementary Figure 11. Exogenous H_2O_2 induces *TSA2* expression but does not induce hyperoxidation

A, B. Samples taken from three independent experimental repeats of the yeast *TSA2* induction experiment in **Fig. 3B** with roGFP2-Tsa1 and Tsa2 expressing cells (**A**) and Tsa1 and roGFP2-Tsa2 expressing cells (**B**) were run on non-reducing SDS-PAGE gels and analyzed by western blot with an α-Prx-$SO_{2/3}$ antibody. The '*' indicates the position of a non-specific band in the panels in **A**. An alternative α-Prx-$SO_{2/3}$ antibody was used in (**B**), with no non-specific bands observed.

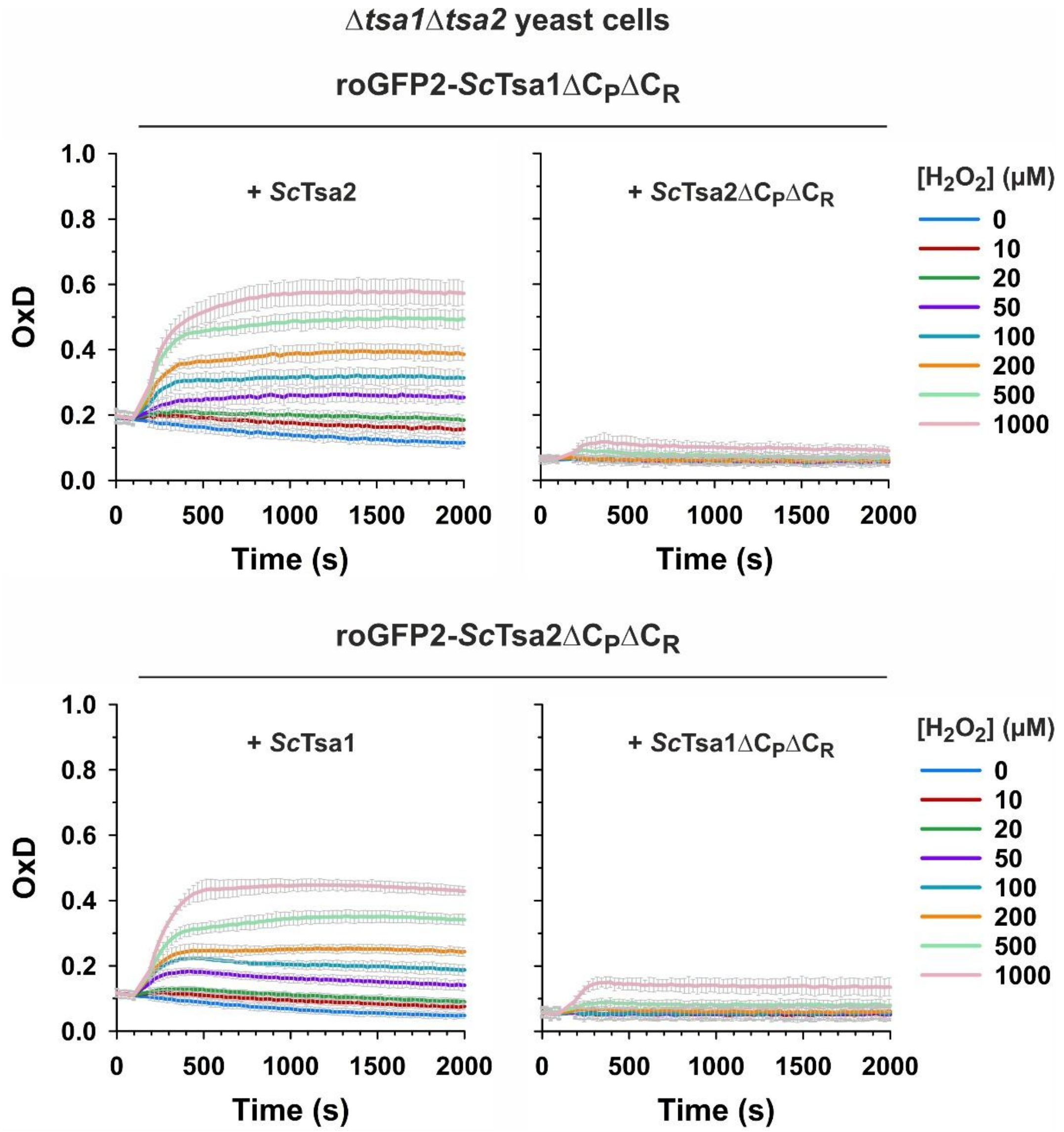

Supplementary Figure 12. Tsa1 and Tsa2 form enzymatically active heterooligomers in yeast

Graphs showing the change in the degree of oxidation (OxD) in response to 1 mM H_2O_2 of roGFP2-Tsa1ΔC_PΔC_R and roGFP2-Tsa1ΔC_PΔC_R constructs expressed in Δ*tsa1*Δ*tsa2* yeast together with either a wild-type (wt) or cysteine-less (ΔC_PΔC_R) variant of the corresponding partner peroxiredoxin. Experiments were repeated 3 times with independent yeast cultures. Data are presented as mean ± s.d.

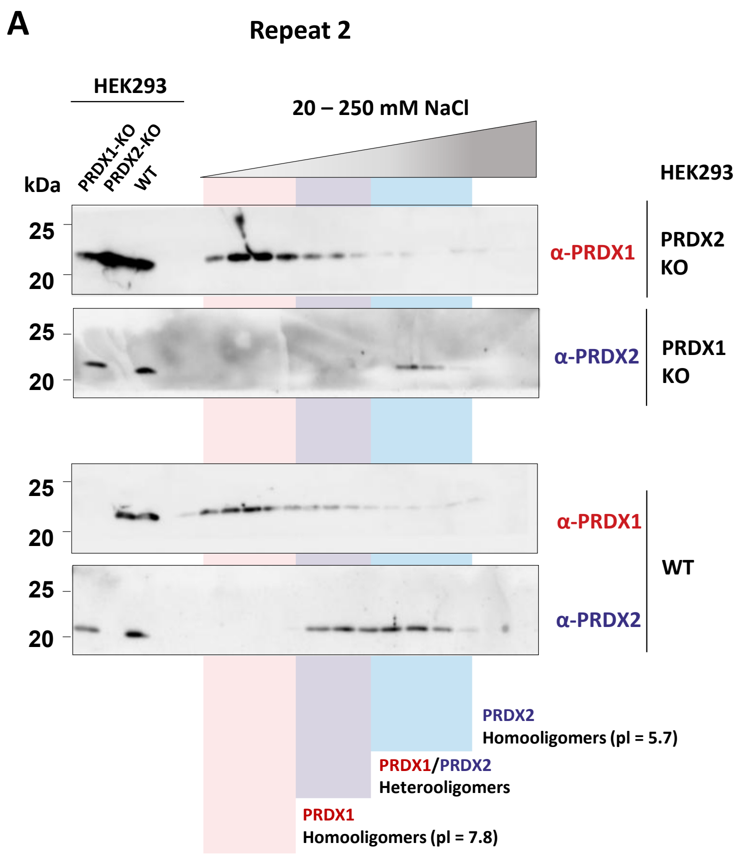

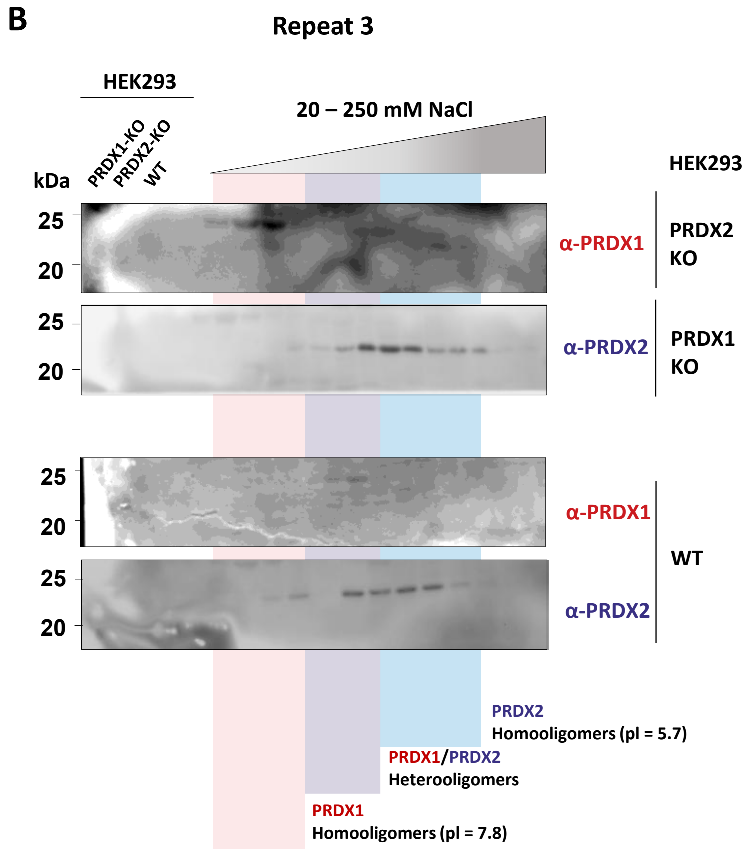

Supplementary Figure 13. PRDX1 and PRDX2 form heterooligomers in HEK293T cells.

A and **B** Second and third experimental repeats of the experiment presented in Figure 6A,B.

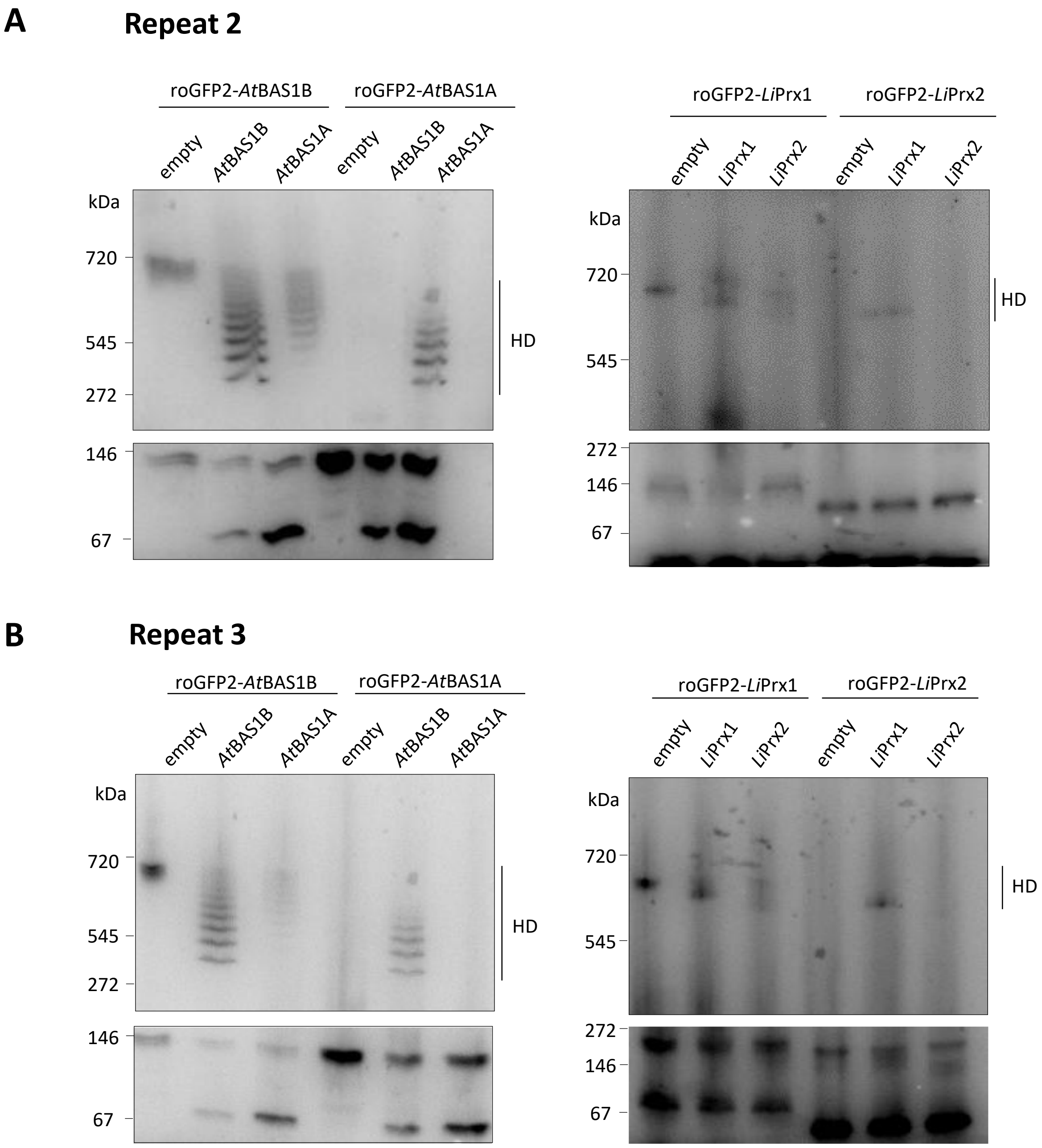

Supplementary Figure 14 Heterooligomerization changes the dimer–decamer equilibrium of Arabidopsis and Leishmania peroxiredoxins.

Two extra experimental repeats of the data presented in Figure 6B.

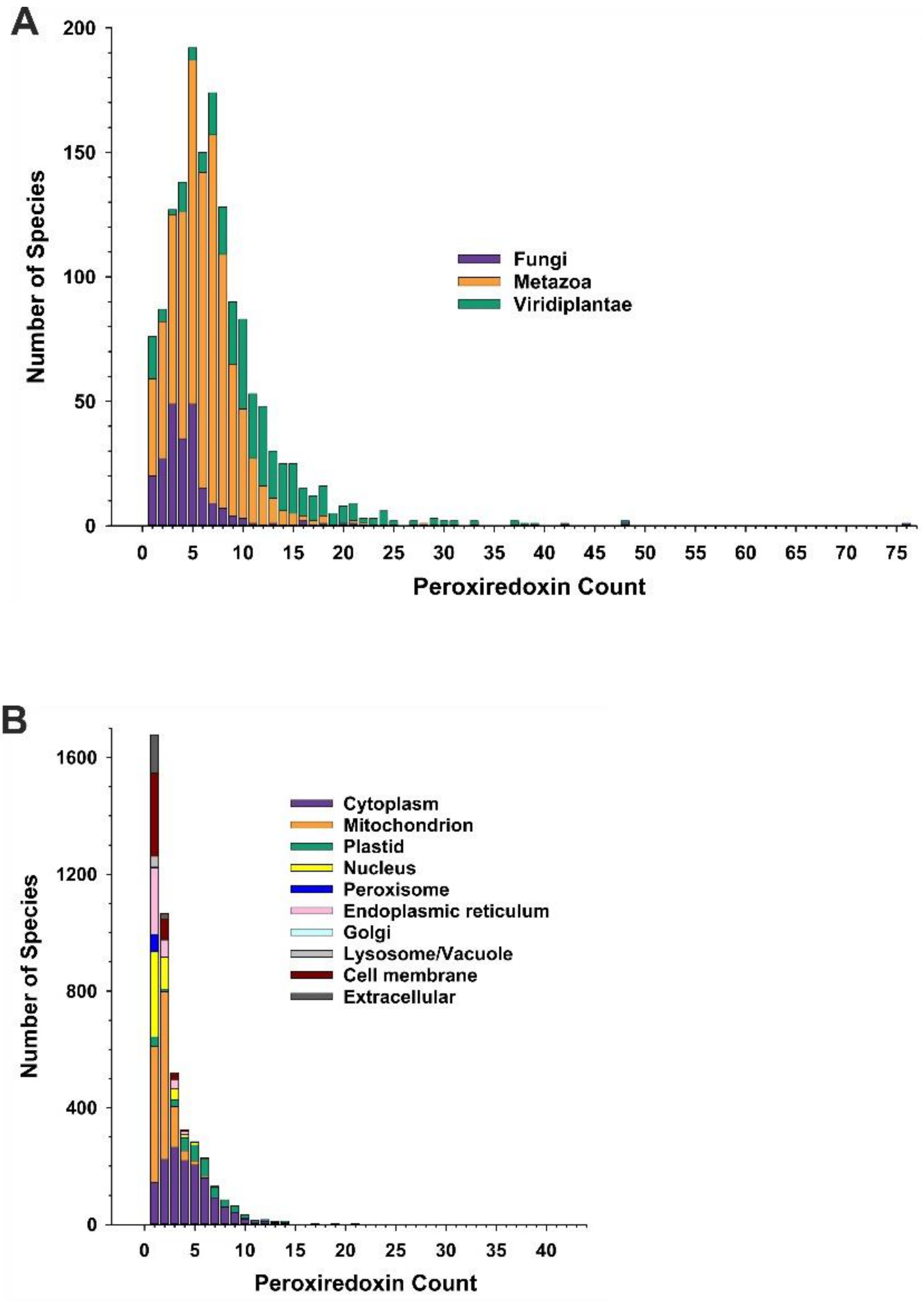

Supplementary Figure 15. Multiple Prx1/AhpC-type peroxiredoxin isoforms are found within at least one subcellular compartment in most eukaryotes.

Corresponds to main **Figure 6C**. **A**) Histogram showing the total number of Prx1/AhpC-type peroxiredoxins predicted within each of 1525 sampled eukaryotic species. **B**) Histogram showing the number of species in which there are predicted to be the indicated number of Prx1/AhpC-type peroxiredoxin present within the indicated subcellular compartments.

Source data for Supp Figure3a ‖ Source data for Supp Figure 3b ‖ Source data for Supp Figure 3c

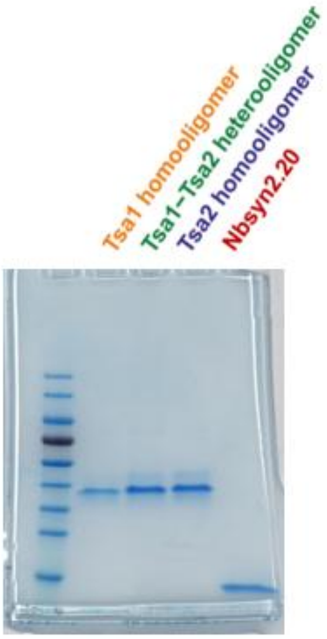

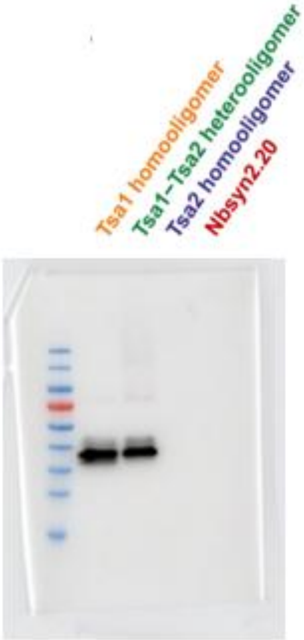

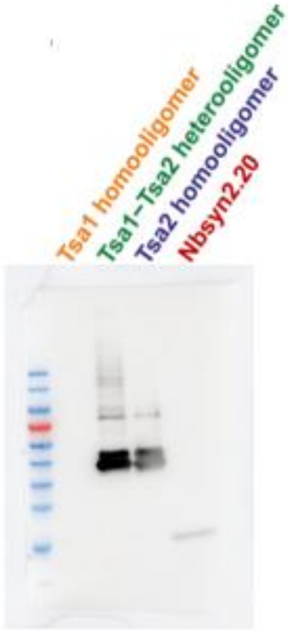

Source data for Supp Figure3d

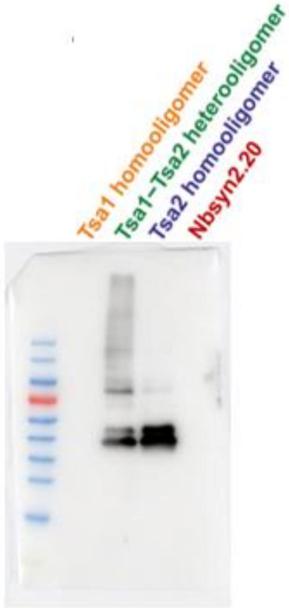

Source data for Supp Figure 3e

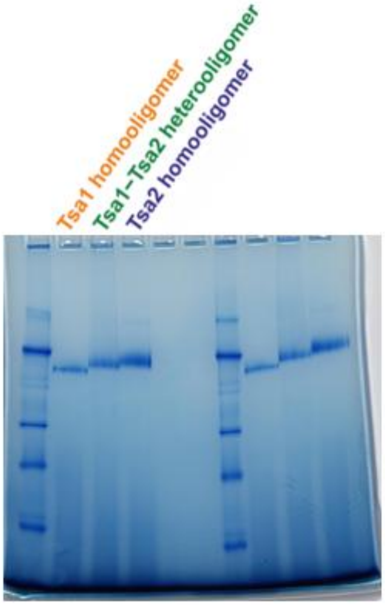

Source Data for Supplementary Figure 10a.

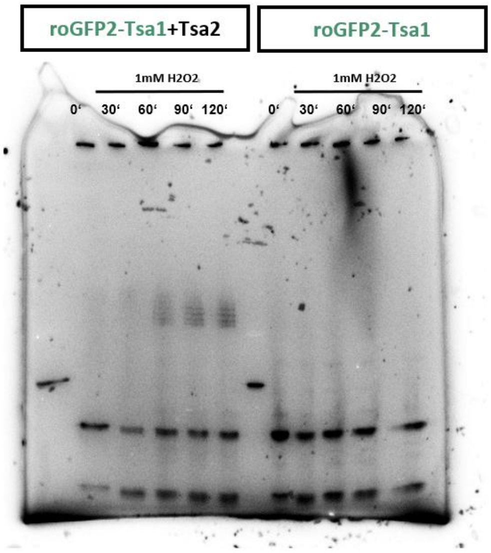

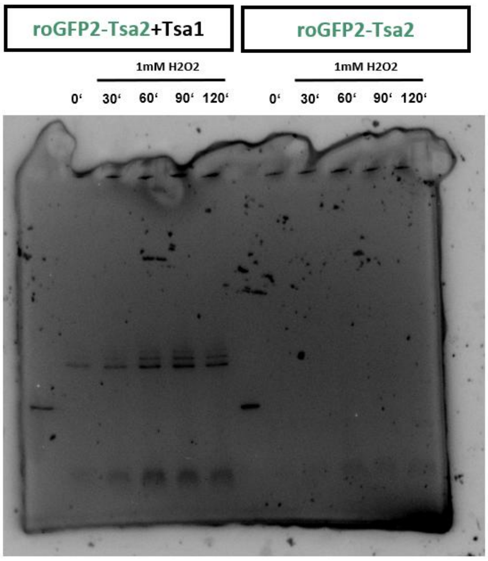

Source Data for Supplementary Figure 10b

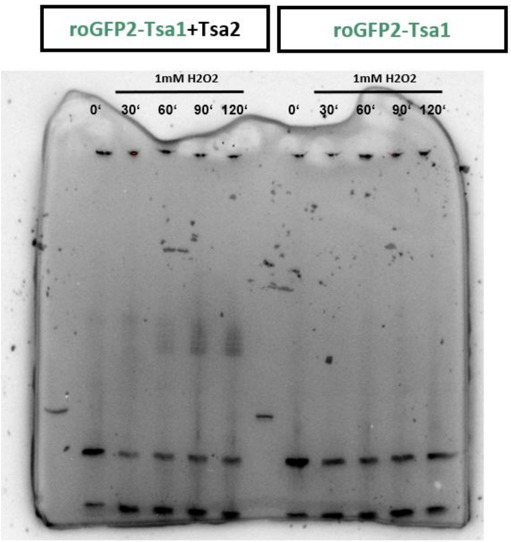

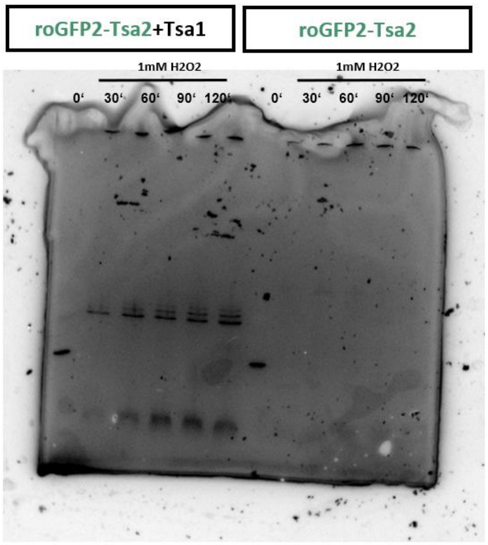

Source Data for Supplementary Figure 11a

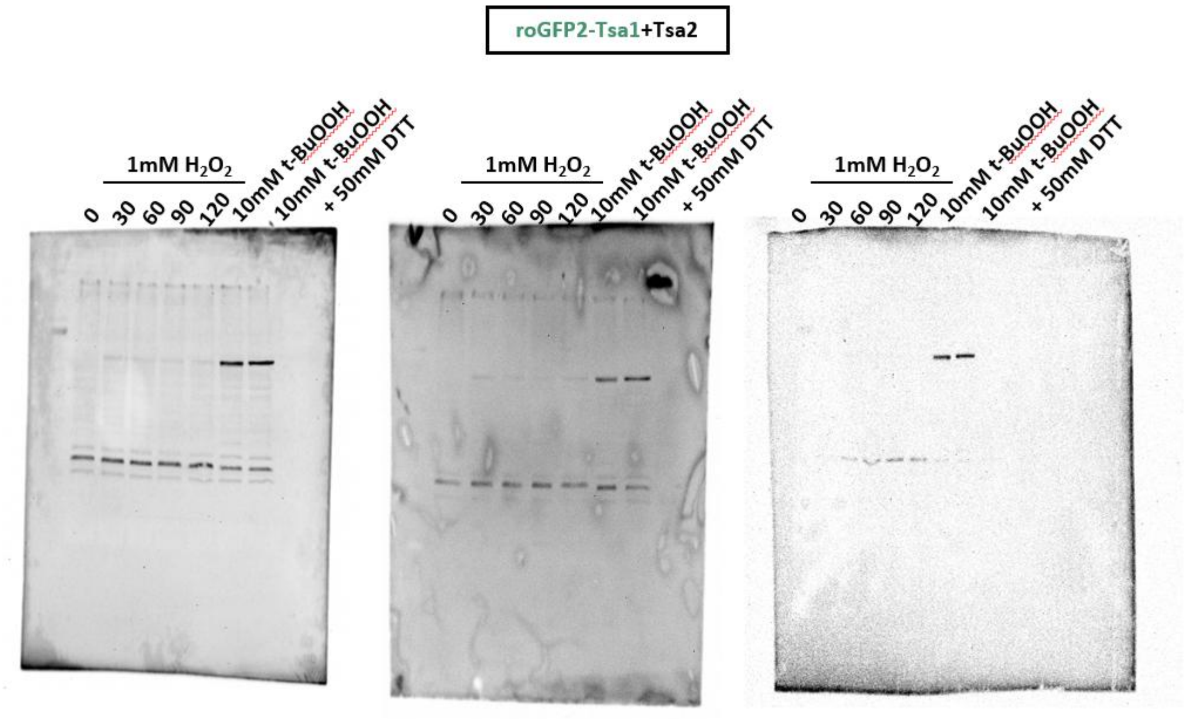

Source Data for Supplementary Figure 11b

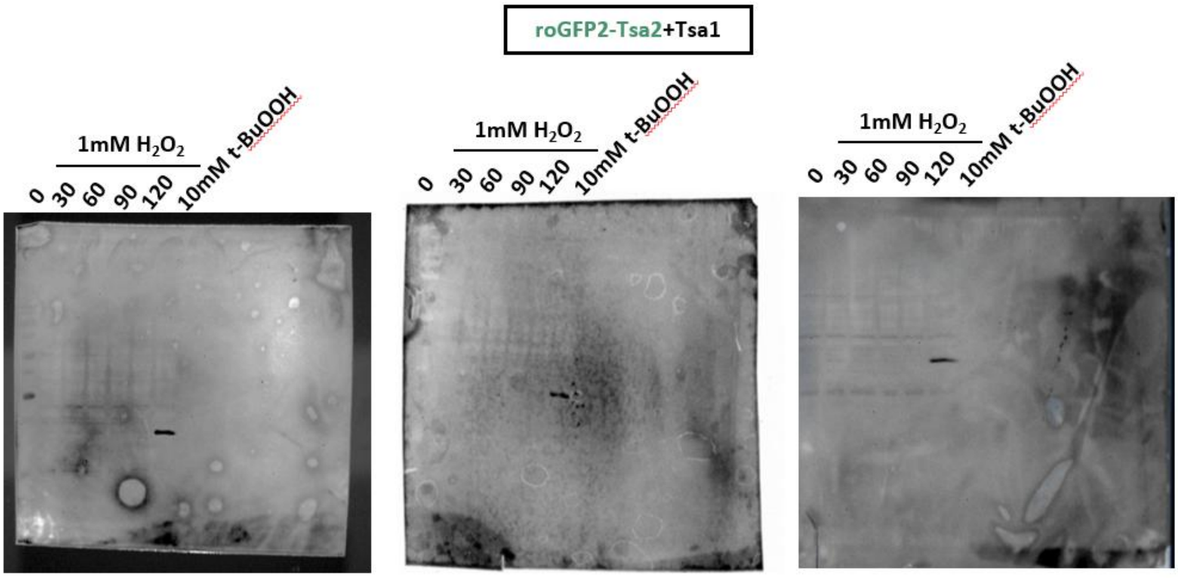

Source Data for Supplementary Figure 13a:

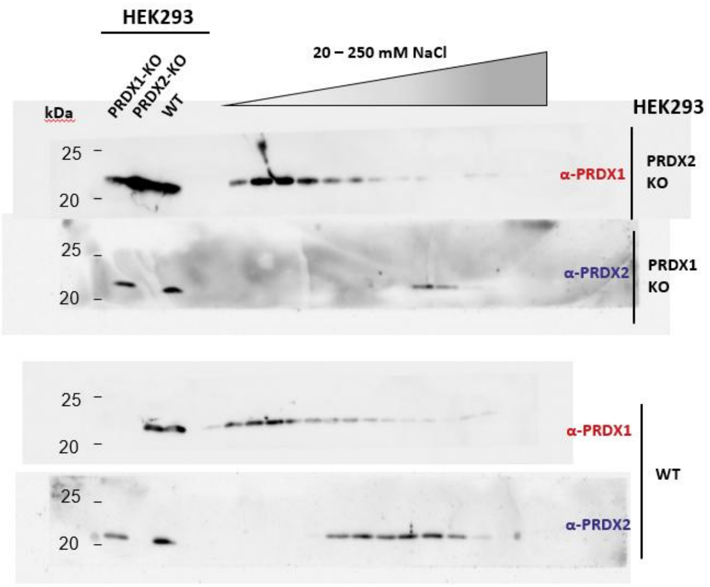

Source Data for Supplementary Figure 13b

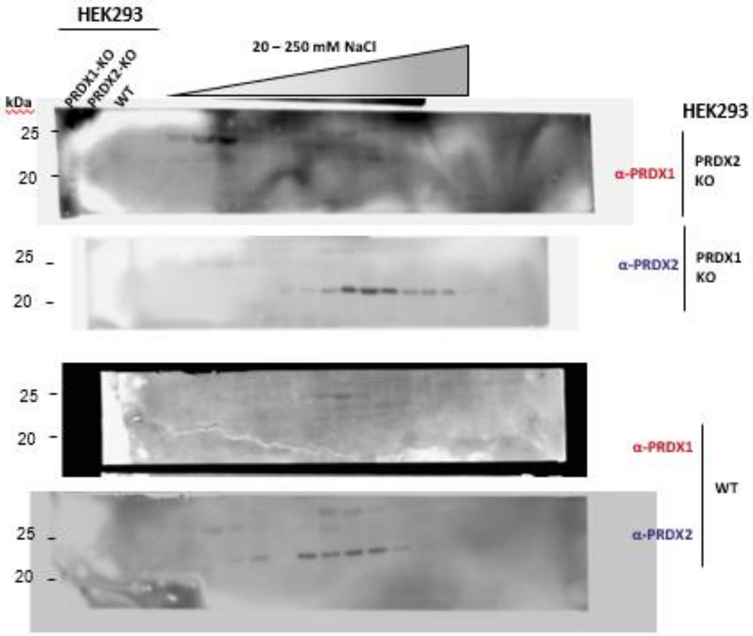

Source Data for Supplementary Figure 14a

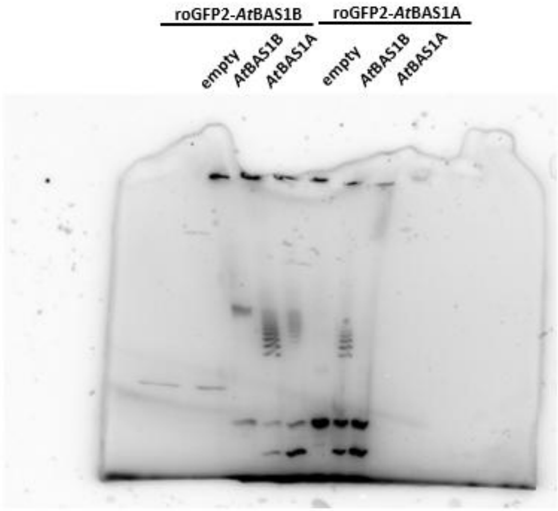

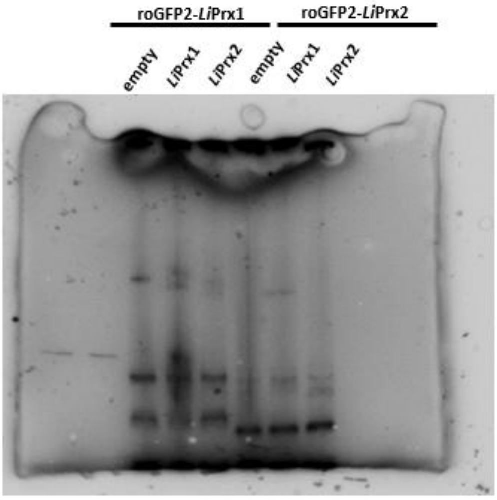

Source Data for Supplementary Figure 14b

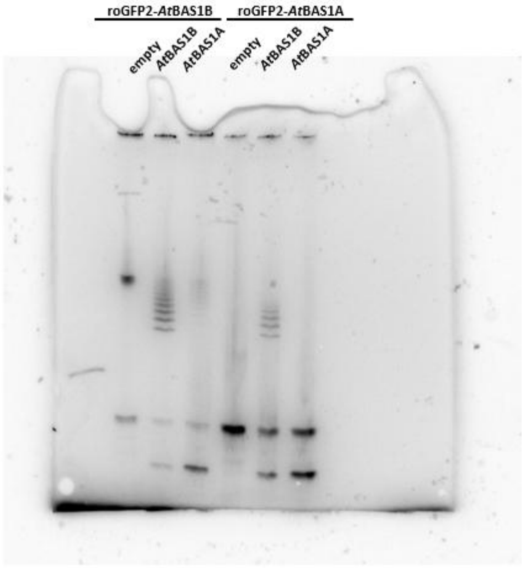

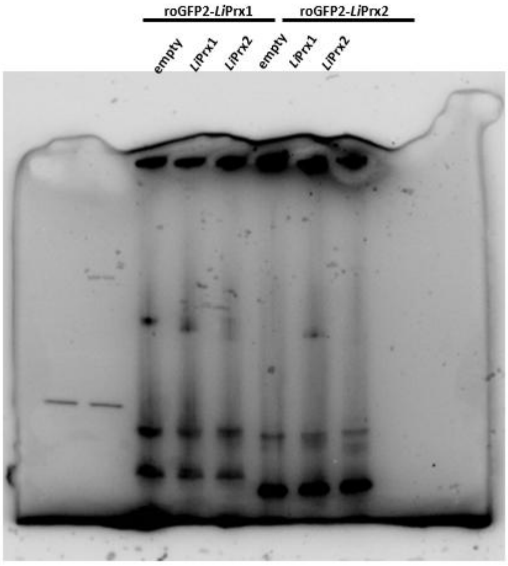

Supplementary Table 1. Expected molecular masses (Da) of the peroxiredoxin constructs analyzed in this study.

Protein	Oligomeric state	Molecular mass (Da)
Strep-Tsa1	monomer	22,916
Strep-Tsa1	homodimer	45,832
His_6-Tsa2-EPEA	monomer	24,204
His_6-Tsa2-EPEA	homodimer	48,408
Strep-Tsa1—His_6-Tsa2-EPEA	heterodimer	47,120
His_6-GFP-TEV-Tsa1	monomer	53,719
His_6-GFP-TEV-Tsa1	homodimer	107,438
His_6-GFP-TEV-Tsa2	monomer	53,744
His_6-GFP-TEV-Tsa2	homodimer	107,488
Tsa1 (P34760)	monomer	21,590
Tsa1 (P34760)	homodimer	43,180
Tsa1—His_6-GFP-TEV-Tsa2	heterodimer	75,334
Tsa2-TEV-GFP- His_6	monomer	50,336
Tsa2-TEV-GFP- His_6	homodimer	100,672
Tsa1—Tsa2-TEV-GFP- His_6	heterodimer	71,926
AhpC (P0AE08)	monomer	20,761
AhpC (P0AE08)	homodimer	41,522

Supplementary Table 2. Mass photometry analysis of the temperature-dependent dimer–dcamer abundance of the indicated Tsa1 and Tsa2 constructs.

Temperature (°C)	LMW (kDa, mean ± SD; %)	Decamer (kDa, mean ± SD; %)	Decamer p2 (kDa, mean ± SD; %)
Strep-Tsa1			
20	78.7 ± 7.5 (33.7%)	225 ± 5.3 (66.0%)	
30	70.3 ± 9.5 (47.0%)	220.3 ± 4.7 (51.7%)	
40	66.5 ± 14.8 (74.5%)	190.5 ± 7.8 (24.5%)	
45	64.7 ± 6.1 (92.7%)	199.3 ± 15.3 (7.0%)	
His6-Tsa2-EPEA			
20	47 (1.0%)	235 ± 4.6 (98.3%)	
30	54 (1.3%)	229.3 ± 14.8 (93.3%)	
40	–	199.3 ± 11.7 (97.3%)	
45	48 (1.3%)	204 ± 7.5 (95.7%)	
Strep-Tsa1–His6-Tsa2-EPEA			
20	74.7 ± 10.5 (16.7%)	215.7 ± 4.9 (76.7%)	
30	86.3 ± 12.2 (11.0%)	207 ± 9.2 (80.3%)	
40	85 ± 28.6 (18.3%)	200.3 ± 8.3 (79.3%)	
45	67.2 ± 5.3 (35.0%)	201.7 ± 8.1 (64.3%)	
His6-GFP-TEV-Tsa1			
20	112.7 ± 10.2 (100.0%)	–	
30	104.3 ± 1.5 (100.0%)	–	
40	100.7 ± 7.4 (99.0%)	–	
45	101 ± 6.2 (99.3%)	–	
His6-GFP-TEV-Tsa2			
20	112 ± 12.5 (9.0%)	465 ± 3.8 (90.7%)	
30	104.3 ± 17.1 (18.7%)	456.7 ± 4.9 (80.3%)	
40	98 ± 20.1 (24.7%)	416.3 ± 4.2 (70.0%)	
45	98.3 ± 19.7 (28.7%)	434.3 ± 12.2 (68.0%)	
Tsa1–His6-GFP-TEV-Tsa2			
20	93.7 ± 8.1 (19.0%)	243.7 ± 7.5 (71.0%)	434.3 ± 55.2 (9.7%)
30	119.8 ± 24.4 (21.0%)	248 ± 1.4 (67.7%)	387 ± 8.5 (10.0%)
40	102.3 ± 12.0 (39.0%)	256.7 ± 9.5 (57.3%)	–
45	77.3 ± 5.1 (76.7%)	256.7 ± 17.2 (22.3%)	–
Tsa1–Tsa2-TEV-GFP-His6			
20	74 ± 10.5 (9.3%)	286 ± 4.0 (90.3%)	
30	67.7 ± 6.4 (14.3%)	289.7 ± 2.5 (85.7%)	
40	68.7 ± 6.1 (24.0%)	277.2 ± 9.3 (70.0%)	
45	61 ± 2.0 (32.3%)	294 ± 7.8 (66.7%)	

Supplementary Table 3. Rate constants for the redox reactions of recombinant Tsa1 and/or Tsa2 at pH7.4 and 25°C.

Protein	Assigned reaction partners A/B and products P/Q				Rate constant	
	A	+ B	P	+ Q	k	
Tsa1	His$_6$-Tsa1(SH)$_2$	H_2O_2	His$_6$-Tsa1(SOH)$_{ff}$	H_2O	k_{1ox}	$(1.2 \pm 0.03) \times 10^{8}\ M^{-1}s^{-1}$
Tsa1	His$_6$-Tsa1(SH)$_2$ [a]	H_2O_2	His$_6$-Tsa1(SOH)$_{ff}$ [a]	H_2O	k_{1ox}	$(9.7 \pm 0.4) \times 10^{7}\ M^{-1}s^{-1}$ [a]
Tsa1	Strep-Tsa1(SH)$_2$	H_2O_2	Strep-Tsa1(SOH)$_{ff}$	H_2O	k_{1ox}	$8.1 \times 10^{7}\ M^{-1}s^{-1}$ [b]
Tsa2	His$_6$-Tsa2(SH)$_2$	H_2O_2	His$_6$-Tsa2(SOH)$_{ff}$	H_2O	k_{1ox}	$(1.3 \pm 0.1) \times 10^{7}\ M^{-1}s^{-1}$
Tsa1/2	His$_6$-Tsa1(SH)$_2$/	H_2O_2	His$_6$-Tsa1(SOH)$_{ff}$/	H_2O	k_{1ox}	$6.1 \times 10^{7}\ M^{-1}s^{-1}$
	Strep-Tsa2(SH)$_2$		Strep-Tsa2(SOH)$_{ff}$		k_{1ox}	$2.8 \times 10^{7}\ M^{-1}s^{-1}$
Tsa1+Tsa2	His$_6$-Tsa1(SH)$_2$ +	H_2O_2	His$_6$-Tsa1(SOH)$_{ff}$ +	H_2O	k_{1ox}	$9.8 \times 10^{7}\ M^{-1}s^{-1}$
	His$_6$-Tsa2(SH)$_2$		His$_6$-Tsa2(SOH)$_{ff}$		k_{1ox}	$1.2 \times 10^{7}\ M^{-1}s^{-1}$
Tsa1	His$_6$-Tsa1(SOH)$_{ff}$		His$_6$-Tsa1(SOH)$_{lu}$		k_{2ox}	$70 \pm 9\ s^{-1}$
Tsa1	His$_6$-Tsa1(SOH)$_{ff}$ [a]		His$_6$-Tsa1(SOH)$_{lu}$ [a]		k_{2ox}	$64 \pm 5\ s^{-1}$ [a]
Tsa1	Strep-Tsa1(SOH)$_{ff}$		Strep-Tsa1(SOH)$_{lu}$		k_{2ox}	$67\ s^{-1}$ [b]
Tsa2	His$_6$-Tsa2(SOH)$_{ff}$		His$_6$-Tsa2(SOH)$_{lu}$		k_{2ox}	$51 \pm 11\ s^{-1}$
Tsa1/2	His$_6$-Tsa1(SOH)$_{ff}$/		His$_6$-Tsa1(SOH)$_{lu}$/		k_{2ox}	$45 \pm 7\ s^{-1}$
	Strep-Tsa2(SOH)$_{ff}$		Strep-Tsa2(SOH)$_{lu}$			
Tsa1	His$_6$-Tsa1(SOH)$_{lu}$		His$_6$-Tsa1(S$_2$)	H_2O	k_{3ox}	$6.6 \pm 1.7\ s^{-1}$
Tsa1	His$_6$-Tsa1(SOH)$_{lu}$ [a]		His$_6$-Tsa1(S$_2$) [a]	H_2O	k_{3ox}	$4.6 \pm 0.3\ s^{-1}$ [a]
Tsa1	Strep-Tsa1(SOH)$_{lu}$		Strep-Tsa1(S$_2$)	H_2O	k_{3ox}	$6.8\ s^{-1}$ [b]
Tsa2	His$_6$-Tsa2(SOH)$_{lu}$		His$_6$-Tsa2(S$_2$)	H_2O	k_{3ox}	$4.0 \pm 0.1\ s^{-1}$
Tsa1/2	His$_6$-Tsa1(SOH)$_{lu}$/		His$_6$-Tsa1(S$_2$)/	H_2O	k	$3.6 \pm 0.6\ s^{-1}$
	Strep-Tsa2(SOH)$_{lu}$		Strep-Tsa1(S$_2$)			
Tsa1	His$_6$-Tsa1(S$_2$) [b]	Trx1(SH)$_2$	His$_6$-Tsa1-SS-Trx1 [b]		k_{1red}	$(2.8 \pm 0.5) \times 10^{6}\ M^{-1}s^{-1}$
Tsa2	His$_6$-Tsa2(S$_2$)	Trx1(SH)$_2$	His$_6$-Tsa2-SS-Trx1		k_{1red}	$(3.9 \pm 0.1) \times 10^{6}\ M^{-1}s^{-1}$
Tsa1	? His$_6$-Tsa1-SS-Trx1		? His$_6$-Tsa1(SH)$_{2\ lu}$	Trx1(S$_2$)	k_{2red}	$10 \pm 2\ s^{-1}$
Tsa2	? His$_6$-Tsa2-SS-Trx1		? His$_6$-Tsa2(SH)$_{2\ lu}$	Trx1(S$_2$)	k_{2red}	$9.4 \pm 0.7\ s^{-1}$
Tsa1	? His$_6$-Tsa1(SH)$_{2\ lu}$		? His$_6$-Tsa1(SH)$_{2\ ff}$		k_{3red}	$0.6 \pm 0.1\ s^{-1}$

[a] 39; [b] 38

Supplementary Table 4. H_2O_2-dependent steady-state rate constants for Tsa1, Tsa2 and Tsa1–Tsa2 heterooligomers in Extended Figure 3

Enzyme	[Trx1] (μM)	$k_{cat}^{app}{}_{(H2O2)}$ (s^{-1})	$K_m^{app}{}_{(H2O2)}$ (μM)	$k_{cat}^{app}/K_m^{app}{}_{(H2O2)}$ ($M^{-1}s^{-1}$)
Tsa1	5	6.3±0.6	0.32±0.66	(1.9±1.0) × 10^7
	10	10.1±0.1	0.75±0.12	(1.3±0.2) × 10^7
	15	13.2±0.9	0.93±0.24	(1.4±0.4) × 10^7
Tsa2	5	19.2±3.0	0.46±0.16	(4.2±0.9) × 10^7
	10	26.5±2.6	0.72±0.15	(3.7±0.4) × 10^7
	15	29.9±2.8	0.64±0.34	(4.7±3.8) × 10^7
Tsa1/2	5	26.4±2.4	0.27±0.31	(9.9±8.4) × 10^7
	10	40.8±2.0	0.43±0.51	(1.0±2.2) × 10^8
	15	49.0±4.5	0.44±0.49	(1.1±6.5) × 10^8

Supplementary Table 5. Rate constants for yeast Trx1 from secondary plots in Extended Figure 3f for Tsa1, Tsa2 and Tsa1–Tsa2 heterooligomers

Enzyme	$k_{cat(Trx1)}$ (s^{-1})	$K_{m(Trx1)}$ (μM)	$k_{cat}/K_{m(Trx1)}$ ($M^{-1}s^{-1}$)
Tsa1	24±16	13±14	(1.8±0.6) × 10^6
Tsa2	41±6	6±2	(6.8±2.3) × 10^6
Tsa1/2	86±3	11±2	(7.5±1.0) × 10^6

Supplementary Table 6. Primers used in this study

Plasmid	Encoded Protein	Primers for cloning/mutagenesis
pET15b/His-*TSA1*	MGSSH_6SSGLVPRGSHM-Tsa1	**Ref. [27]**
pET15b/His-*TSA2*	MGSSH_6SSGLVPRGSHM-Tsa2	**Ref. [27]**
pET45b/Strep2-*TSA1*	MAWSHPQFEKGGT-Tsa1	S: GATC**GGTACC**GTCGCTCAAGTTCAAAAGCAAG<br>AS: GATC**CCTAGG**TTATTTGTTGGCAGCTTCGAAG
pColaDuet/Strep2-<br>*TSA1*/His-*TSA2*	MAWSHPQFEKGGT-Tsa1<br>MGSSH_6SSGLVPRGSHM-Tsa2	S: GATC**CATATG**GCATGGTCTCATCCGCAGTTTG<br>AS: GATC**CTCGAG**TTATTTGTTGGCAGCTTCGAAG
pET45b/Strep2-*TSA1*ΔC_R	MAWSHPQFEKGGT-Tsa1ΔC_R	S: GGTACTGTCTTGCCATCTAACTGGACTCCAGGTG<br>AS: CACCTGGAGTCCAGTTAGATGGCAAGACAGTACC
pColaDuet/Strep2-<br>*TSA1*ΔC_R /His-*TSA2*	MAWSHPQFEKGGT-Tsa1ΔC_R<br>MGSSH_6SSGLVPRGSHM-Tsa2	-
pColaDuet/Strep2-<br>*TSA1*/His-*TSA2-EPEA*	MAWSHPQFEKGGT-Tsa1<br>MGSSH_6SSGLVPRGSHM-Tsa2-<br>EPEA	S:<br>GATC**CCATGG**GCAGCAGCCATCATCATCATCATCACAGCA<br>AS:<br>GATC**GGATCC**TTATGCTTCCGGTTCATTATTGGCATTTTTG<br>AAATACTCC
pET15b/His-*TSA2-EPEA*	MGSSH_6SSGLVPRGSHM-Tsa2-<br>EPEA	**-**
pTrc99aNHis-ScTRX1	MH_6P-ScTrx1	**Ref. [27]**
pTrc99aNHis-ScTRR1	MH_6P-ScTrr1	**Ref. [27]**

Supplementary Table 7. Yeast strains used in this study.

Genotype	Source
BY4742 *MATα his3Δ1 leu2Δ1 lys2Δ0 ura3Δ0*	Euroscarf
BY4742 *TSA1*::*ROGFP2-TSA1*	This study
BY4742 *TSA1*::*ROGFP2-TSA1* Δ*tsa2*::*kanMX4*	This study
BY4742 *TSA2*::*ROGFP2-TSA2*	This study
BY4742 *TSA2*::*ROGFP2-TSA1* Δ*tsa1*::*kanMX4*	This study
BY4742wt +p416TEF roGFP2-Tsa1ΔC_PΔC_R + p415TEF Tsa2	This study
BY4742wt +p416TEF roGFP2-Tsa1ΔC_PΔC_R + p415TEF Tsa2ΔC_PΔC_R	This study
BY4742wt +p416TEF roGFP2-Tsa2ΔC_PΔC_R + p415TEF Tsa1	This study
BY4742wt +p416TEF roGFP2-Tsa2ΔC_PΔC_R + p415TEF Tsa1ΔC_PΔC_R	This study
BY4742wt +p416TEF roGFP2-*Hs*PRDX1ΔC_PΔC_R + p415TEF *Hs*PRDX2	This study
BY4742wt +p416TEF roGFP2-*Hs*PRDX1ΔC_PΔC_R + p415TEF *Hs*PRDX2ΔC_PΔC_R	This study
BY4742wt +p416TEF roGFP2-*Hs*PRDX2ΔC_PΔC_R + p415TEF *Hs*PRDX1	This study
BY4742wt +p416TEF roGFP2-*Hs*PRDX2ΔC_PΔC_R + p415TEF *Hs*PRDX1ΔC_PΔC_R	This study
BY4742wt +p416TEF roGFP2-BAS1AΔC_PΔC_R + p415TEF BAS1B	This study
BY4742wt +p416TEF roGFP2-BAS1AΔC_PΔC_R + p415TEF BAS1BΔC_PΔC_R	This study
BY4742wt +p416TEF roGFP2-BAS1BΔC_PΔC_R + p415TEF BAS1A	This study
BY4742wt +p416TEF roGFP2-BAS1BΔC_PΔC_R + p415TEF BAS1AΔC_PΔC_R	This study
BY4742wt +p416TEF roGFP2-*Li*PRDX1ΔC_PΔC_R + p415TEF *Li*PRDX2	This study
BY4742wt +p416TEF roGFP2-*Li*PRDX1ΔC_PΔC_R + p415TEF *Li*PRDX2ΔC_PΔC_R	This study
BY4742wt +p416TEF roGFP2-*Li*PRDX2ΔC_PΔC_R + p415TEF *Li*PRDX1	This study
BY4742wt +p416TEF roGFP2-*Li*PRDX2ΔC_PΔC_R + p415TEF *Li*PRDX1ΔC_PΔC_R	This study
BY4742wt +p416TEF roGFP2-BAS1A + p415TEF Empty	This study
BY4742wt +p416TEF roGFP2-BAS1A + p415TEF BAS1A	This study
BY4742wt +p416TEF roGFP2-BAS1A + p415TEF BAS1B	This study
BY4742wt +p416TEF roGFP2-BAS1B + p415TEF Empty	This study

BY4742wt +p416TEF roGFP2-BAS1B + p415TEF BAS1A	This study
BY4742wt +p416TEF roGFP2-BAS1B + p415TEF BAS1B	This study
BY4742wt +p416TEF roGFP2-*Li*PRDX1 + p415TEF Empty	This study
BY4742wt +p416TEF roGFP2-*Li*PRDX1 + p415TEF *Li*PRDX1	This study
BY4742wt +p416TEF roGFP2-*Li*PRDX1 + p415TEF *Li*PRDX2	This study
BY4742wt +p416TEF roGFP2-*Li*PRDX2 + p415TEF Empty	This study
BY4742wt +p416TEF roGFP2-*Li*PRDX2 + p415TEF *Li*PRDX1	This study
BY4742wt +p416TEF roGFP2-*Li*PRDX2 + p415TEF *Li*PRDX2	This study

Supplementary Note 1. Calculation of the number of possible decamer configurations

Unique decamers formed from two types of monomers, containing a given number of monomers of one type

If each position in a peroxiredoxin decamer can be occupied by one of two distinct monomer types (*A*, *B*), then there are 2^{10} = 1024 possible configurations, of which $\binom{10}{a}$ have exactly a monomers of type *A*. However, most of these configurations are equivalent under rotation around the 5-fold rotational symmetry axis perpendicular to the equatorial plane of the toroidal decamer (Fig. 1a), or the five 2-fold rotational symmetry axes in this plane and crossing the center of each dimer. To accurately enumerate the truly distinct decamer configurations, we apply the Cauchy–Frobenius lemma (a.k.a. Burnside's lemma [1]. This lemma asserts that the number of distinct configurations (N) of a symmetric object is given by the formula:

$$N = \frac{1}{|G|}\sum_{g\in G} Fix(g), \tag{1}$$

where G is the symmetry group of the object, which here includes rotations around the above-mentioned 5-fold and 2-fold axes; $|G|$ is the order of the group; and $Fix(g)$ is the number of configurations that are invariant under a given group element g.

In the present case, $|G| = 10$, of which one element is the identity operation, four are the 72°, 144°, 216° and 288° rotations around the 5-fold axis, and the remaining five are the 180° rotations around the five 2-fold axes.

We must now calculate $Fix(g)$ for each of these symmetry operations. Because every configuration is invariant under the identity operation (e), there are

$$Fix(e,a) = \binom{10}{a} \tag{2}$$

invariant configurations with exactly a *A*-type monomers.

In turn, a configuration is only invariant under rotation around the 5-fold axis if all five dimers are identical. This is only possible for $a = 0$ (1 configuration: all dimers *BB*), $a = 5$ (2 configurations: all *AB* or all *BA*), and $a = 10$ (1 configuration: all *AA*). Therefore:

$$Fix(g_{5\text{-fold}}, a) = 4 \times \begin{cases} 1 & a \in \{0,10\} \\ 2 & a = 5 \\ 0 & \text{otherwise} \end{cases}, \tag{3}$$

where the factor 4 accounts for the possible rotations around this symmetry axis.

Turning attention to rotations around each 2-fold axis, configurations invariant under this operation must meet both the following conditions: (i) dimers at which the axis is centered must be either *AA* or *BB*, and (ii) each monomer in dimers to one side of the axis must match another one of the same

type in the opposite side of the axis. This can only happen for even a, and since there are five independent *pairs* of monomers the number of such configurations is:

$$Fix(g_{2\text{-fold}}, a) = 5 \times \begin{cases} \binom{5}{a/2} & a\%2 = 0 \\ 0 & a\%2 = 1 \end{cases}, \qquad (4)$$

where the 5 factor accounts for the number of these axes.

Finally, the total number of configurations with exactly a *A*-type monomers is obtained by replacing equations (2)–(4), according to equation (1):

$$N(a) = \frac{1}{10}\left(Fix(e,a) + Fix(g_{5\text{-fold}}, a) + Fix(g_{2\text{-fold}}, a)\right). \qquad (5)$$

This yields the values described in the main text, with representations of the unique configurations in **Extended Fig. 2**.

Unique decamers formed of two types of monomers, each having n possible states

If there are two types of monomers and each can adopt n states, there is a total of $(2n)^{10}$ possible decamer configurations. In order to find how many of these are unique, we resort again to the Cauchy–Frobenius lemma (Supplementary Ref. 1), without the constraints on the number of monomers of a given type. In this case, all the $(2n)^{10} = 1024n^{10}$ configurations are invariant under the identity operation. For invariance under the 5-fold rotations all dimers must be equal, which $4 \times (2n)^2 = 16n^2$ configurations satisfy. In turn, for invariance under the 2-fold rotations, both monomers in one of the dimers must be of the same type and state ($2n$ choices), and each of the remaining two pairs of dimers must be identical ($(2n)^2$ choices per pair). Therefore, for the five 2-fold rotations a total of $5 \times (2n) \times (2n)^2 \times (2n)^2 = 160n^5$ configurations are invariant. The number of unique configurations is thus:

$$N(n) = \frac{1024n^{10} + 160n^5 + 16n^2}{10}, \qquad (6)$$

which is plotted in **Extended Fig. 2b**.

Supplementary References

1. Burnside, W. *Theory of Groups of Finite Order*, (Cambridge University Press, 1897).
