# Supplementary material for: Hetero-oligomerization drives structural plasticity of eukaryotic peroxiredoxins
Source: Nat Chem Biol. 2026 Mar 10;22(4):580–92. doi: 10.1038/s41589-026-02157-6 (PMC13038412; doi:10.1038/s41589-026-02157-6)

Source data for Figure1a

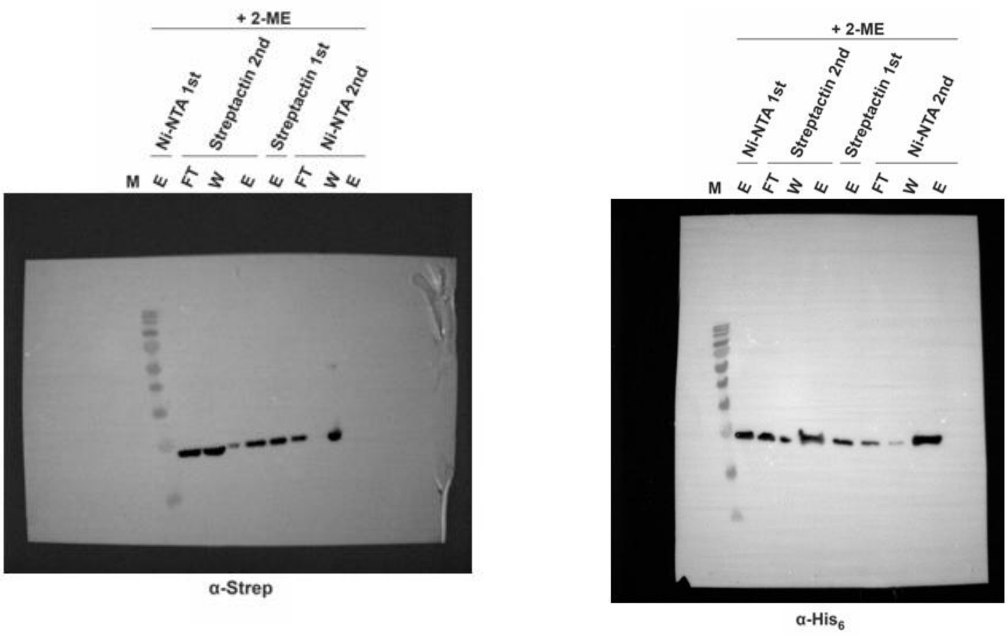

Source data for Figure 1b – left panels

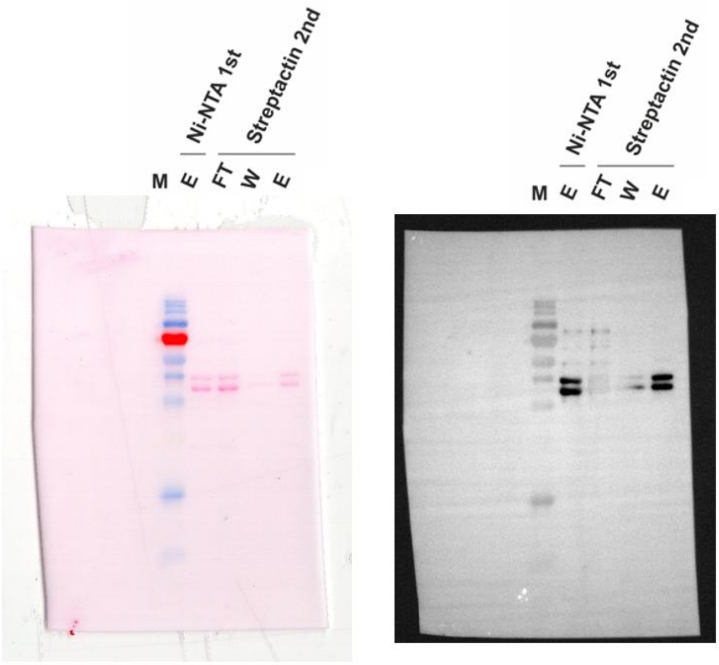

Source data for Figure 1b – right panels

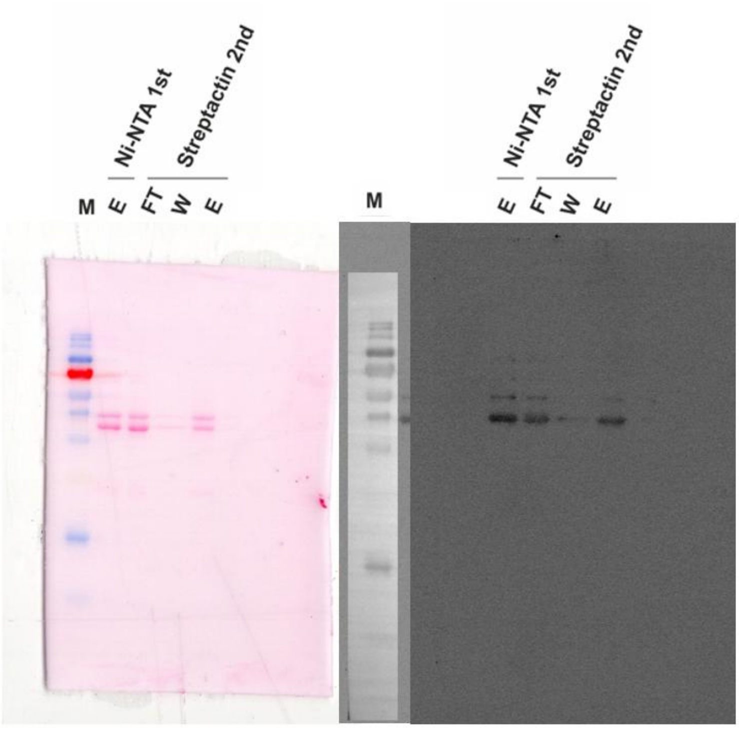

Supplement: Supplementary file 3 — Unprocessed gels. [file 41589_2026_2157_MOESM3_ESM.pdf]
