# Supplementary figures and images for: Hetero-oligomerization drives structural plasticity of eukaryotic peroxiredoxins
Source: Nat Chem Biol. 2026 Mar 10;22(4):580–92. doi: 10.1038/s41589-026-02157-6 (PMC13038412; doi:10.1038/s41589-026-02157-6)

Source Data for Figure 3b

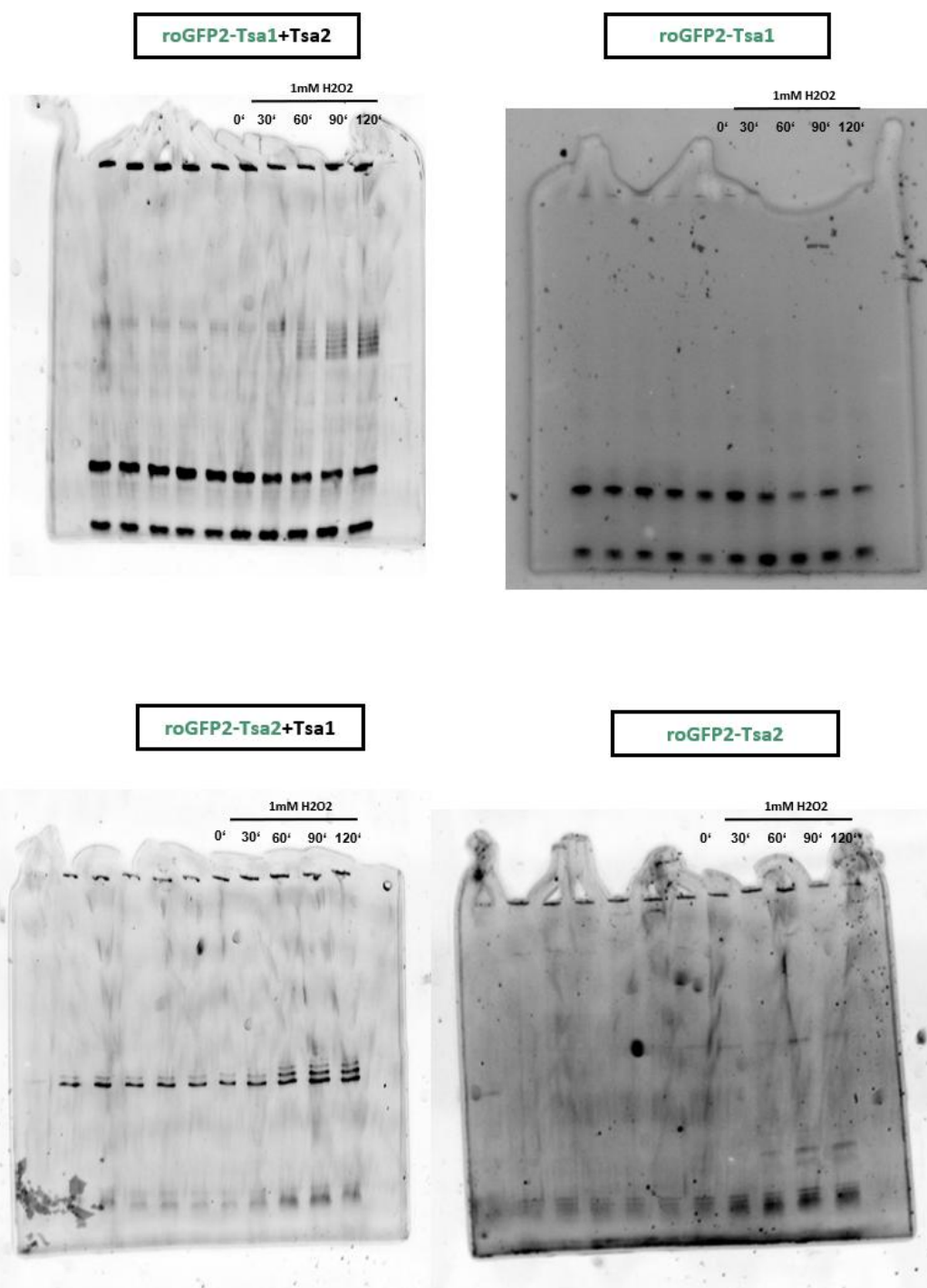

Supplement: Supplementary file 5 — Unprocessed gels. [file 41589_2026_2157_MOESM5_ESM.pdf]

Source Data for Figure 5a

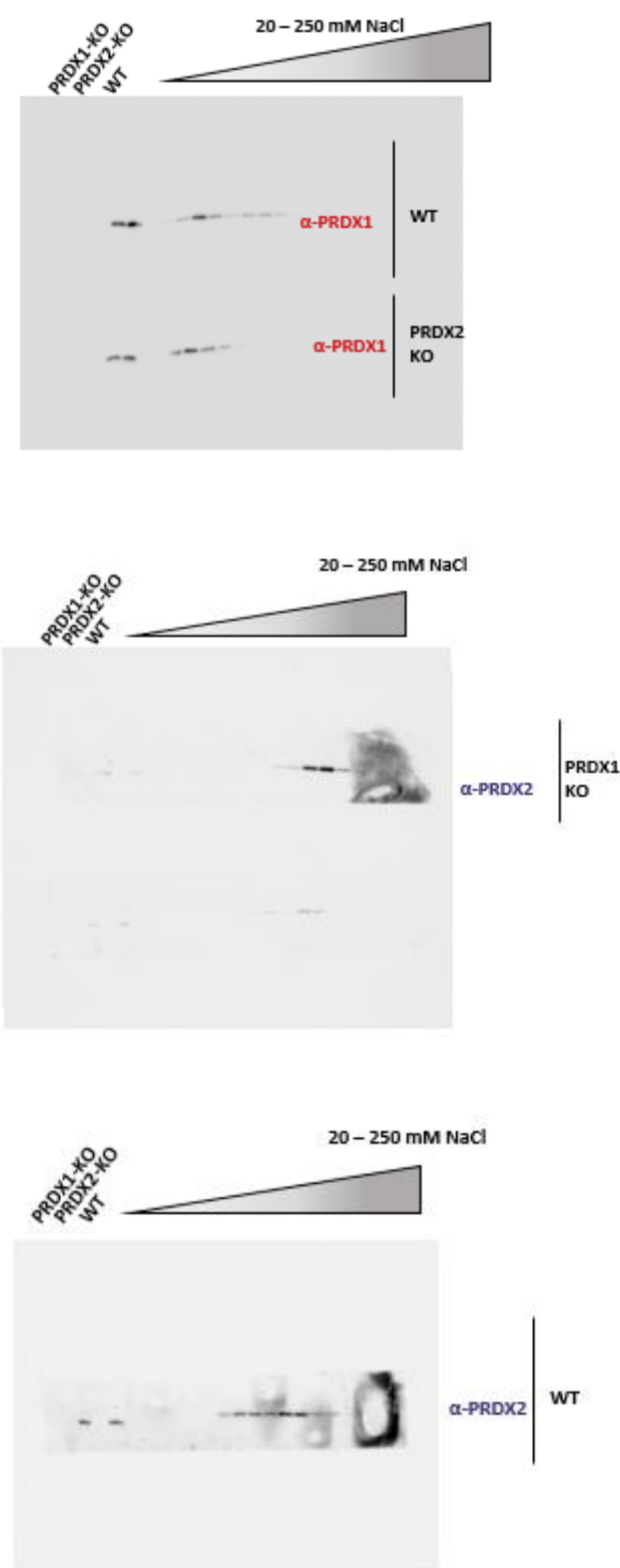

Supplement: Supplementary file 8 — Unprocessed gels. [file 41589_2026_2157_MOESM8_ESM.pdf]

Source Data for Figure 6a

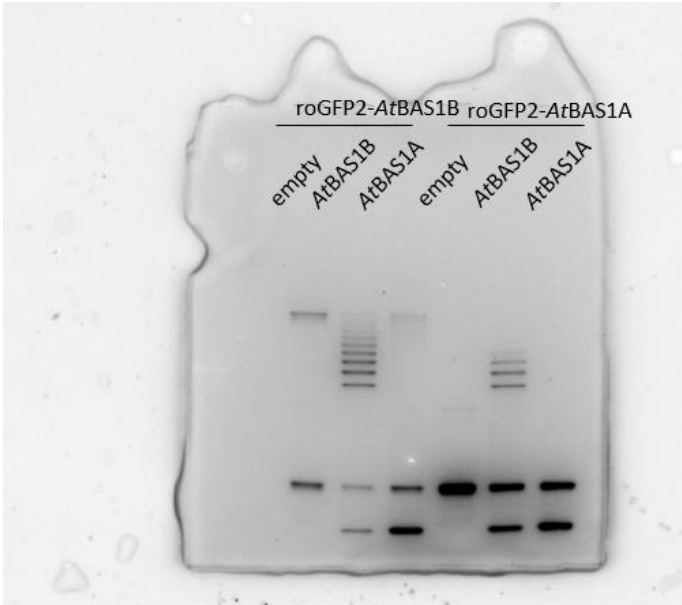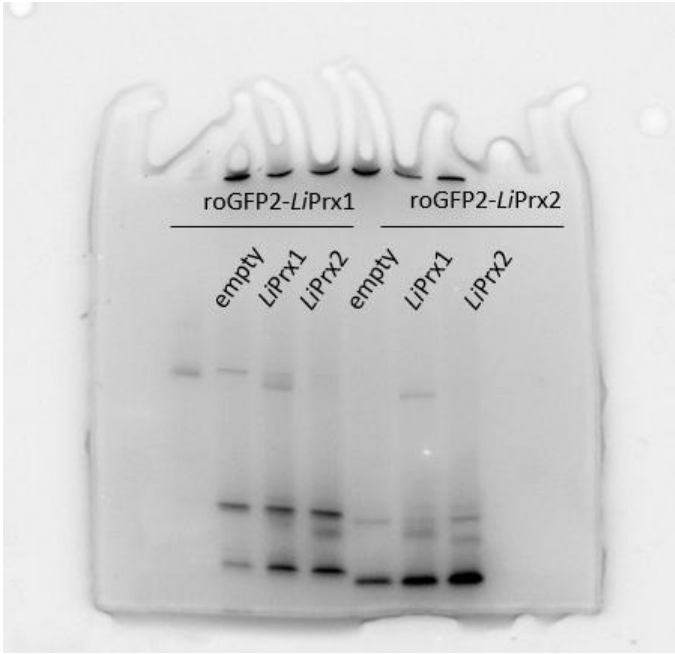

Supplement: Supplementary file 10 — Statistical source data. [file 41589_2026_2157_MOESM10_ESM.pdf]
